# Deciphering the high‐quality genome sequence of coriander that causes controversial feelings

**DOI:** 10.1111/pbi.13310

**Published:** 2020-02-05

**Authors:** Xiaoming Song, Jinpeng Wang, Nan Li, Jigao Yu, Fanbo Meng, Chendan Wei, Chao Liu, Wei Chen, Fulei Nie, Zhikang Zhang, Ke Gong, Xinyu Li, Jingjing Hu, Qihang Yang, Yuxian Li, Chunjin Li, Shuyan Feng, He Guo, Jiaqing Yuan, Qiaoying Pei, Tong Yu, Xi Kang, Wei Zhao, Tianyu Lei, Pengchuan Sun, Li Wang, Weina Ge, Di Guo, Xueqian Duan, Shaoqi Shen, Chunlin Cui, Ying Yu, Yangqin Xie, Jin Zhang, Yue Hou, Jianyu Wang, Jinyu Wang, Xiu‐Qing Li, Andrew H. Paterson, Xiyin Wang

**Affiliations:** ^1^ School of Life Sciences North China University of Science and Technology Tangshan Hebei China; ^2^ Center for Genomics and Computational Biology North China University of Science and Technology Tangshan Hebei China; ^3^ State Key Laboratory of Systematic and Evolutionary Botany Institute of Botany Chinese Academy of Sciences Beijing China; ^4^ University of Chinese Academy of Sciences Beijing China; ^5^ School of Genomics and Bio‐Big‐Data Chengdu University of Traditional Chinese Medicine Chengdu China; ^6^ Fredericton Research and Development Centre Agriculture and Agri‐Food Canada Fredericton New Brunswick Canada; ^7^ Plant Genome Mapping Laboratory University of Georgia Athens GA USA

**Keywords:** *De novo* coriander genome, tetraploid, genome evolution, terpenoid biosynthesis pathway, RNA‐Seq, metabolomics

## Abstract

Coriander (*Coriandrum sativum* L. 2*n* = 2*x* = 22), a plant from the Apiaceae family, also called cilantro or Chinese parsley, is a globally important crop used as vegetable, spice, fragrance and traditional medicine. Here, we report a high‐quality assembly and analysis of its genome sequence, anchored to 11 chromosomes, with total length of 2118.68 Mb and N50 scaffold length of 160.99 Mb. We found that two whole‐genome duplication events, respectively, dated to ~45–52 and ~54–61 million years ago, were shared by the Apiaceae family after their split from lettuce. Unbalanced gene loss and expression are observed between duplicated copies produced by these two events. Gene retention, expression, metabolomics and comparative genomic analyses of terpene synthase (TPS) gene family, involved in terpenoid biosynthesis pathway contributing to coriander’s special flavour, revealed that tandem duplication contributed to coriander TPS gene family expansion, especially compared to their carrot counterparts. Notably, a TPS gene highly expressed in all 4 tissues and 3 development stages studied is likely a major‐effect gene encoding linalool synthase and myrcene synthase. The present genome sequencing, transcriptome, metabolome and comparative genomic efforts provide valuable insights into the genome evolution and spice trait biology of Apiaceae and other related plants, and facilitated further research into important gene functions and crop improvement.

## Introduction

Coriander (*Coriandrum sativum* L. 2*n* = 2*x* = 22), also known as cilantro or Chinese parsley, is a globally important vegetable crop. Its global production tripled from 1994 to 2016, according to FAO (://faostat3.fao.org/), especially in Asia which accounts for 71.4% of global production. Its edible leaves and stems are widely used as vegetables, often referred to as cilantro, while its dried seeds can be used as a spice commonly called coriander. Coriander is native to the Mediterranean coast and central Asia, and now is cultivated globally (Zohary and Hopf, 2000; Zohary et al., 2012).

Coriander is rich in volatile oils that impart its distinctive aroma. The volatile oils contain mannitol, n‐acetaldehyde, furfural and linalool (Aelenei et al., 2019; Belsinger and Tucker, 2016; Zheljazkov et al., 2014). Cooking with coriander can increase flavour and eliminate the astringency of meat. Actually, volatiles are vital for various interactions with other organisms and the surrounding environment. According to their biosynthetic origins and chemical structures, plant volatiles are grouped into several classes, including terpenoids, benzenoids/phenylpropanoids, amino acid derivatives, carbohydrate derivatives and fatty acid derivatives (Dudareva et al., 2013; Sun et al., 2016). For example, the monoterpenes are well known as constituents of essential oils of aromatic plants and as components of floral scent, which are widely used in the food, cosmetic, perfume and pharmaceutical industries (Calo et al., 2015).

Coriander vegetative tissues (e.g. cilantro) are rich in nutrients and contain vitamin C, carotene, and vitamins B1 and B2 (Prachayasittikul et al., 2018). The amount of vitamin C in coriander is unusually high, with 7–10 g of leaves satisfying the body's demand (Abbassi et al., 2018; Verma et al., 2019). Coriander leaves contain more than 10 times higher concentrations of carotene than tomatoes, beans and cucumbers (Kandlakunta et al., 2008). Moreover, coriander has important medicinal value. Coriander stems and leaves can be used to increase appetite, comfort the stomach and improve digestion (Prachayasittikul et al., 2018), and coriander fruit exhibits gut modulatory, blood pressure lowering and diuretic activities (Jabeen et al., 2009).

Human populations are polymorphic for a qualitative difference in organoleptic response to coriander leaves, with the majority perceiving a tart, lemon or lime‐like flavour, but about 4%–14% think that coriander leaves taste like bath soap (Singletary, 2016). Actually, coriander originated from a Greek word, koris, which means a bad‐smelling bug. In a genetic survey of nearly 30 000 people, two genetic variants related to sputum perception were found, the most common of which were genes associated with infectious odours. Scientists found most coriander haters to have a common olfactory receptor gene called OR6A2, which absorbs the odour of aldehyde chemicals (Eriksson et al., 2012). Flavour chemists discovered that coriander aroma is produced by about six substances, most of which are aldehydes. Those who do not like the taste are sensitive to harmful unsaturated aldehydes, while those not able to detect aromatic chemicals find it pleasant. To avoid the soapy taste, the coriander haters can often eat parsley instead of coriander.

Coriander is from the Apiaceae family, which includes more than 3700 species in 434 genera, including well‐known crops such as carrot (*D. carota*) and celery (*Apium graveolens*). The Apiaceae family contains additional economically important plants, such as ajwain, angelica, anise, asafoetida, caraway and chervil (Feng et al., 2018; Que et al., 2019; Shelef, 2003). In recent years, a large number of studies have been reported on the phenotype, physiology, stress resistance, gene expression and metabolite identification of coriander (Abbassi et al., 2018; AlQuraidi et al., 2019; Choudhary et al., 2019; Divya et al., 2018; Fraser et al., 2017; Gholizadeh et al., 2018; Verma et al., 2019). However, among all the Apiaceae species, only the carrot genome has been sequenced until now (Iorizzo et al., 2016).

Here, we report a high‐quality genome assembly of coriander. The aims of the present research are to decipher important gene families controlling the aroma and flavour of coriander, to characterize the expression of these functional genes by using RNA‐Seq, metabolomics and comparative genomics analyses in coriander and carrot, and to understand the formation and evolution of the coriander genome.

## Results

### Genome de novo sequencing, assembly and annotation

**Figure 1:**
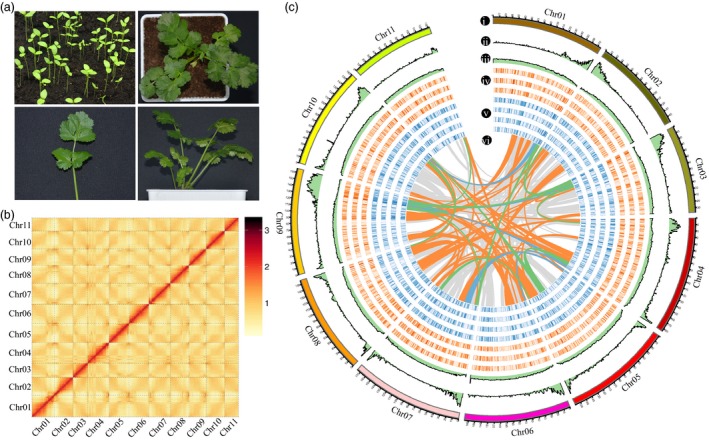
The morphology, Hi‐C map and chromosomal features of coriander genomes. (a) The morphology of coriander, including seedlings, top view, front view and leaf. (b) Hi‐C map showing genome‐wide all‐by‐all interactions between chromosomes. (c) i, 11 chromosomes of coriander, within 1‐Mb windows depicting; ii, gene density; iii, transposable element (TE) content; iv, gene expression levels (Log2FPKM) at 30, 60 and 90 days after sowing from outside to inside; v, gene expression levels (Log2FPKM) in flower, leaf, root and stem from outside to inside; and vi, lines connecting colinear blocks; orange, green and blue colours represent 20–40, 40–60 and ≥60 gene pairs in colinear blocks, respectively.

Here, the *C. sativum* L. (Coriander) genome was sequenced using a combination of several technologies (Figure 1a). We initially analysed the coriander genome by Kmer = 17, finding that the heterozygosity rate was 0.47%, the repeat sequence ratio was 80.58%, and the estimated genome size was 2130.29 Mb (Figure S1, Tables S1‐S2). A PacBio platform (Sequel I) was used to produce a total of 197.45 Gb sequencing data with average coverage depth of 92.69× (Figure S2). In addition, a 10X Genomics library of second‐generation small fragments was constructed and sequenced using Illumina platform HiSeq 4000. A total of 577.88G of coriander DNA sequence were produced with a depth of 271.27X (Table S3). Then, a coriander genome sequence was *de novo* assembled, with cumulative scaffold length of 2147.13 Mb and scaffold N50 length of 2.15 Mb (Tables S4‐S7).

We conducted Hi‐C analysis to assist the genome assembly and eventually obtained 278.90 Gb high‐quality sequences (Table S8, Figure S3). A Hi‐C heat map could separate distinct regions on different chromosomes (Figure 1b). Although the revised assembly was slightly smaller (2118.31 Mb), contig N50 length reached 604.13 Kb and scaffold N50 reached 160.99 Mb with only 7 scaffolds achieving N50 (Table 1, Tables S9‐S10). Grossly, we obtained a high‐quality assembled genome. Among 33 representative plant species recently sequenced, this value was second only to *Papaver somniferum* (Guo et al., 2018; Table S11).

**Table 1:** Results of Hi‐C auxiliary assembly of the *C. sativum* genome

Sample ID	Length		Number	
	Contig† (bp)	Scaffold (bp)	Contig†	Scaffold
Total	2 118 309 730	139.8	9936	6186
Max	3 580 399	184 508 978	–	–
Number ≥ 2000	–	–	9711	5961
N50	604 128	160 995 510	1031	7
N60	464 580	156 618 565	1432	8
N70	345 039	149 113 063	1957	9
N80	233 760	132 297 589	2696	11
N90	115 576	176 899	3961	238

^†^ Assembled scaffolds > 100 bp.

By implementing *de novo* repeat prediction tools with reference to the existing Repbase library, we found that 70.59% of the coriander genome is comprised of repetitive sequences, 1.5 times that in carrot (46%) (Figure 1c, Tables S12‐S13, Figure S4). Most transposable elements (TEs) belong to the long terminal repeat (LTR) category, with total length over 1.4 Gb, accounting for 66.71% of the whole genome. Among each kind of LTRs, the two most frequent types were Copia and Gypsy, respectively, accounting for 55.85% and 36.47% of all LTRs.

In total, 40 747 high‐quality genes were predicted in the coriander genome and comparative analysis with 7 other species (Tables S14‐S15). Most genes were distributed in the terminal regions of chromosomes (Figure 1c). Functional annotation using several protein databases, including NCBI nonredundant protein (NR), Swiss‐Prot, KEGG and InterPro (Table S16), provided evidence of function for 37 772 (92.7% of) genes, with 25 722 annotated by all four databases (Figure S5a). In addition, we identified 1.65 Mb noncoding RNAs, including miRNA, tRNA, rRNA and snRNA, accounting for 0.078% of the genome assembly (Table S17).

**Figure 2:**
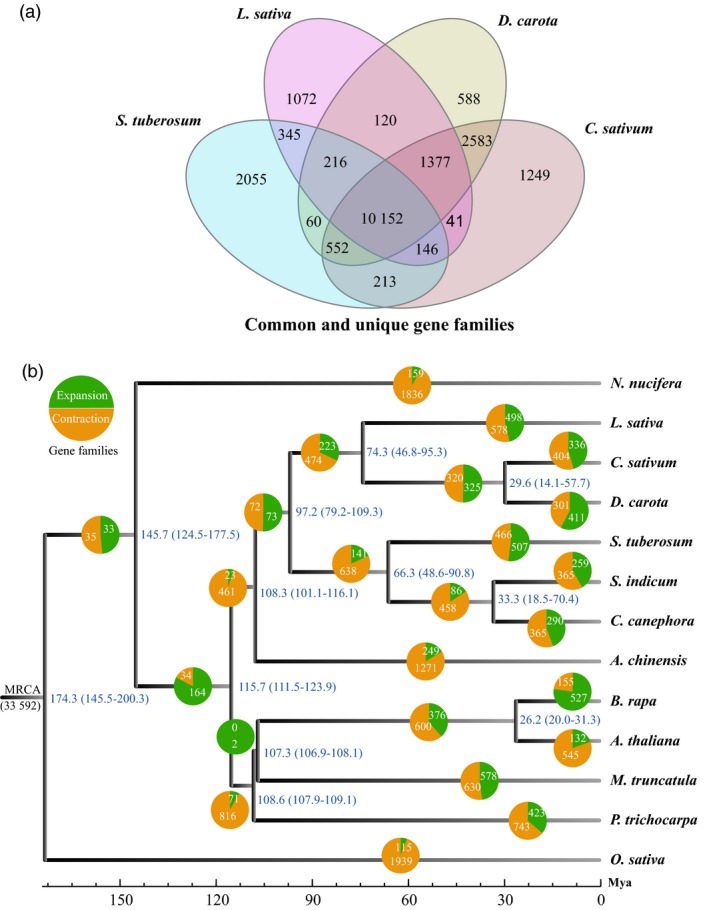
Coriander gene family analyses. (a) Common and lineage‐specific gene families in carrot (*D. carota*), potato (*S. tuberosum*), lettuce (*L. sativa*) and coriander (*C. sativum*). (b) Divergence time estimation and gene family expansion/contraction analyses. The numbers on the nodes represent the divergence time of the species (million years ago, Mya), with confidence range in brackets. The green and orange pies indicate the gain (expansion) and loss (contraction) number of gene families.

In a comparative analysis with 12 representative species, we found coriander to had 1249 specific gene families. In all 13 species, there were 33 601 gene families in total, including 6548 multiple‐gene families and 519 single‐copy ones (Figure S5b). Notably, the Asterid relatives, carrot, potato (*S. tuberosum*), lettuce (*L. sativa*) and coriander, shared a total of 10 152 gene families (Figure 2a). An analysis using CAFÉ showed that the MRCA (most recent common ancestor) of these 13 species had 33 592 gene families (Figure 2b). Coriander has lost more gene families (404) than it gained (336). In contrast, carrot has gained more gene families (411) than it lost (301).

To clarify the inter‐related evolutionary histories of these 13 plants, we performed multiple sequence alignments of all single‐copy genes, combined the results to construct a super alignment matrix and then built a phylogenetic tree by a maximum likelihood method using RAxML software (Figure 2b). The times of species divergence were estimated by using this phylogenetic tree. Using 519 single‐copy gene families and MCMCTree in the PAML software package, we performed time correction utilizing the divergence time between five known species such as coriander and *Medicago truncatula* (see Methods section), inferring that coriander and carrot separated 29.6 Mya (14.1–57.7 Mya) (Figure 2b).

### Genome organization and polyploidization

**Figure 3:**
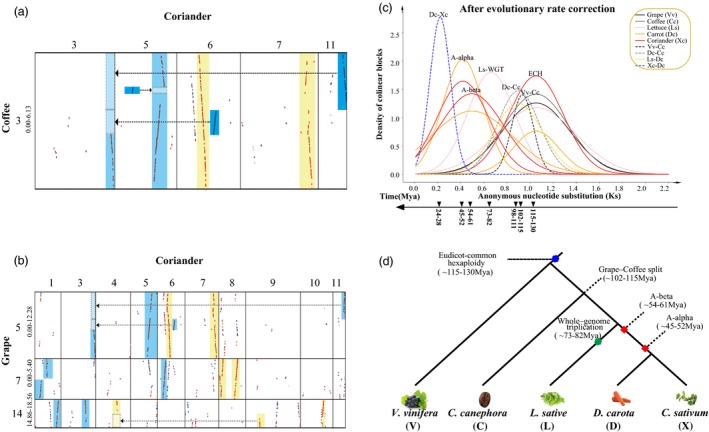
Homologous gene dot plots between genomes and evolutionary dating. (a,b) Homologous gene dot plots among (a) grape vs. coriander; (b) coffee vs. coriander. Grape, coriander and coffee chromosome numbers are shown on the tops and sides of plots, segment regions showed in Megabases (Mb). Dot colours indicate best hit (red), secondary hits (blue) and others (grey). Arrows show complementary correspondence produced by chromosome breakages during evolution. Yellow and blue blocks represent different WGD, respectively. (c) Corrected synonymous nucleotide substitutions (Ks) among colinear genes. Lines show Ks distribution within (continuous) and between genomes (dashed). Distributions fitted by using corrected Ks values were used to infer evolutionary dates. (d) Species and gene phylogenetic trees for coriander (X), carrot (D), Lettuce (L), coffee (C) and grape (V): eudicot‐common hexaploidy (ECH) denoted by blue hexagon, Asteraceae‐common hexaploidy (ACH) by green hexagon and the two Apiaceae palaeo‐tetraploidizations by red squares.

Besides a hexaploidization event shared with major eudicot plants, ECH (Jaillon et al., 2007), we identified two tetraploidization events affecting the coriander genome. Firstly, by inferring gene colinearity, we identified 796 homoeologous blocks within the coriander genome, involving 7214 colinear gene pairs and 9286 genes (Tables S18, S19). In recursive genomic duplications, one gene can be involved in multiple colinear gene pairs and homoeologous blocks. Secondly, by inferring intergenomic gene colinearity, we mapped coriander genome sequences onto grape, coffee, lettuce and carrot genomes (Tables S18, S19). The estimated synonymous divergence level (Ks) and complementary breakage points shared by colinear blocks help to infer and relate coriander homoeologous blocks to different polyploidization events. The best‐matched or likely orthologous correspondence ratios of grape, coffee, lettuce and carrot with coriander are 1:4, 1:4, 3:4 and 1:1 (Figure 3a, b, Figures S6‐S10), indicating that, after splitting from the Asterales, the Apiaceae experienced additional polyploidization events, resulting in overlapping coriander homoeologous regions often up to 4× depth (Figure 3c).

With grape as the reference, we classified the 4x homoeologous regions into two groups based on Ks (Table S20), each containing 567 and 435 colinear gene pairs. These two groups of colinear gene blocks covered 80.3% and 79.6% of the genome, respectively. With coffee as the reference, based on Ks, we also classified the 4x homoeologous regions into two groups (Table S21), each containing 664 and 547 colinear gene pairs. These two groups of colinear gene blocks covered 82.4% and 81.1% of the genome, respectively. These findings indicated that there were likely two whole‐genome duplication, or tetraploidization, events. With carrot as the reference, homoeologous regions had 1:1 relationship with coriander (Table S22), indicating that the two whole‐genome duplications predated the carrot–coriander split. Divergent evolutionary rates were found among the plants studied, and we adopted a correction‐by‐shared‐event approach to gauge the dates of the two Apiaceae family‐common events, respectively, dated to ~45–52 and ~54–61 Mya, now referred as A‐alpha and A‐beta (Figure 3c, d, Figure S11, Tables S23‐S24). Accordingly, the divergence of coriander and carrot was inferred to occur 24–28 Mya, overlapping phylogenetically inferred date above.

**Figure 4:**
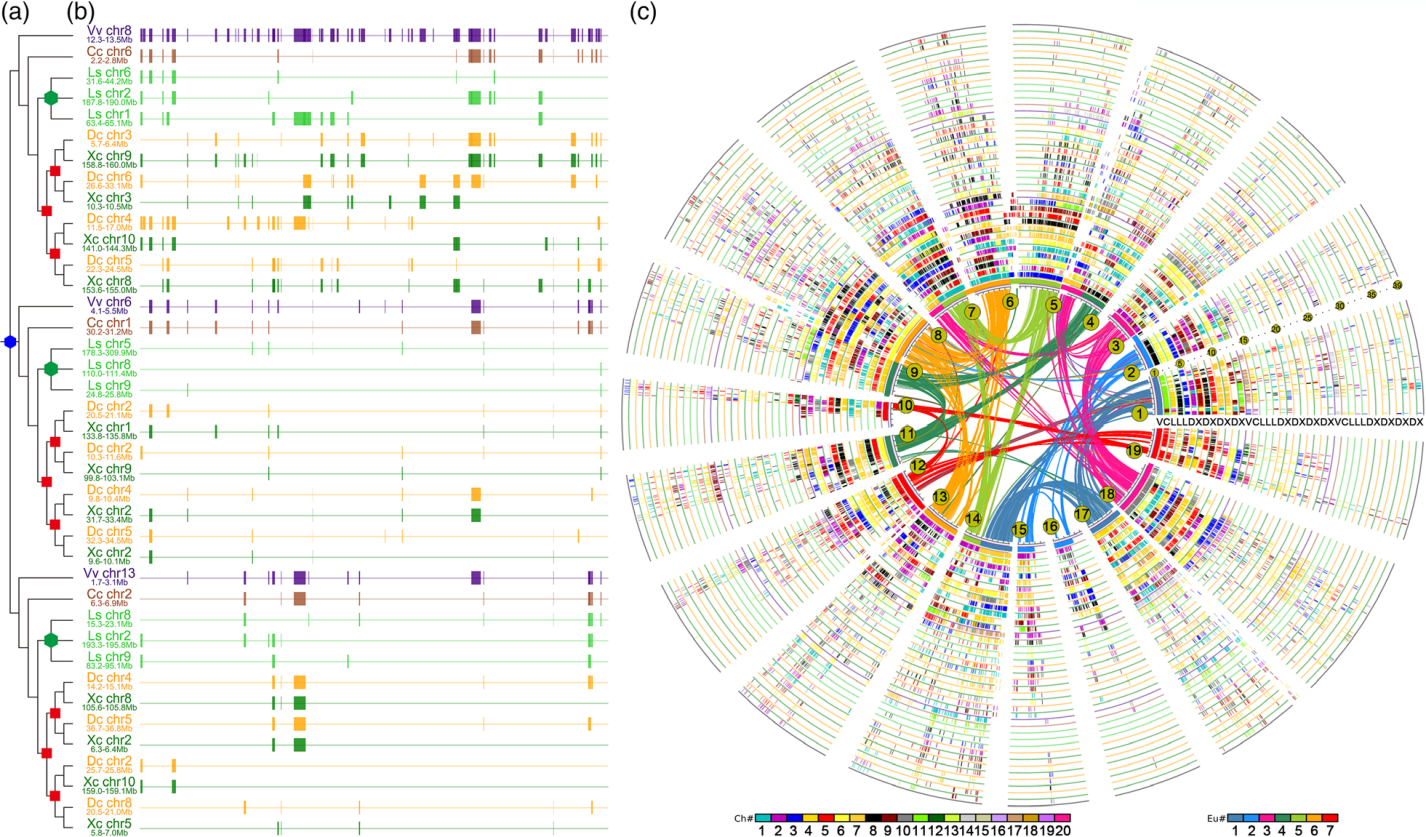
Local and global alignment of genomes. (a) A gene phylogenetic tree was constructed using colinear genes among coriander, carrot, lettuce, coffee and grape. Hexagons indicate eudicot‐common hexaploidy (ECH, blue), Asteraceae‐common hexaploidy (ACH, green) and the Apiaceae palaeo‐tetraploidization events, A‐beta and A‐alpha (red). (b) Local alignment of genes among coriander (Xc), carrot (Dc), lettuce (Ls), coffee (Cc) and grape (Vv). Using as reference a homoeologous series of grape segments produced by the ECH, we displayed the alignment of a region from 12.3 to 13.5 Mb on grape chromosome 8, 4.1 to 5.5 Mb on chromosome 6 and 1.7 to 3.1 Mb on chromosome 13, along with corresponding regions from other genomes. Chromosome numbers follow the names of plants, and locations on chromosomes are below. Gene (rectangle) positions correspond to those of colinear grape genes. (c) Global alignment of homologous regions in coriander (X), carrot (D), lettuce (L) and coffee (C) genomes with grape (V) as a reference. Genomic paralogy, orthology and outparalogy information within and among genomes are displayed in 39 circles, each corresponding to an extant gene. The curved lines within the inner circle are formed by 19 grape chromosomes colour‐coded to correspond to the 7 ancestral chromosomes before the major eudicot‐common hexaploidy (ECH). The short lines forming the innermost grape chromosome circles represent predicted genes, which have 2 sets of paralogous regions, forming another two circles. Each of the three sets of grape paralogous chromosomal regions has one ortholog in coffee, 3 in lettuce and 4 in coriander and carrot. The resulting 39 circles were marked according to species by a capital letter. Each circle has an underline coloured as to its source plant, corresponding to the colour scheme, and each circle is formed by short vertical lines that denote homologous genes, coloured as to chromosome number in their respective source plant as shown in the inset colour scheme.

Unbalanced fractionation of subgenomes was consistent with a tetraploid nature of both Apiaceae family‐common events. Alignment of studied genomes permitted inference about gene retention after each tetraploidization (Figure 4, Figures S12‐S13). Two Apiaceae plants, coriander and carrot, shared 14 255 colinear orthologous genes, accounting for 42.01% and 46.37% of their respective predicted gene sets, much more than either share with non‐Apiaceae plants (Tables S25‐S27). Given no gene loss or translocation, one grape or coffee gene would have 4 coriander orthologs; however, we found this in only 0.12% of cases, with 20.76%, 6.76% and 1.39% having 1, 2 or 3 orthologs, respectively, showing that 70.97% of coriander genes have been deleted from the orthologous regions (Figure S14, Table S28). Further, alternative erosion of grape–coriander colinear orthologs showed that 5765 and 6632 genes, respectively, were removed from the orthologous regions between A‐beta and A‐alpha, and after A‐alpha. Comparatively, only 1307 genes were inferred to have been removed after the core‐eudicot‐common hexaploidization (ECH) and before A‐beta. The chromosome regions duplicated by A‐beta and A‐alpha often have divergent gene retention levels (Tables S29‐S33, Figures S15‐S17). No matter whether grape or coffee was used as reference, the divergent gene retention pattern holds true in both carrot and coriander, showing the tetraploid nature of Apiaceae family‐common genome duplications. Genes were removed from colinear blocks followed by random geometric distribution at large, that is, if DNA breakage occurred, a segment of DNA of a certain length including neighbouring genes was likely removed (Figures S15‐S17). For example, with grape (or coffee) as reference, we inferred that 87.05% (82.38%) of genes were removed in segments containing <10 neighbouring genes, respectively.

Phylogeny reconstruction with colinear genes supports the inference of the two Apiaceae tetraploidization events. We constructed 524 and 683 groups of homologous gene evolutionary trees, each containing at least two carrot genes and at least two coriander genes, with one grape gene and one coffee gene as outgroups. In the homologous gene trees with grape and coffee as the outgroup, 78.1% (409/524) and 76.9% (525/683) respectively correspond to the expected topology (Figure S18).

### Expression bias between two subgenomes

To explore coriander gene expression, we selected four tissues (root, stem, leaf and flower) for RNA‐Seq analyses. A total of 529 212 394 clean reads and 79.36 Gb sequencing data were obtained for the four tissues (Table S34). In addition, the coriander and carrot samples were collected from three different growth stages, including 30, 60 and 90 day after sowing. A total of 69.22 and 68.70 Gb sequencing data were obtained for each plant, respectively (Table S35). High correlations were obtained for three replications of these samples (Figure S19). The total mapped reads from four tissues onto the coriander genome sequence were more than 90%, and the uniquely mapped ratios were more than 80% (Table S36). Similar mapped ratios were found for the three different growth stages of each plant (Tables S37‐S38). A total of 39 225 (90.74% of) coriander genes showed expression in at least one tissue, while 4005 genes had no expression in all four tissues (Table S39, Figure S20). A total of 35 759 (83.93%) coriander genes were detected in at least one developmental stage, and 6848 genes had no expression in any of the three developmental stages (Table S40). In carrot, a total of 28 667 (82.10%) genes showed expression, and 6251 genes had no expression in any of the three developmental stages (Table S41).

Unbalanced gene expression was observed between duplicated copies of chromosomes produced in A‐beta and A‐alpha, further supporting their tetraploid nature (Table S42). As to the A‐beta duplicated chromosome copies, 85.45%–94.75% of duplicated genes show differences in expression using coffee as reference and 82.61%–93.70% using grape as reference. Likewise, for A‐alpha duplicated chromosome copies, 75.68%–97.73% of duplicated genes show difference in expression with coffee as reference and 78.43%–100% with grape as reference. For tetraploidization events, the significantly diverged expression homologs often have the higher expressed copies concentrated on one set of accumulated chromosomal region(s), not balanced across two sets. Moreover, we compared the expression level of coriander genes in colinearity to the grape ones. We checked likely expression difference between coriander genes each having just one‐copy duplicated regions and those ones each preserving two duplicated copies. Unexpectedly, we found that the one‐copy group had significantly lower expression than the average expression of the two‐copy group. This finding is consistent in different tissues, with t‐test P‐values from 2.0e‐05 to 3.6e‐02 in four tissues (Table S43).

### Determination of functional genes and related volatile aroma compounds

#### Terpenoid biosynthesis pathway

The terpenoid biosynthesis pathway is related to the formation of plant volatile substances, imparting aroma. The pathway mainly contained eight subpathways in Arabidopsis according to the KEGG. We identified 159 Arabidopsis genes from these subpathways and used these as seeds to predict homologous genes in coriander and 5 other representative plants by using BLASTP (*e*‐value < 1e‐5, identify > 50%, score >200) (Table S44).

**Figure 5:**
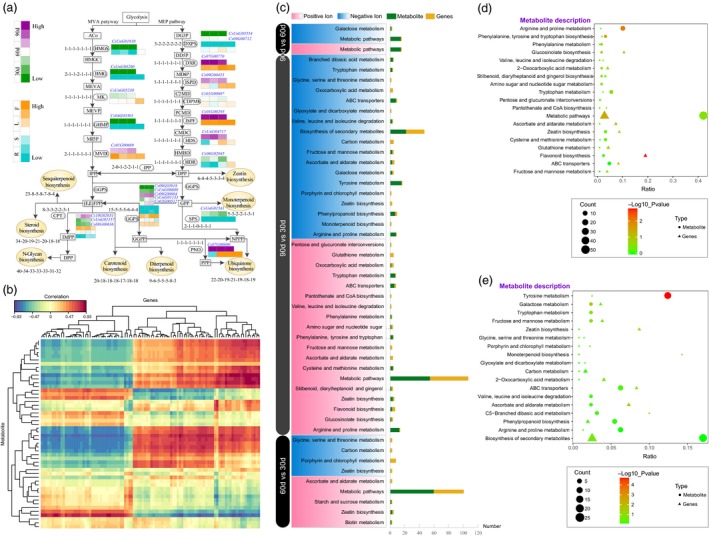
Inferred terpenoid biosynthesis genes and metabolic profile analyses of coriander at 3 development stages, including 30, 60 and 90 days after sowing. (a) Terpene precursors are synthesized by two main pathways, the mevalonic acid (MVA) and the methylerythritol phosphate (MEP) pathway. A total of 7 plants (Arabidopsis, coriander, carrot, lettuce, tomato, grape and Amborella) were compared. The notation ‘1‐1‐1‐1‐1‐1‐1’ indicates one homologous gene was identified among each plant, respectively. Gene expression was detected in the different tissues (R: root; S: stem; L: leaf; F: flower) and at different developmental stages (30, 60 and 90 days) of coriander. The purple and orange colours indicate the high expression level at different developmental stages and tissues, respectively. The orange oval represents 8 sub‐biosynthesis pathways. The abbreviations of each gene encoding enzymes and compounds are listed in Table S46. Gene expression levels in these biosynthesis pathways are shown in Tables S47‐S48 and Figures S21‐S22. (b) The association analyses between transcriptome and metabolome data. The red and blue colours indicate the positive and negative correlation between metabolite and genes, respectively. (c) The significant enrichment of terms between 60 vs 30 days, 90 vs 30 days and 90 vs 60 days of coriander. The red and blue colours indicate the positive and negative ion identified by metabolic profiles, respectively. The green and orange colours indicate the metabolite and genes number located in the related enrichment terms. (d, e) The plot of enrichment terms obtained by the association of transcriptome and metabolome data.

Using 44 coriander genes in the terpenoid backbone biosynthesis pathway, we inferred the homologous genes with Arabidopsis in other 6 representative plants (Table S45). Almost every node in the regulatory pathway has one or more gene copies among the 7 species. No isopentenyl diphosphate (*IPP*) gene was found in coriander (Table S45). Certain genes had high expression in all three development periods and 4 tissues, such as *Cs07G00770.1* (homologous to the Arabidopsis *DXR* gene encoding 1‐deoxy‐d‐xylulose 5‐phosphate reductoisomerase in MEP pathway), *Cs01G00293.1* (homologous to the Arabidopsis *ISPF* gene (isoprenoid F) in MEP pathway) and *Cs01G00669.1* (homologous to the Arabidopsis *MVD1* gene encoding a mevalonate diphosphate decarboxylase in MVA pathway; Figure 5a). Some genes showed tissue‐specific expression, for example, *Cs07G00690.1* (homologous to the Arabidopsis *PNO* gene encoding a pyridine nucleotide‐disulphide oxidoreductase) is not expressed in the root, while had high expression in the other 3 tissues. In addition, we detected the expression of 113 coriander genes in eight subpathways (Tables S46‐S47; Figures S21‐S22).

#### Volatile aroma compound identification by metabolic profile

To investigate the composition and content of metabolites in the coriander, we performed a metabolome analysis for 3 development stages, including 30, 60 and 90 days after sowing of coriander. Based on the LC‐MS detection and the bioinformatics analyses, more than thousands of metabolites were identified, such as aroma compounds linalool, delphinidin, rutin, kaempferol and daidzein. The metabolome analysis results showed that the higher content of metabolites is mainly 3‐desoxy‐3,4‐methylenedioxy pyrovalerone, choline, ethyl nicotinate, scropheanoside I, α, α‐trehalose, etc. (Table S49).

To make a connection between the biosynthetic genes and the aroma‐related metabolite, we conduct the comparative, association and enrichment analyses of transcriptome and metabolic profile data for 3 development stages (30d, 60d and 90d) of coriander (Figure 5, Table S50). We can identify the metabolite‐related genes by the correlation analyses between transcriptome and metabolome (Figure 5b). A total of 9, 37 and 3 significant enrichment of terms between 60 vs 30 days, 90 vs 30 days and 90 vs 60 days were identified, respectively (Table S50, Figure 5c). Therefore, there were more significant differences of 90 vs 30 days than other two comparative pairs. The mainly enrichment terms were metabolic pathways, biosynthesis of secondary metabolites and tyrosine metabolism (Table S50, Figure 5c, d, e).

#### Terpene synthase (TPS) gene families

**Figure 6:**
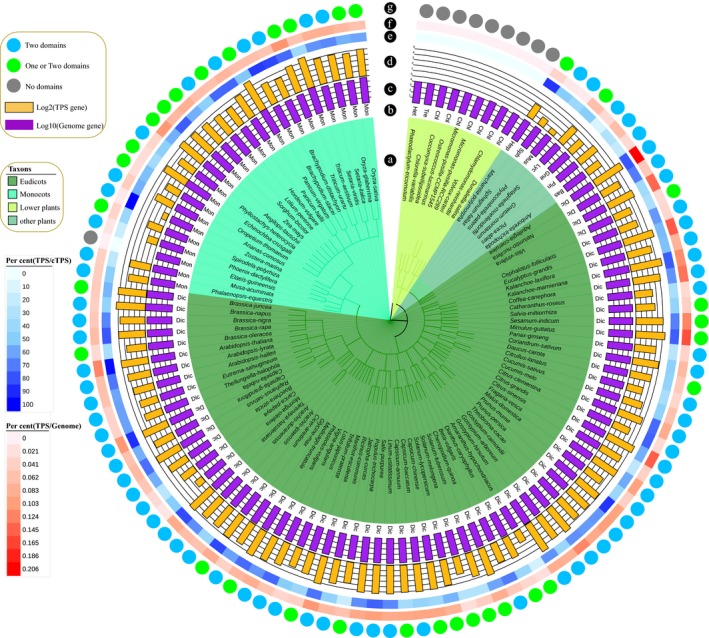
Comparative analysis of the TPS gene family of coriander and 112 representative plants, including eudicots, monocots, lower plants and other plants. Colours indicate that all TPS genes of one species contain two domains (blue); TPS genes of one species contained one or two domains (green); or there were no TPS genes (grey). The orange and purple colours represent the TPS genes and genome gene numbers transformed by log2 and log10, respectively. The gradient from blue to white represents the percentage of TPS/cTPS (candidate TPS). The gradient from red to white represents the percentage of TPS/Genome genes.

Because it is vital to terpenoid biosynthesis, we systematically identified the TPS gene family members in coriander and 112 representative plants, including lower plants, bryophytes, ferns, gymnosperms and angiosperms. Among 4711 candidate TPS genes identified in all these plants (Figure 6, Table S51), further screening prioritized 2487 TPS genes for the following analysis. Among them, no TPS gene was identified in the 9 lower plants, indicating distinct gene contents in terpenoid regulation and synthesis from higher plants. Of 104 higher plants, no TPS gene was found in the monocot *Elaeis guineensis*.

Among the 103 species containing the TPS gene, 70 (67.96%) had two domains (PFAM IDs: PF03936 and PF01397). Among the remaining 33 species, some TPS genes contain only one domain, and the number of genes containing a single domain in different species accounts for 1.75%–33.4%. Of the 2487 genes identified above, 2442 had both domains, 13 contained only PF03936, and 32 contained only PF01397 domain.

**Figure 7:**
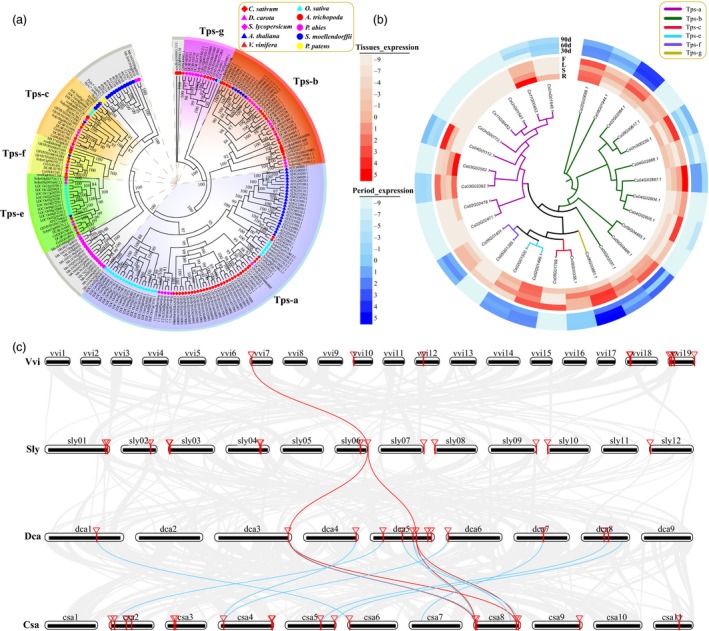
TPS gene family analyses. (a) Phylogenetic analysis of coriander and nine representative plant TPS gene families. (b) TPS gene expression in 4 different tissues (R: root; S: stem; L: leaf; F: flower) and at 3 different developmental stages after sowing (30, 60 and 90 days) of coriander. (c) Colinear analysis of TPS genes in coriander and other 3 representative species (Csa: coriander; Dca: carrot; Sly: tomato; Vvi: grape).

To further investigate the TPS gene family, we selected 10 representative species containing a total of 215 TPS genes, with the most copies found in grape (35), followed by Arabidopsis and rice (33) (Figure 7). There are only 2 and 8 in *P. patens* and *A. trichopoda*, respectively (Table S51). Twenty‐nine TPS genes were identified in coriander, but only 16 in carrot. We found that 6/6, 2/1, 14/6 and 3/3 of these coriander/carrot genes could be related to dispersed, proximal, tandem and whole‐genome duplication (WGD) type of genes. Thus, tandem duplication is responsible for most TPS gene expansion in coriander, after its split from carrot.

Based on phylogenetic tree topology and the reported classification of grape and Arabidopsis TPS genes (Aubourg et al., 2002; Martin et al., 2010)**,** we divided the coriander TPS genes into seven groups, namely TPS a‐g (Figure 7a). The TPS‐a group contains more genes, while TPS‐f and TPS‐g groups contain few genes. These three groups of genes, derived from *A. trichopoda*, *P.* *abies*, *S.* *moellendorffii* and *P. patens,* were not attributed to any group. We found that in the same group, the TPS genes of the same species were likely to be clustered together, indicating that new gene copies were often produced by gene duplication after species divergence.

Gene expression analysis showed that more coriander genes had higher expression in flower than in other 3 tissues studied (Figure 7b). Two genes, *Cs11G00453.1* and *Cs03G02562.1*, had higher expression in roots than in other 3 tissues, and both belonged to the TPS‐a group; gene *Cs06G00661.1* had higher expression in all examined tissues except the root. Several TPS genes were highly expressed at each of 3 developmental stages, such as *Cs02G02594.1* and *Cs06G00661.1*, while 11 genes had no expression at any of the three stages. Notably, one TPS‐g group gene, *Cs06G00661.1*, had high expression in different tissues and different development stages and may play important roles in encoding linalool synthase and myrcene synthase according to a previous report (Aubourg et al., 2002).

In addition, colinearity analyses for the TPS gene family (Figure 7c) showed that one grape gene and its orthologs preserved their colinear orthologs among four species: the *GSVIVT01001155001* gene in grape, the *Solyc06g084240.1.1* gene in tomato, the *DCAR_012483* and *DCAR_01842* genes in carrot, and *Cs08G00356.1* and *Cs08G01799.1* genes in coriander.

## Discussion

We have produced a high‐quality coriander reference genome with the latest sequencing technologies and bioinformatics methods, studied the coriander genome evolution and identified the candidate genes of the underlying biology for its controversial flavour of coriander. The coriander genome, combined with the carrot genome, will represent the essential resources for the Apiaceae community and in particular for coriander breeders.

The Apiaceae palaeo‐polyploidization events were reported but not well resolved about their ploidy levels so far (Iorizzo et al., 2016). Here, we inferred two tetraploid events, shared by coriander and carrot, after divergence from the lineage leading to asterales (e.g. lettuce). These two tetraploidization events probably provided a high level of hybridity, empowering fast divergence of the ancestral plant(s), eventually to produce the Apiaceae family of more than 3700 species.

The molecular basis of coriander scent and the related regulatory network has not been well described until now. Here, we analysed terpenoid biosynthesis pathway in coriander and related plants, related to the formation of plant volatile substances. Furthermore, we identified the TPS gene family in coriander and other plants. Interestingly, we found one TPS‐g group gene (*Cs06G00661.1*) to have high expression in different tissues and development stages, showing its likely important roles in encoding linalool and myrcene synthase (Aubourg et al., 2002).

The high‐quality coriander genome sequence described here, in combination with comparative transcriptome and metabolomics analysis, identified related genes in coriander and explored their expression between different tissues and development periods, laying a solid foundation for dissecting the genetic mechanisms regulating aroma and flavour accumulation in plants, with potential application to crop breeding.

## Materials and Methods

### Genome sequencing

Leaf samples were collected from *C. sativum* ‘SJ01’ and processed for genomic DNA isolation and library construction. The following three sequencing strategies were used: (a) second‐generation library construction included 2 paired‐end Illumina libraries (Illumina Inc, CA, USA) with 350 bp fragments. A total of 139.87Gb clean data were obtained, which covered the genome ~65.66×. The abundance of 17 nt k‐mers was used to estimate the genome size (Marcais and Kingsford, 2011). (b) Third‐generation library construction followed the PacBio SMRT protocol and was sequenced on the PacBio Sequel platform (Pacific Biosciences, CA, USA). A total of 197.45Gb clean data were obtained, which covered the genome ~92.69×. (c) 10X Genomics Library construction: a total of 240.56 Gb clean data were obtained, which covered the genome ~112.92×. Sequencing was performed in the Novogene Corporation.

### Hi‐C technology‐assisted genome assembly

Hi‐C technology spatially connected DNA sequences based on interactions between distantly located DNA fragments at physical locations. In that, the interaction probability is higher within the chromosome than between chromosomes and decreases with increased distance on the same chromosome; this method permits sorting and orienting contigs or scaffolds along a chromosome. The specific operations mainly include three steps: (a) comparison with draft genome, (b) clustering and (c) sorting and orientation.

### Gene prediction

We used multiple gene prediction methods, including homologous prediction, *de novo* prediction and other evidence‐supported predictions. (a) *Homologous* *prediction* was mainly conducted by BLAST and GeneWise software (Birney et al., 2004; Camacho et al., 2009). (b) *De novo prediction* mainly used software Augustus, GlimmerHMM (Stanke and Morgenstern, 2005) and SNAP (Korf, 2004). (c) *Other* *evidence‐supported predictions* mainly used EST or cDNA data from homologous species to predict gene structure by BLAT (Kent, 2002). Then, we integrated the above results into one nonredundant and more complete gene set using the IntegrationModeler software (Haas et al., 2008). Finally, we integrated the above results and our transcriptome data using PASA (Haas et al., 2003).

### Genome annotation

We annotated the genome with three features. (a) *Repeated sequence annotation* used two methods, homologous sequence alignment and *de novo* prediction. The former was based on repeat sequence database*,* using RepeatMasker and RepeatProteinMask software to identify repeat sequences (Bao et al., 2015; Tarailo‐Graovac and Chen, 2009). The *de novo* prediction firstly constructed the repeat sequence database by LTR_FINDER (Xu and Wang, 2007), Piler (Edgar and Myers, 2005), RepeatScout (Price et al., 2005) and RepeatModeler, then the repeated sequences were predicted by Repeatmasker. TRF software was used to detect tandem repeats (Benson, 1999). (b) *Gene annotation* involved comparing with known protein databases Swiss‐Prot, TrEMBL, KEGG and InterPro. (c) *Noncoding RNA annotation* used tRNAscan‐SE software to identify tRNA (Chan and Lowe, 2019). The rRNA was predicted by BLAST program. BLAST program was used to predict miRNAs and snRNAs (Nawrocki and Eddy, 2013).

### Gene family’s identification, amplification and contraction analysis

We used OrthoMCL to conduct gene family identification (Fischer et al., 2011) as follows: (a) filter gene set of each species. Only the longest transcript was retained when a gene had multiple alternative splicing transcripts, excluding genes that encode proteins less than 50 amino acids. (b) Obtain similarity relationships between protein sequences of all species by BLASTP with *e*‐value <1e‐5. (c) Compare and cluster results using 1.5 expansion co‐efficient, obtaining single‐copy and multicopy gene families. Gene family amplification and contraction analysis were performed using CAFE software (De Bie et al., 2006).

### Phylogenetic analysis and divergence time estimation

Multiple sequence alignments on all single‐copy genes were performed and then combined to construct a phylogenetic tree called a super alignment matrix. Here, we performed the construction of 13 species phylogenetic trees (ML TREE) by a maximum likelihood method using RAxML software (Stamatakis, 2014). This study used 519 single‐copy gene families to estimate divergence time using MCMCTree in the PAML software (Yang, 2007). The time correction points were as follows: *C. sativum* and *M.* *truncatula* (107–125 Mya), *B. rapa* and *P. trichocarpa* (107.0–109.0 Mya), *B. rapa* and *M. truncatula* (107–109 Mya), *O.* *sativa* and *N. nucifera* (140–200 Mya), and *B.* *rapa* and *A. thaliana* (20.4–30.9 Mya). The time correction points were from TimeTree website (Kumar et al., 2017). The operating parameters of MCMCTree were burn‐in = 5 000 000, sample number = 1 000 000 and sample frequency = 50.

### Transcriptome sequencing and analyses

Samples of coriander and carrot were collected from three different growth stages, including 30, 60 and 90 days after sowing. In addition, four tissues (root, stem, leaf and flower) were also used for RNA‐Seq analyses. Each sample included three biological replicates. The RNA was isolated from the tissues using a kit (Tiangen, Beijing, China) according to the manufacturer’s instructions. The main steps were as follows: (a) total RNA sample detection; (b) library construction; (c) library inspection; and (d) sequencing and bioinformatics.

Filtered reads were aligned to the genome assembly using HISAT (Kim et al., 2015). Alternative splicing was identified using the rMATS (Shen et al., 2014). The novel transcripts predicted by Cufflinks and FPKM (expected number of fragments per kilobase of transcript sequence per million base pairs sequenced) were used for estimating gene expression levels (Trapnell et al., 2010). We adopted HTSeq software to analyse the gene expression level (Anders et al., 2015) and DESeq software to conduct DEGs analyses with padj < 0.05 (Anders and Huber, 2010). The GO enrichment analysis was conducted using GOseq (Young et al., 2010), and the KOBAS was used for KEGG pathway enrichment analysis (Xie et al., 2011).

### Metabolomics analyses

We selected the leaf of coriander from three different growth stages, including 30, 60 and 90 days after sowing. At the same time, the leaf at these three stages was picked from the same sample for the transcriptome studies described above. The LC‐MS was used for metabolomics analysis, and each sample was set eight replications. The procedures mainly included the sample collection, metabolite extraction and LC‐MS/MS detection. Based on the raw data detected by mass spectrometry, we first import the original file into the CD (Compound discoverer) software and then perform spectral processing and database search. The quality control of the data is carried out to ensure the accuracy and reliability of the results. Multivariate statistical analysis of metabolites, including principal component analysis (PCA) and partial least squares analysis (PLS‐DA), reveals the differences in the metabolic composition of different alignment groups. The relationship between metabolites and samples was revealed using hierarchical clustering analysis (HCA) and metabolite–metabolite correlation analysis.

### Inference of gene colinearity

Colinear genes were inferred using ColinearScan (Wang et al., 2006). BLASTP search was performed to find putative homologous genes within a genome or between genomes. When running ColinearScan, maximal gap length between genes in colinearity along a chromosome sequence was set to 50 genes according to previous reports (Wang et al., 2017a; Wang et al., 2017b; Wang et al., 2016a; Wang et al., 2016b; Wang et al., 2005; Wang et al., 2015). Since large gene families lead to difficulty to infer gene colinearity, families with > 30 genes were removed before running ColinearScan.

To see directly the homology within and between genomes, homologous gene dot plots were produced using MCScanX (Wang et al., 2012). Dot plots were used to facilitate identification of homologous blocks produced by different polyploidization events (Wang et al., 2017a). Synonymous nucleotide substitution rates (Ks) were estimated between homologous genes, and the Ks median of a colinear block was shown in the dot plots to help group blocks produced by different events.

### Construction of the event‐related colinear gene table

To construct the table with the grape genome as a reference, all grape genes were listed in the first column. Each grape gene may have two additional colinear genes in its genome due to hexaploidy, and two other columns in the table listed this information. For a grape gene, when there was a corresponding colinear gene in an expected location, a gene ID was filled in a cell of the corresponding column in the table. When it was missing, often due to gene loss or translocation in the genome, the cell contained a dot. For the coffee genome, without extra duplications, we assigned one column. For the carrot or coriander genome, each affected by two palaeo‐tetraploidizations, we assigned four columns. Therefore, the table had 39 columns, reflecting layers of tripled and then fourfold homology due to recursive polyploidies across the genomes.

### Ks calculation, distribution fitting and correction

Ks were estimated using the Nei–Gojobori approach implemented by BioPerl Statistical module (Nei and Gojobori, 1986). We adopted a kernel function analysis of Ks distribution of colinear homologs within or between different genomes according to our previous reports (Wang et al., 2018; Wang et al., 2019). Ks distribution was viewed as a mix of multiple normal distributions. Kernel smoothing density function **ksdensity** (width is generally set to 0.05) in MATLAB was used to estimate the probability density of each Ks list to obtain the density distribution curve. Then, Gaussian multipeak fitting of the curve was inferred using Gaussian approximation function **Gaussian** in the fitting toolbox **cftool**. We set *R* ^2^, a parameter to evaluate the goodness of fit, to be at least 95%, using the smallest number of normal distributions to represent Ks distribution, and the principal one was used to represent the corresponding evolutionary event.

We estimated the evolutionary rates of ECH‐produced duplicated genes, corrected according to our report (Wang et al., 2019). The maximum likelihood estimate *µ* from inferred Ks means of ECH‐produced duplicated genes was aligned to have the same value of those of grape. Supposing a grape duplicated gene pair to have Ks value is a random variable $X_{G}∼\left(\mu_{G},\sigma_{G}^{2}\right)$, and for a duplicated gene pair in another genome, the Ks to be set as $X_{i}∼\left(\mu_{i},\sigma_{i}^{2}\right)$. Based on the fact that carrot and coriander shared two additional polyploidizations after the split with lettuce, and the different evolutionary rates of these two polyploidizations, we need to re‐correct their evolutionary rates. Here, since coriander had the slower rate during both of the two additional polyploidizations, we re‐corrected the evolutionary rates experienced in carrot with coriander as the reference. The specific methods were showed in the supplementary note.

## Conflicts of interest statement

The authors declare no competing interests.

## Authors' contributions

X.W. and X.S. conceived the project and were responsible for the project initiation. X.W., X.S. and J.W. supervised and managed the project and research. X.S., N.L., W.C., F.N., X.L., J.H., Q.Y., C.L. and S.F. designed the experiments and analyses. Q.P., T.Y., X.K., W.Z., L.W., W.G., D.G., C.C., Y.Y. and J.W. performed data generation and analysis. X.W., X. S., J. W., K.G., Z.Z., J.Y., F.M., C.L., Y.L., C.W., H.G., J.Y., T.L., X.D., S.S., Y.X., J.Z., Y.H. and J.W. performed bioinformatic analyses. X. S., X. W. and J.W. organized and wrote the manuscript. Most authors read and revised the manuscript. X.S. and J.W. contributed equally. Correspondence and requests for materials should be addressed to X.W.

## Supporting information


**Figures S1–S22** Supplementary Figures S1–S22.

**Tables S1–S51** Supplementary Tables S1–S51.

**Supinfo** Supplementary Notes (including legends).

## Data Availability

The genome sequence and RNA‐Seq data sets reported in this paper have been deposited in the Genome Sequence Archive (Wang et al., 2017c) in BIG Data Center (Members, 2019), Beijing Institute of Genomics (BIG), Chinese Academy of Sciences, under accession numbers CRA001654, CRA001656, CRA001657 and CRA001658 that are publicly accessible at ://bigd.big.ac.cn/gsa. The assembled coriander genome and related data set can be downloaded from the CGDB (http://cgdb.bio2db.com). All materials and other data in this study are available upon reasonable request.
