# Supplementary material for: Deciphering the high‐quality genome sequence of coriander that causes controversial feelings
Source: Plant Biotechnol J. 2020 Feb 5;18(6):1444–56. doi: 10.1111/pbi.13310 (PMC7206992; doi:10.1111/pbi.13310)
Supplement: Supplementary file 1 — Figures S1–S22 Supplementary Figures S1–S22. [file PBI-18-1444-s001.pdf]

Supplementary Figures 1-22

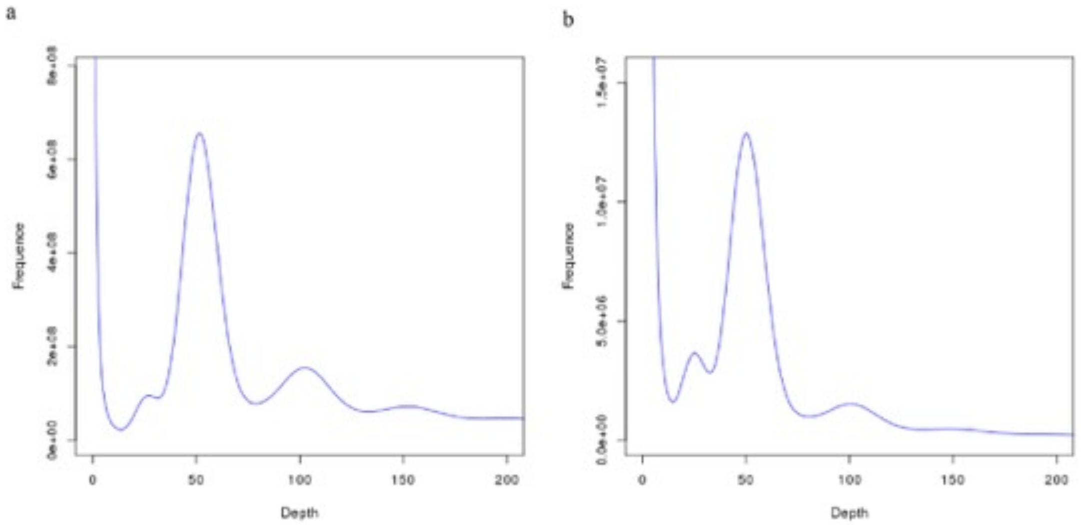

Supplementary Figure 1. Kmer distribution map. (a) Kmer=17 Depth and K-mer number frequency distribution. (b) Kmer=17 Depth and K-mer type frequency distribution.

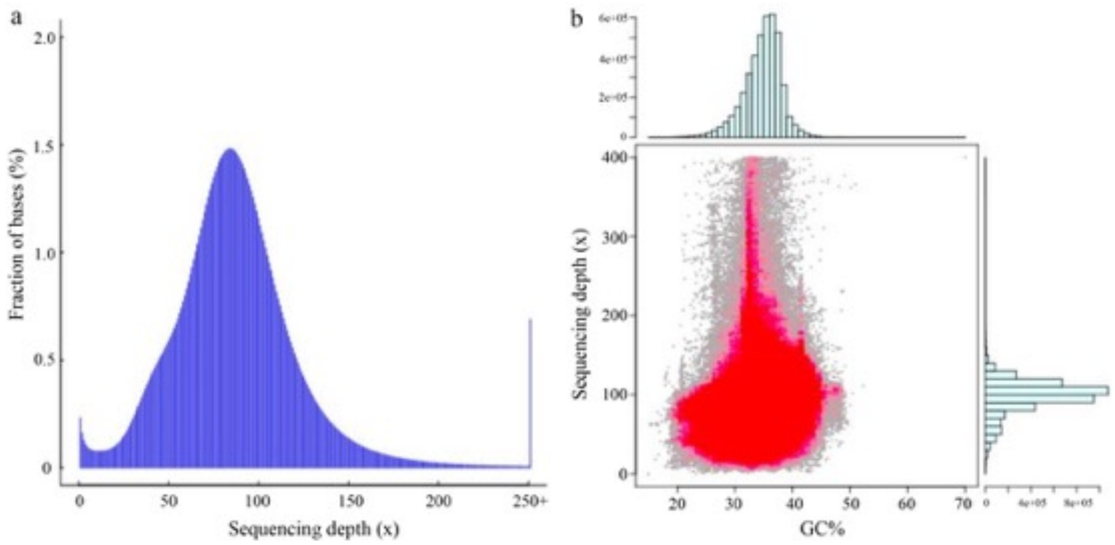

Supplementary Figure 2. The quality of genome assembly. (a) Sequencing depth profile

(horizontal axis: sequencing depth. Vertical axis: ratio of bases to genome (no N)). (b) GC content and depth profile. The main map is GC content distribution. Horizontal axis is GC content, and Vertical axis is Sequencing depth. On the histogram is GC content distribution map. Histogram right is sequencing depth profile.

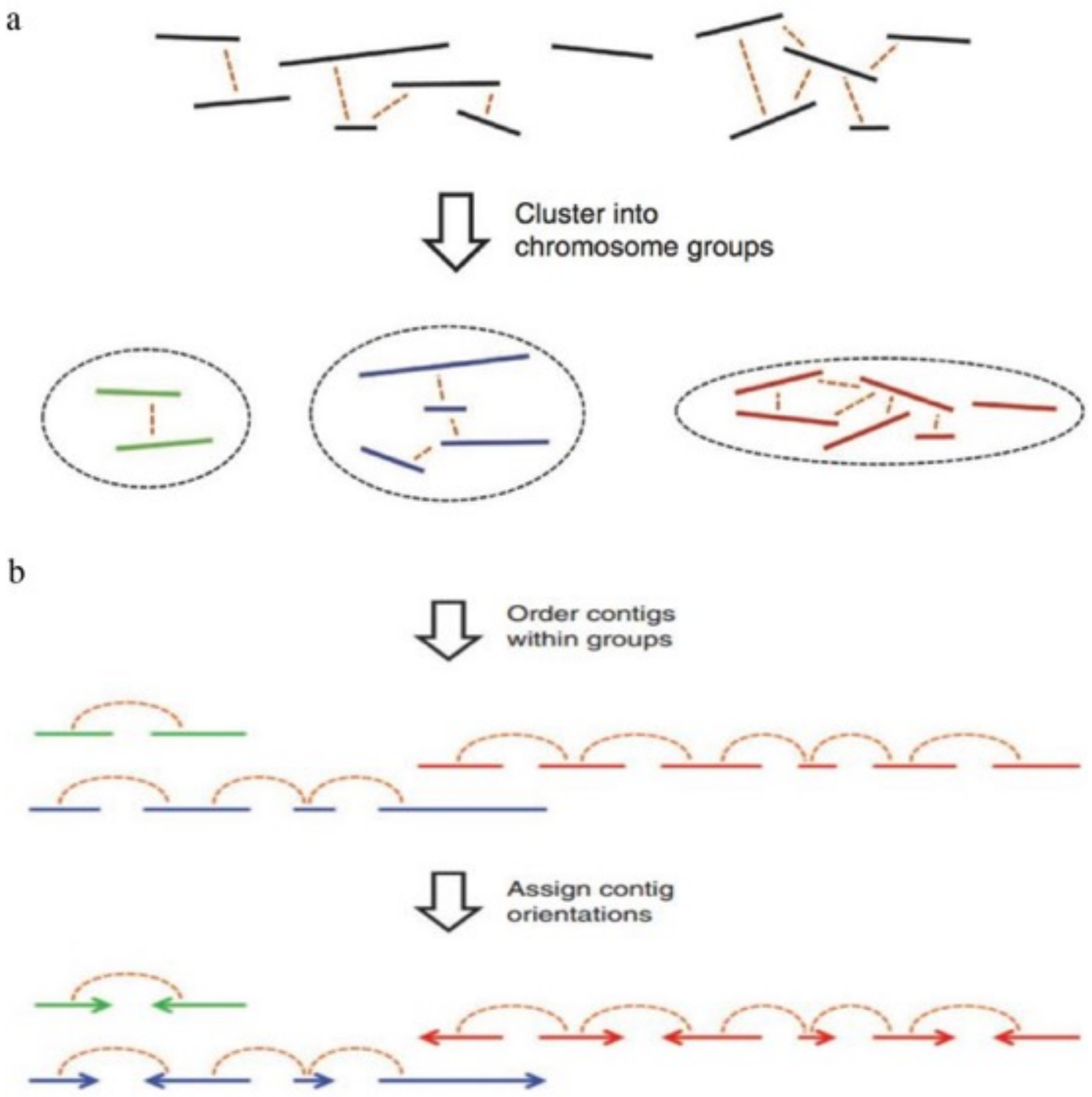

Supplementary Figure 3. The mainly Hi-C flowchart. (a) The theory of clustering. (b)The theory of sorting and orientation.

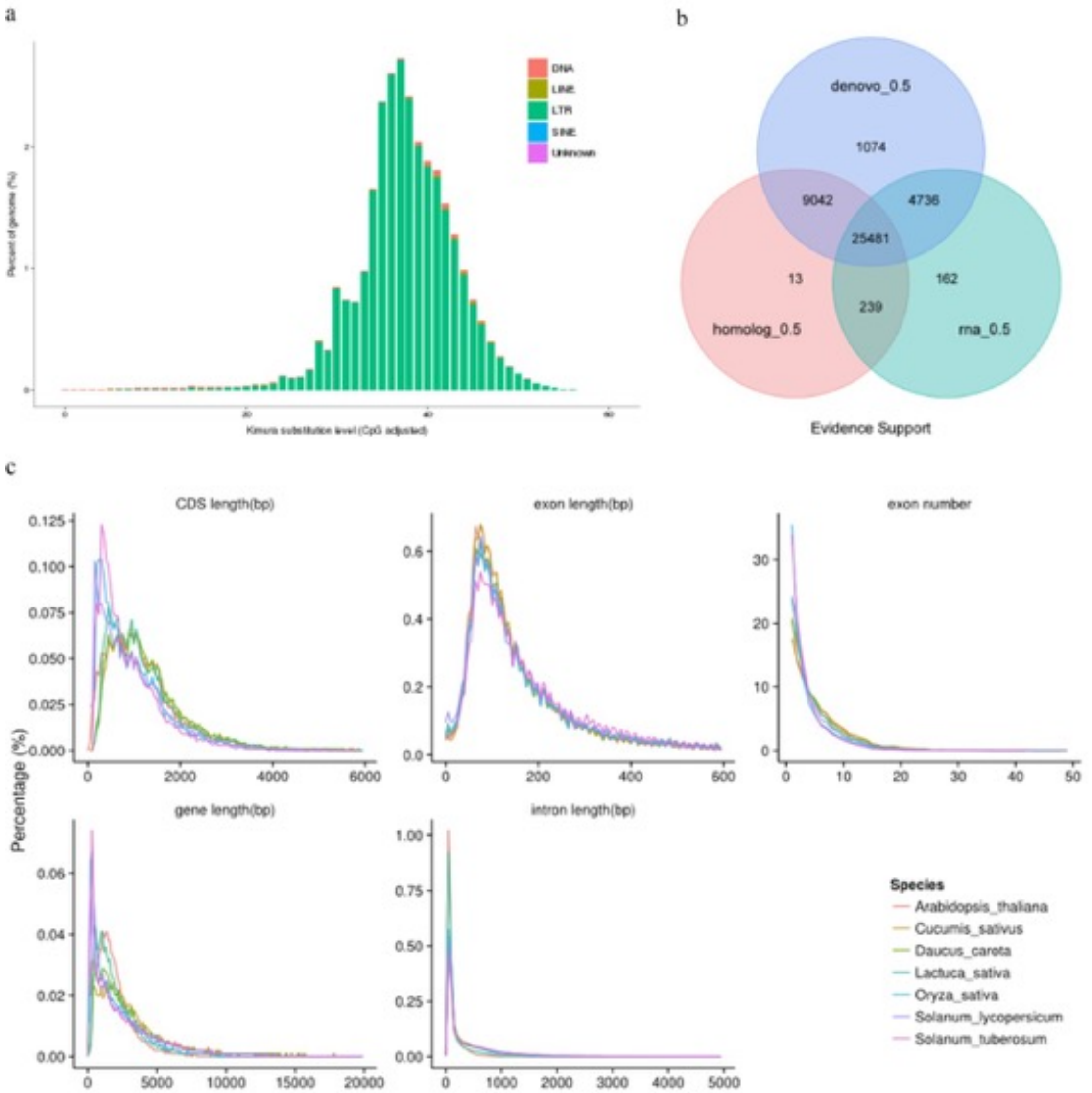

Supplementary Figure 4. (a) The frequency of the different type repeat sequence. (b) Gene set evidence supports statistics. Note: De novo_0.5, EVM integrates genes supported by *Denovo* prediction; Homolog_0.5, genes supported by homologous prediction when EVM integration; rna_0.5, genes supported by RNA-seq during EVM integration. The gene overlap is greater than 50% as a standard, and the number indicates the number of genes. (c) Comparison of gene components of *C. sativum* and other represent species.

a

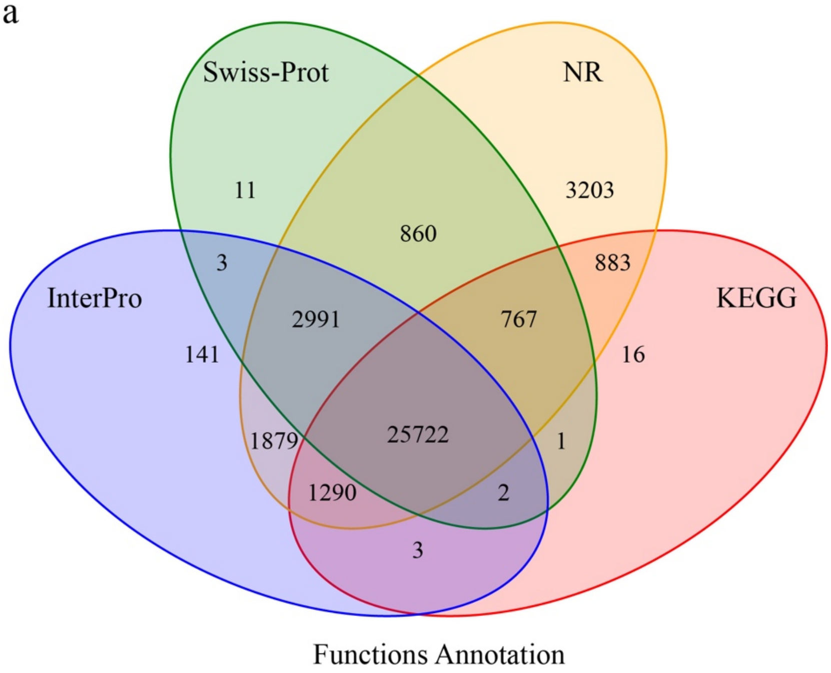

b

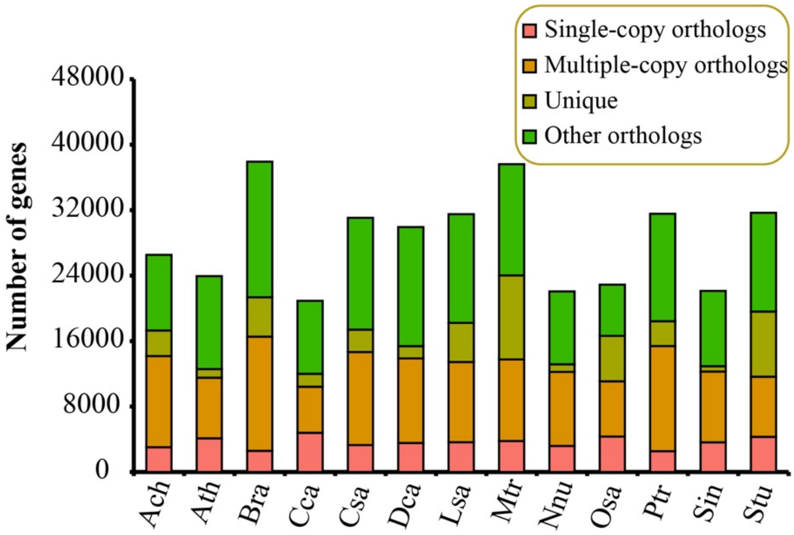

Supplementary Figure 5. (a) Statistical results of gene function annotations of *C. sativus*. (b) The distribution of gene numbers and family sizes in different species (Ach: *A. chinensis*; Ath: *A. thaliana*; Bra: *B. rapa*; Cca: *C. canephora*; Csa: *C. sativum*; Dca: *D. carota*; Lsa: *L. sativa*; Mtr: *M. truncatula*; Nnu: *N. nucifera*; Osa: *O. sativa*; Ptr: *P. trichocarpa*; Sin: *S. indicum*; Stu: *S. tuberosum*).

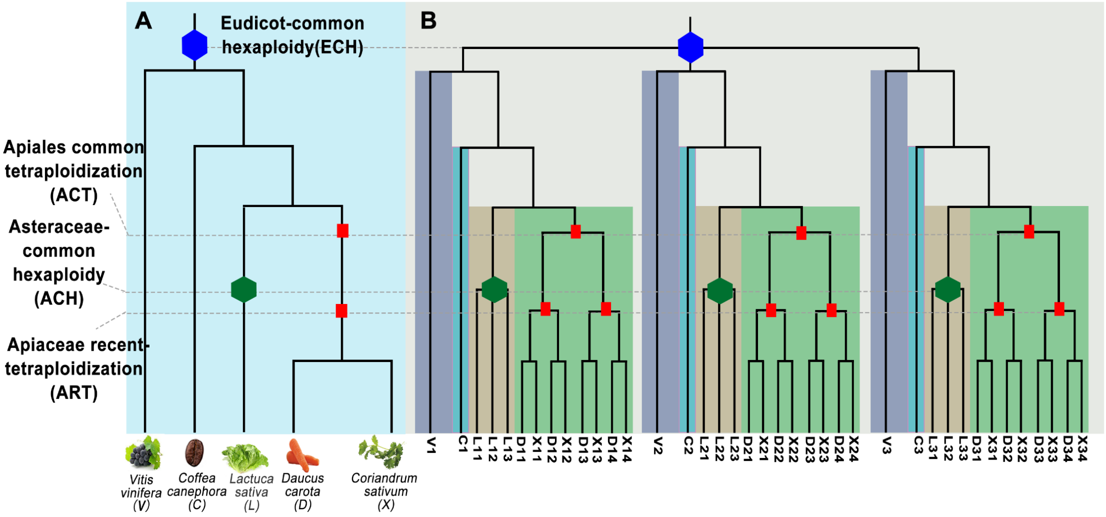

Supplementary Figure 6. Species and gene phylogenetic trees for the five genomes. (A) Phylogenetic tree of coriander (X), carrot (D), Lettuce (L), coffee (C), and grape (V): Eudicot-common hexaploidy (ECH) denoted by blue hexagon, the Asteraceae-common hexaploidy (ACH) denoted by green hexagon, and the two paleo-tetraploidization events denoted by red squares. (B) Gene phylogeny: three paralogous genes in the grape and coffee genomes are denoted by V1, V2, V3 and C1, C2, C3, produced by the ECH, and each has four orthologs and eight outparalogs in the coriander and carrot genome. For example, V1 has four orthologs D11, D12, D13, and D14, and eight outparalogs D21, D22, D23, D24, D31, D32, D33, and D34 in carrot. Each grape and coffee gene has three orthologs and six outparalogs in the lettuce genome. The species tree was produced based on our present analysis of homologous genes.

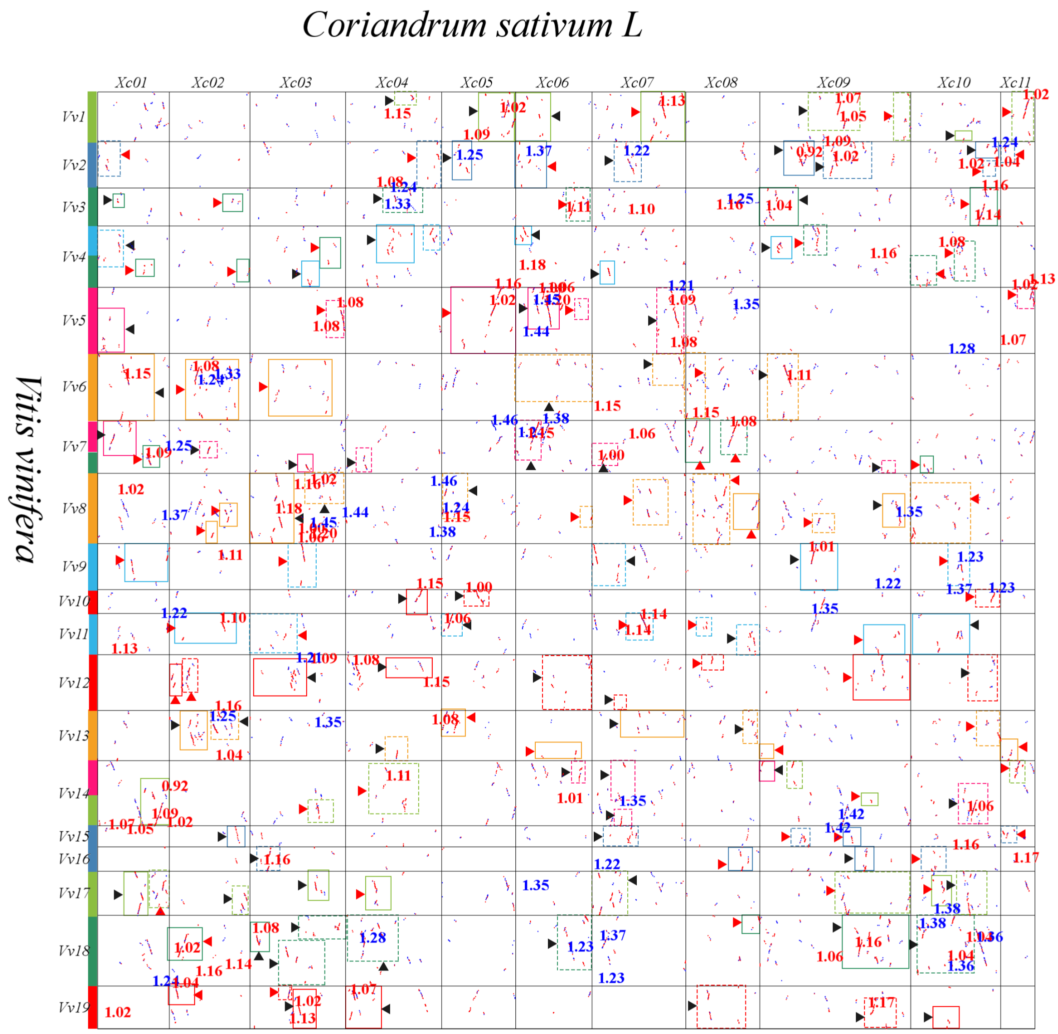

Supplementary Figure 7. Homologous dotplot between grape and coriander genomes. The best, secondary, and other matched homologous gene pairs output by Blast were dotplotted by using red, blue, and gray colors, respectively. Mean Ks of each inferred collinear block is shown besides. The Ks values <=0.85 are in red, and others in blue, often showing orthology and outparalogy, respectively. The 19 grape chromosomes are colored corresponding to the seven ancestral eudicot chromosomes, as described in the main text. Chromosome regions exhibiting genomic orthology were identified and are distinguished by solid and dashed rectangles corresponding to the ART and AAT events, respectively. Arrows of the same color indicate chromosomal segments on different cilantro chromosomes that correspond to the same grape chromosome, likely produced by chromosome breakages during evolution.

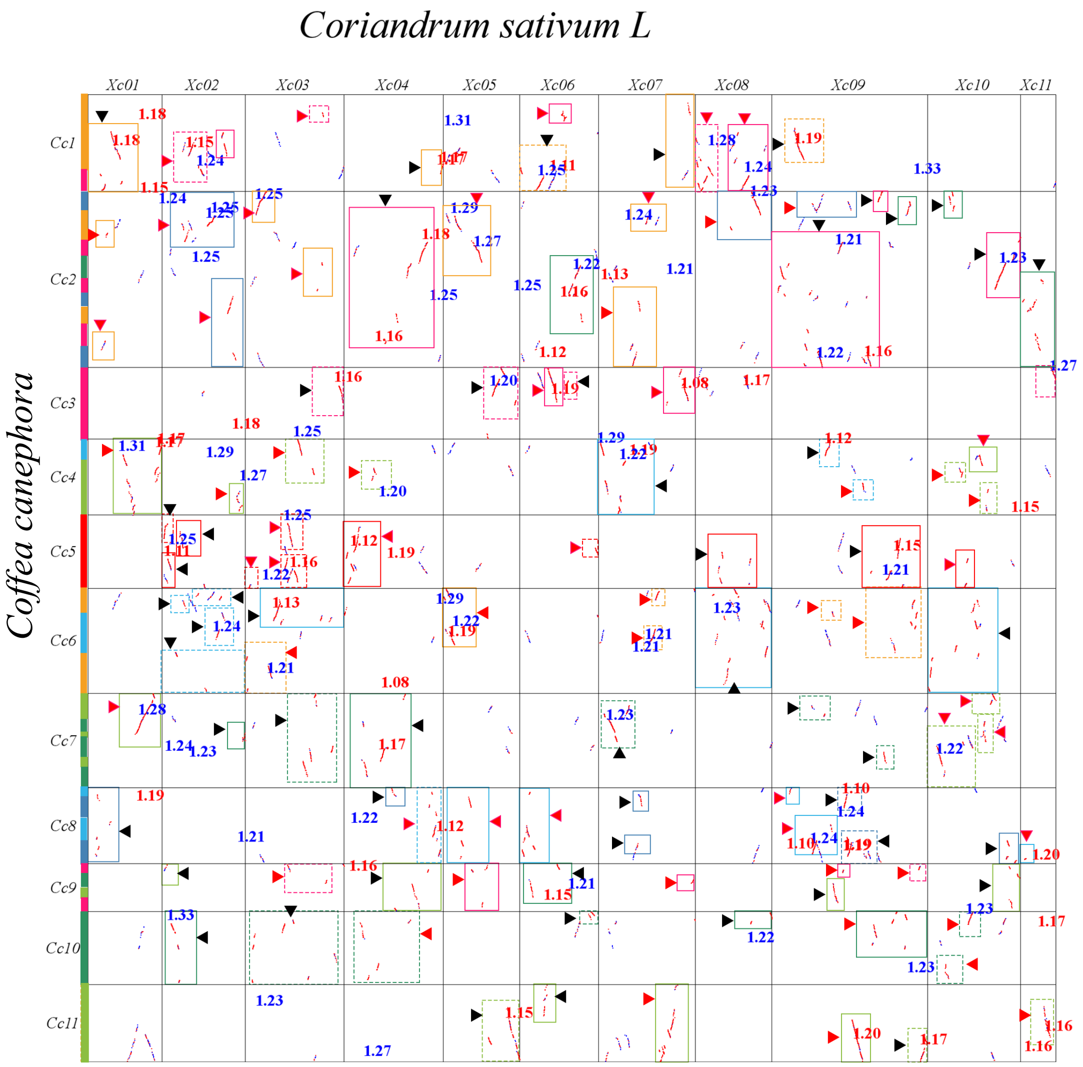

Supplementary Figure 8. Homologous dotplot between coffee and coriander genomes. The best, secondary, and other matched homologous gene pairs output by Blast were dotplotted by using red, blue, and gray colors, respectively. Mean Ks of each inferred collinear block is shown besides. The Ks values <=0.85 are in red, and others in blue, often showing orthology and outparalogy, respectively. The 11 cacao chromosomes are colored corresponding to the seven ancestral eudicot chromosomes, as described in the main text. Chromosome regions exhibiting genomic orthology were identified and are distinguished by solid and dashed rectangles corresponding to the ART and AAT events, respectively. Arrows of the same color indicate chromosomal segments on different cilantro chromosomes that correspond to the same coffee chromosome, likely produced by chromosome breakages during evolution.

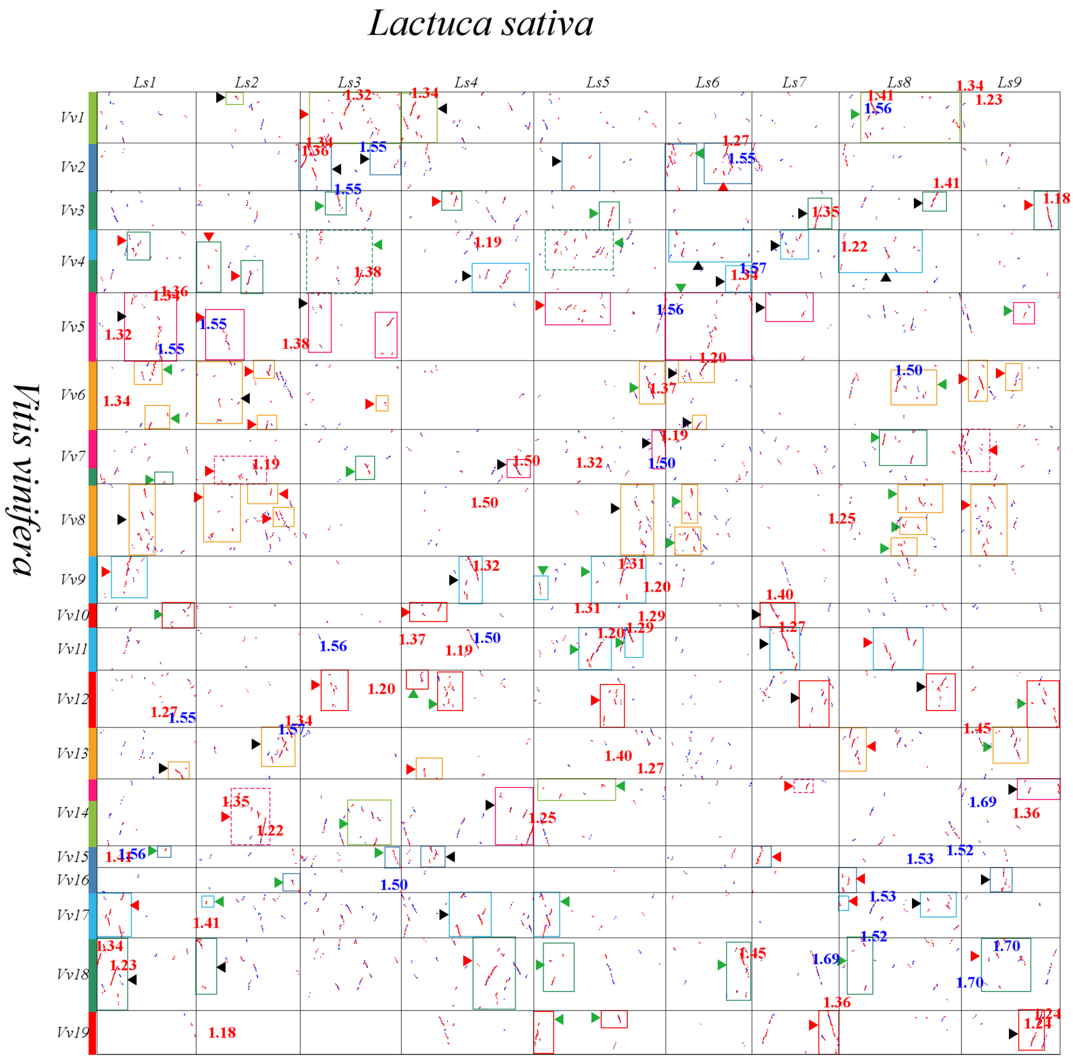

Supplementary Figure 9. Homologous dotplot between grape and lettuce genomes. The best, secondary, and other matched homologous gene pairs output by Blast were dotplotted by using red, blue, and gray colors, respectively. Mean Ks of each inferred collinear block is shown besides. The Ks values <=0.85 are in red, and others in blue, often showing orthology and outparalogy, respectively. The 19 grape chromosomes are colored corresponding to the seven ancestral eudicot chromosomes, as described in the main text. Chromosome regions exhibiting genomic orthology were identified and are distinguished by solid and dashed rectangles corresponding to the ART and AAT events, respectively. Arrows of the same color indicate chromosomal segments on different lettuce chromosomes that correspond to the same grape chromosome, likely produced by chromosome breakages during evolution.

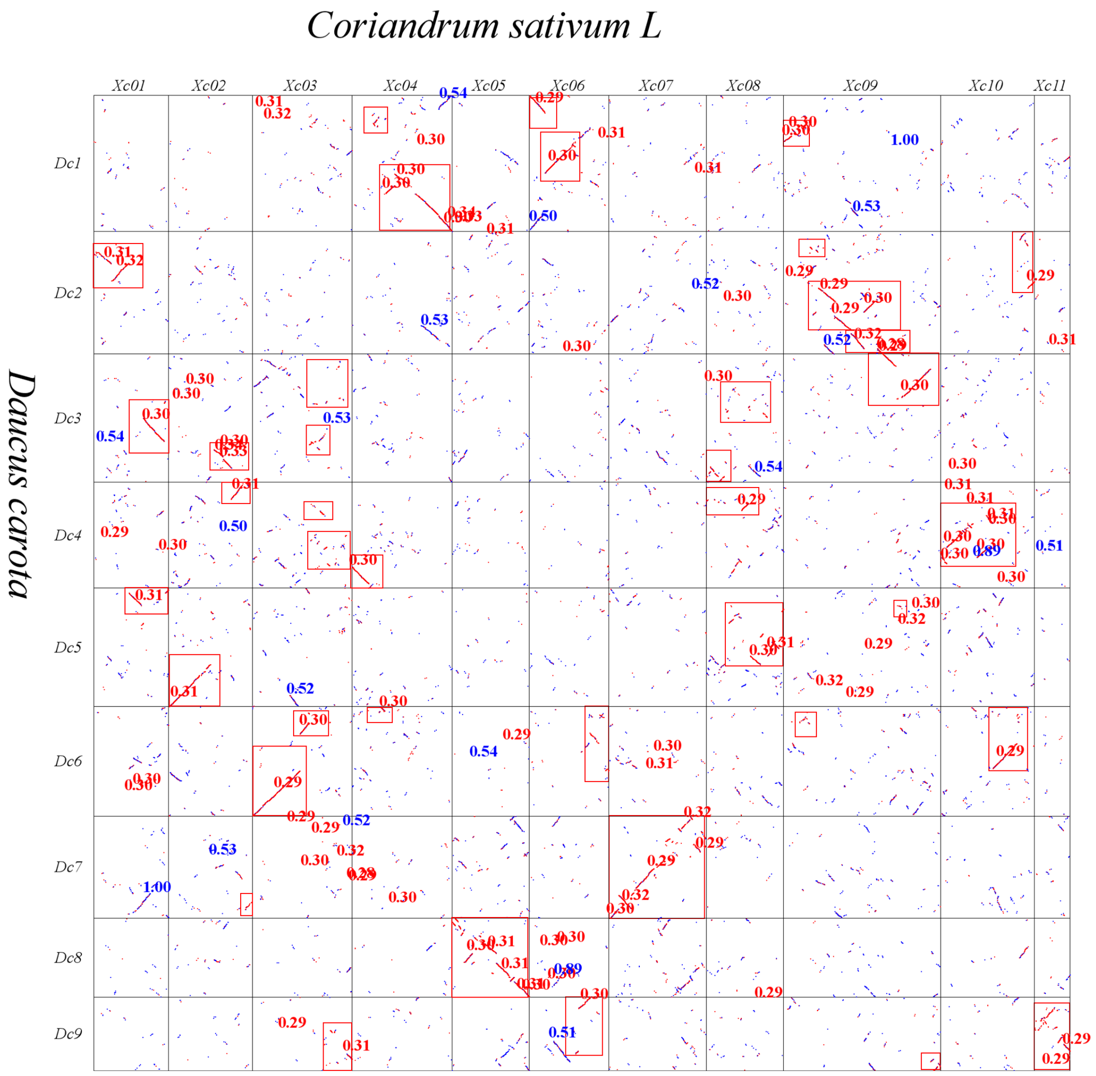

Supplementary Figure 10. Homologous dotplot between coriander and carrot genomes. The best, secondary, and other matched homologous gene pairs output by Blast were dotplotted by using red, blue, and gray colors, respectively. Mean Ks of each inferred collinear block is shown besides. The Ks values <=0.85 are in red, and others in blue, often showing orthology and outparalogy, respectively. Arrows of the same color indicate chromosomal segments on different Cilantro chromosomes that correspond to the same carrot chromosome, likely produced by chromosome breakages during evolution.

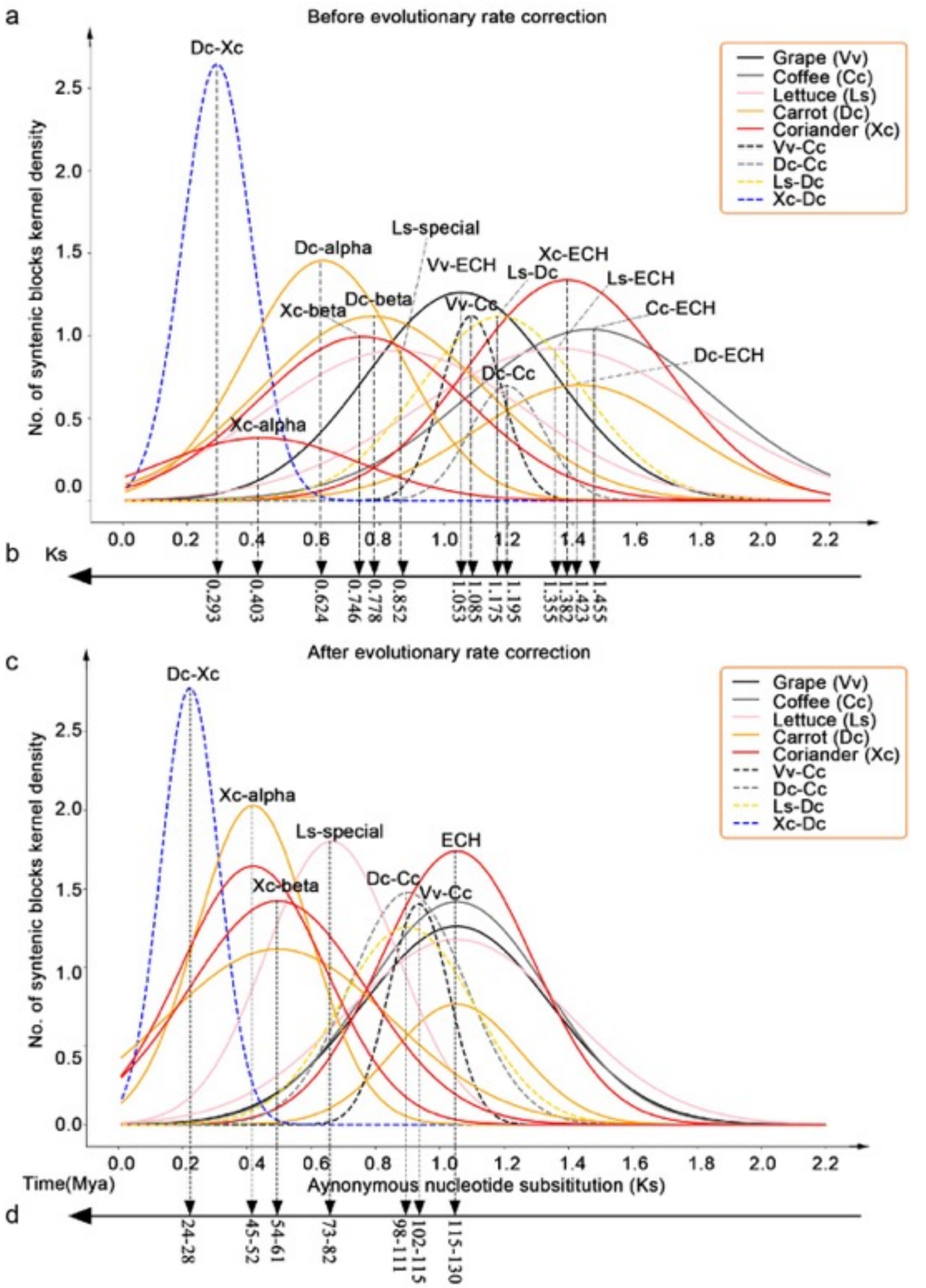

Supplementary Figure 11. The original and corrected synonymous nucleotide substitutions (Ks) among colinear genes. Continuous lines are used to show Ks distribution in a genome, and dashed lines are among genomes. a, Distributions fitted by using original Ks values; b, Infered means; c, Distributions fitted by using corrected Ks values; d, Inferred evolutionary dates.

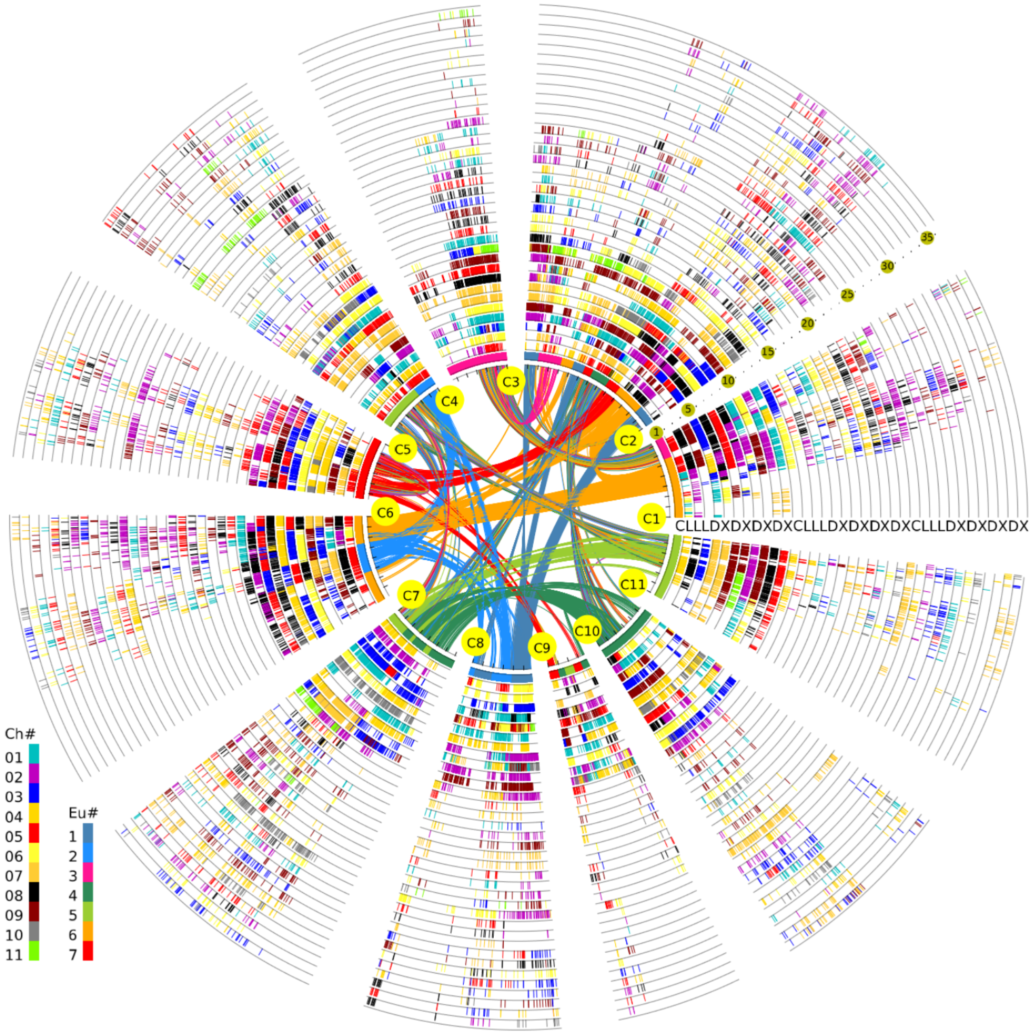

Supplementary Figure 12. Homologous alignments of coriander genomes with *coffee* as a reference. Genomic paralogy, orthology, and outparalogy information within and among genomes, with abbreviations as in Supplementary Figure 6, are displayed in 36 circles, each corresponding to an extant gene; The curved lines within the inner circle are formed by 11 coffee chromosomes color-coded to correspond to the 7 ancestral chromosomes before the ECH. The short lines forming the innermost coffee chromosome circles represent predicted genes, which have 2 sets of paralogous regions, forming another two circles. Each of the three sets of coffee paralogous chromosomal regions has 3 orthologous copies in lettuce, and 4 copies in coriander and carrot. The resulting 36 circles were marked according to species by a capital letter. Each circle has an underline colored as to its source plant corresponding to the color scheme in Supplementary Figure 6 and each circle is formed by short vertical lines that denote homologous genes, colored as to chromosome number in their respective source plant as shown in the inset color scheme.

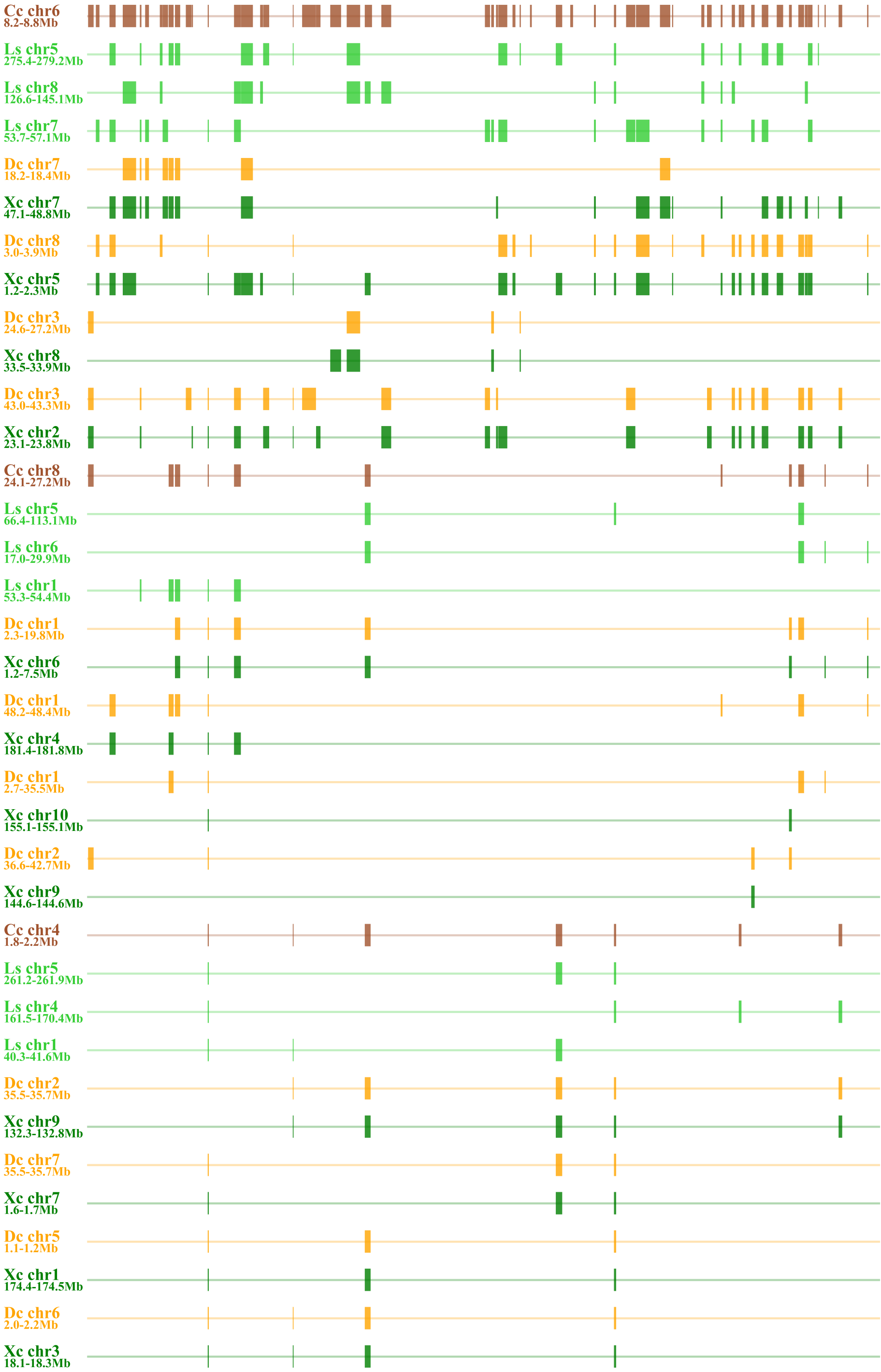

Supplementary Figure 13. Local alignment analyses. Using coffee chromosomes 6, 8 and 4, which were produced by the ECH as reference, we displayed the alignment of a region from 8.2 to 8.8 Mb on chromosome 6, 24.1 to 27.2 Mb on chromosome 8, 1.8 to 2.2 Mb on chromosome 4, along with its corresponding regions from all other genomes. Chromosome numbers and locations are shown after the names of plants. The gene is shown by a rectangle and its position corresponds to the position of the gene that is collinear on chromosomes 6, 8 and 4 of the coffee.

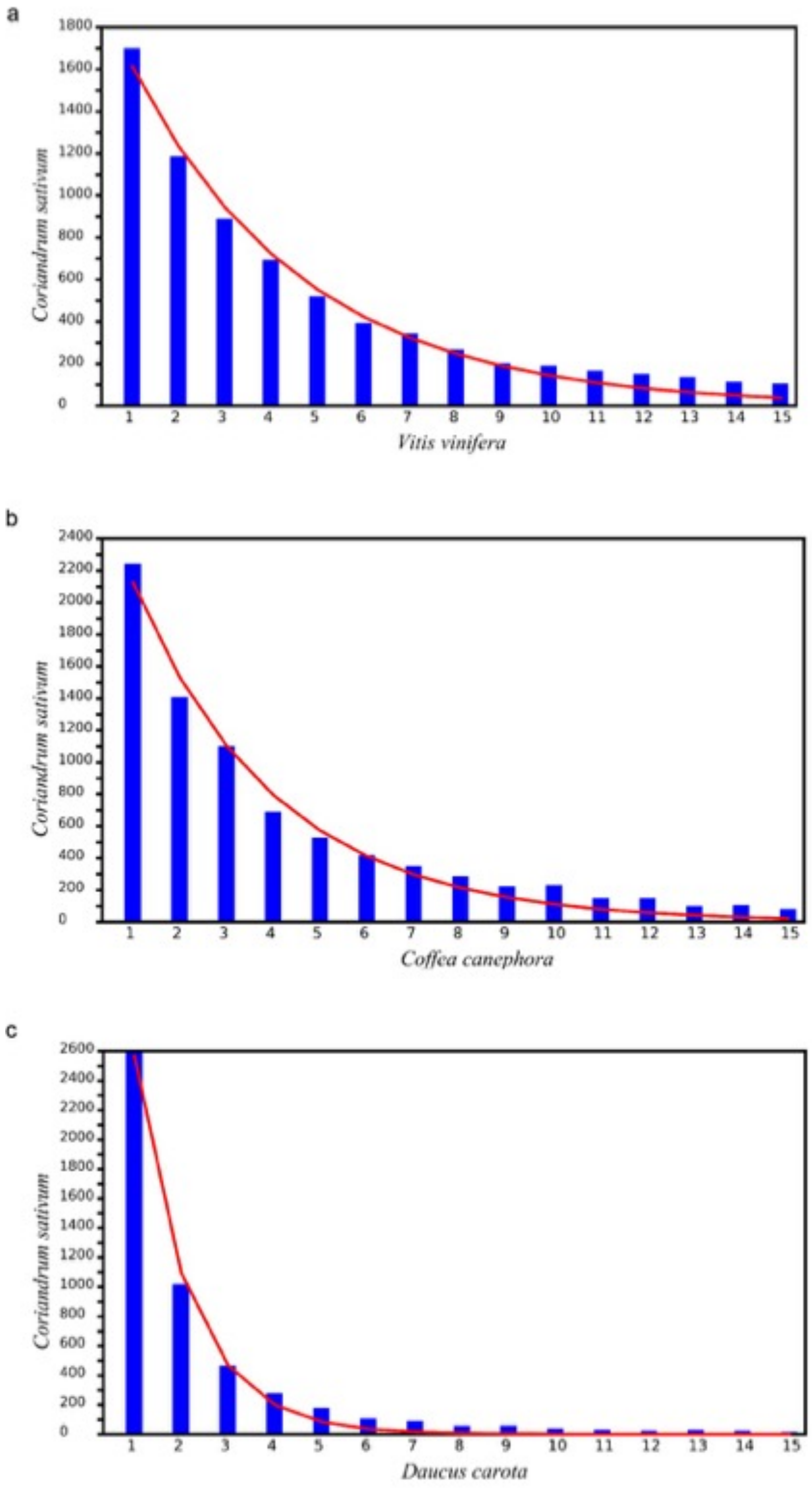

Supplementary Figure 14. Fitting a geometric distribution and gene loss rates in *C. sativum* as to the grape (a), coffee (b), and carrot (c), respectively.

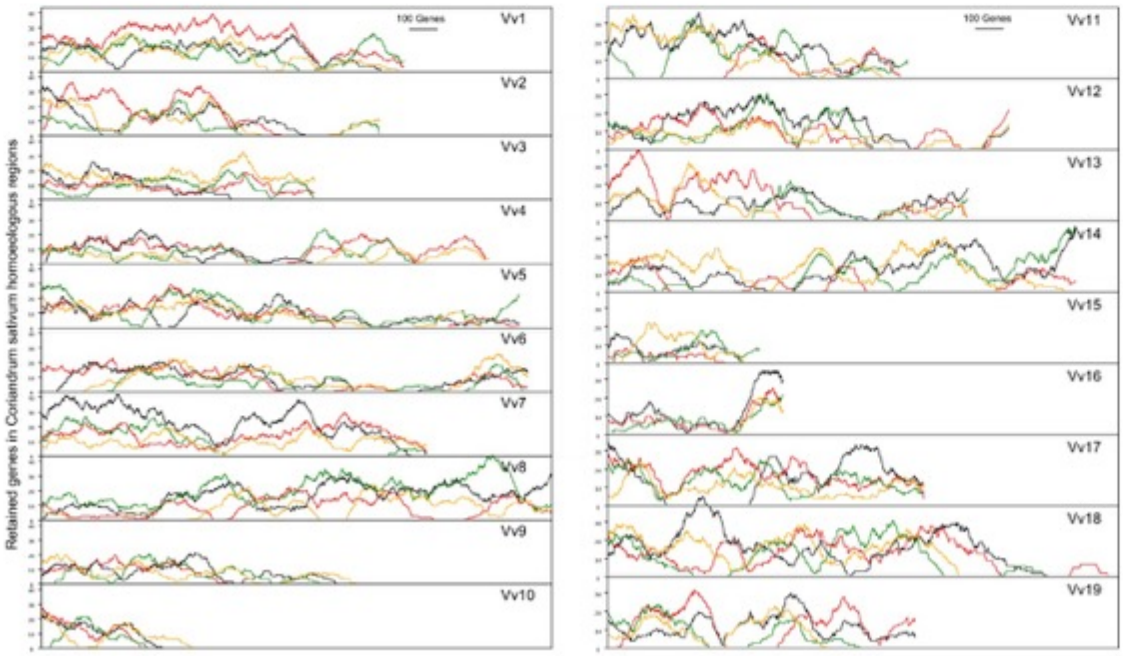

Supplementary Figure 15. The retaintion of duplicated genes reside in 4 subgenomes of *C. sativum* using the grape as reference.

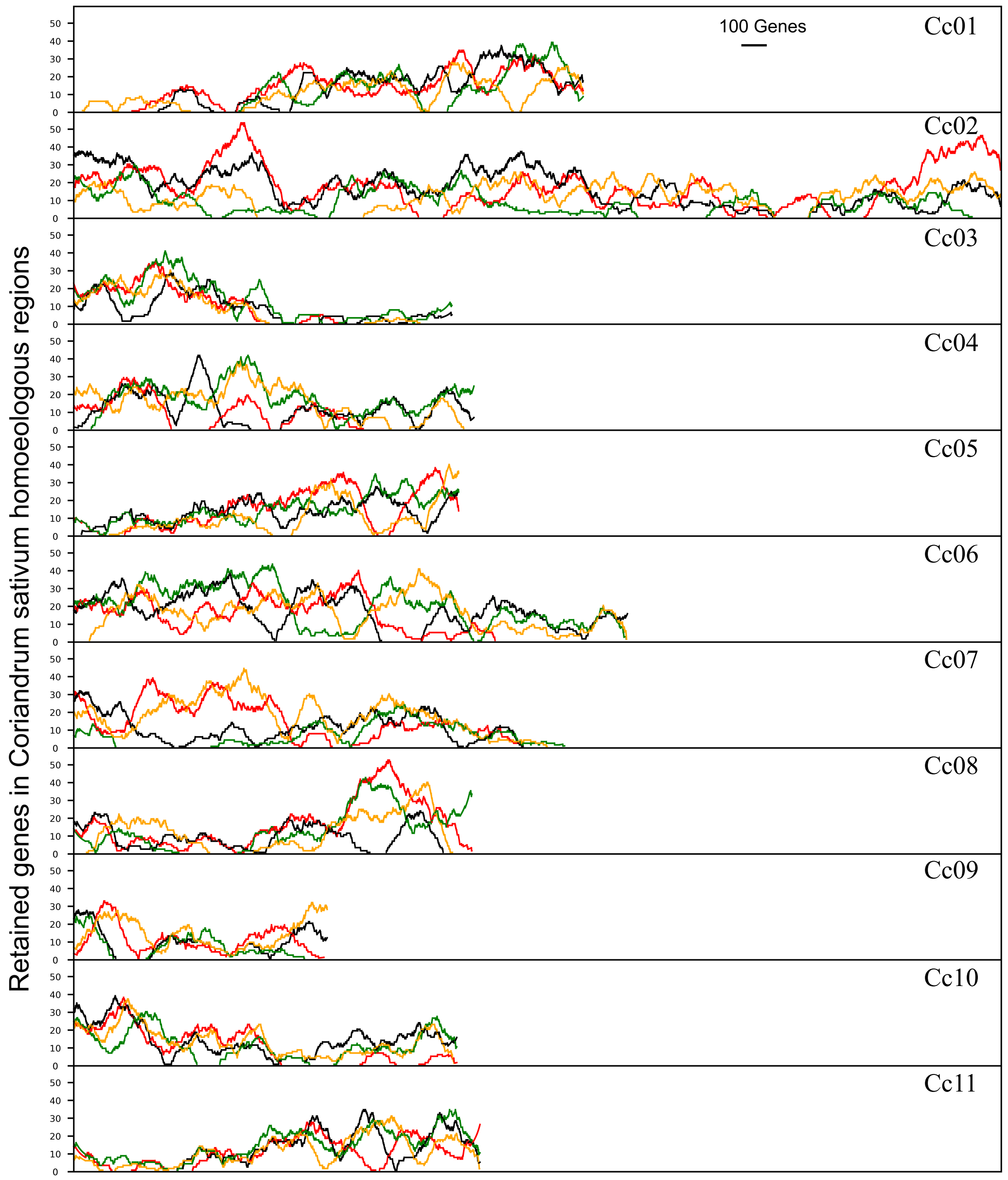

Supplementary Figure 16. The retaintion of duplicated genes reside in 4 subgenomes of *C. sativum* using the coffee as reference.

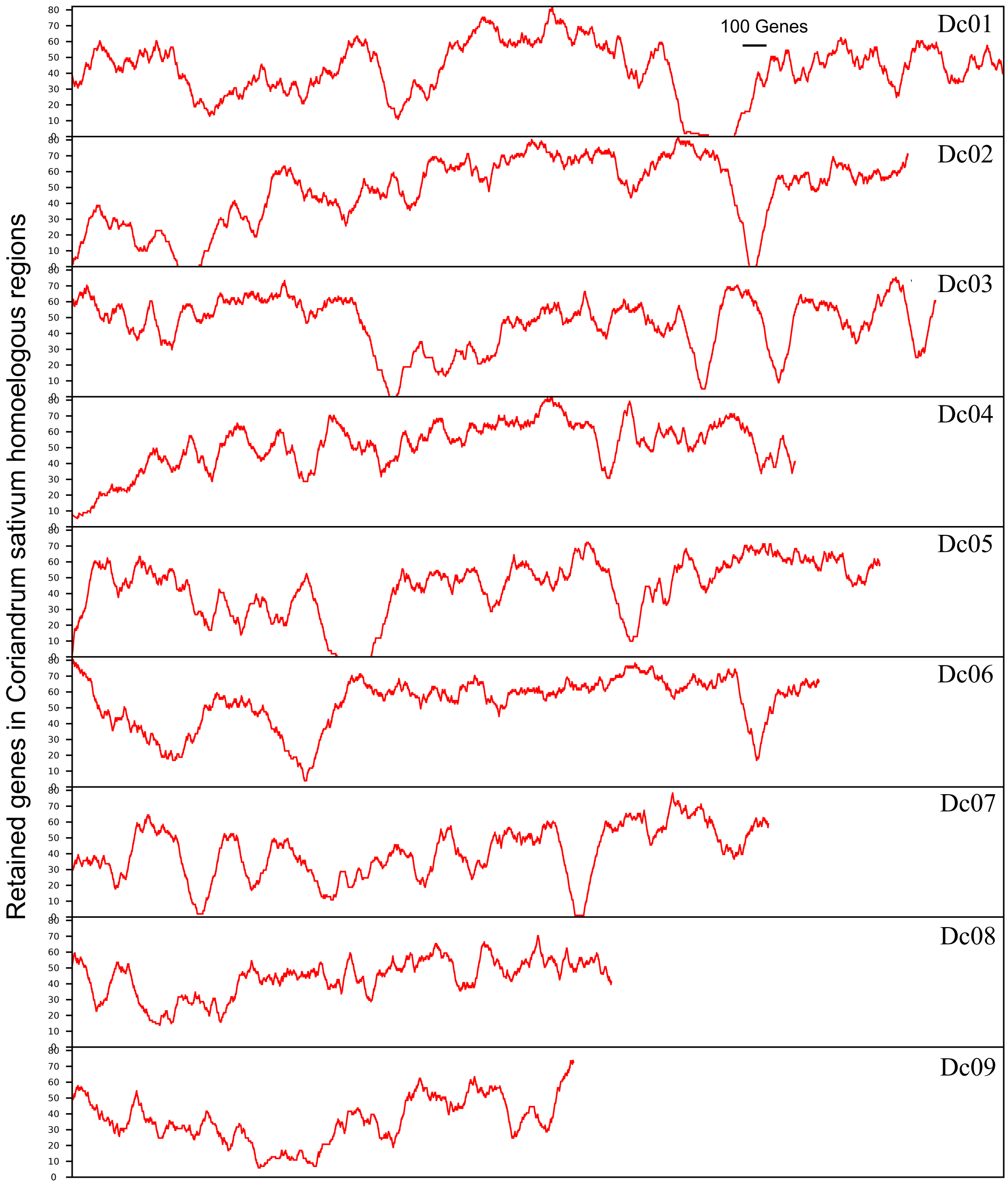

Supplementary Figure 17. The retaintion of duplicated genes reside in genomes of *C. sativum* using the carrot as reference.

expected topology

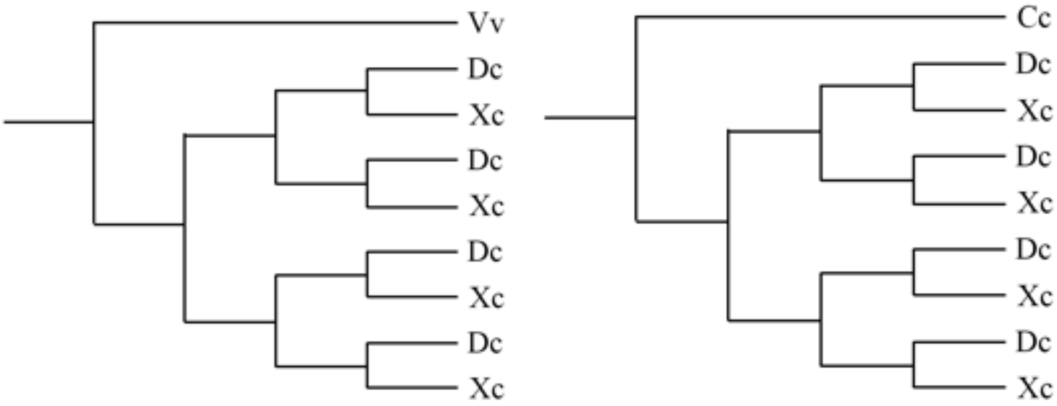

agree with expectations

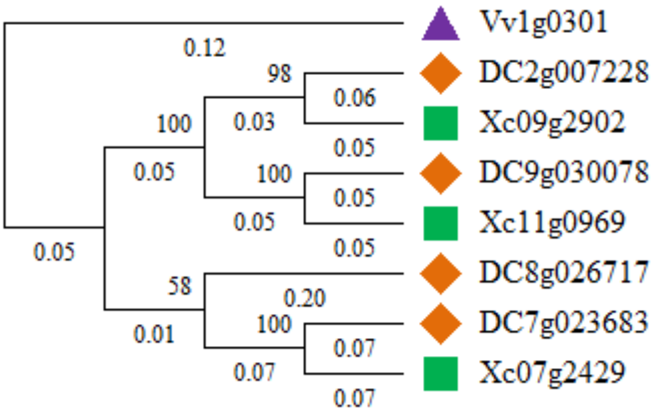

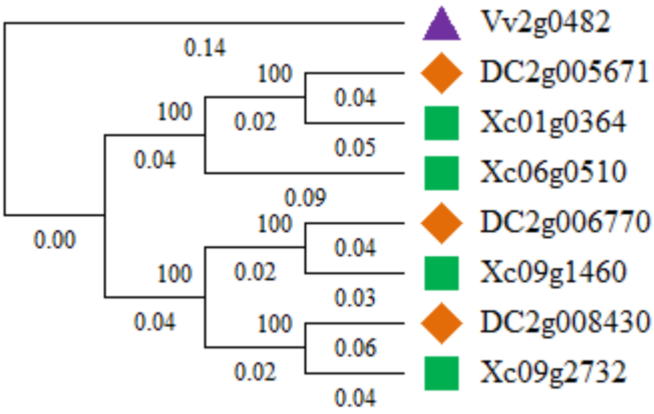

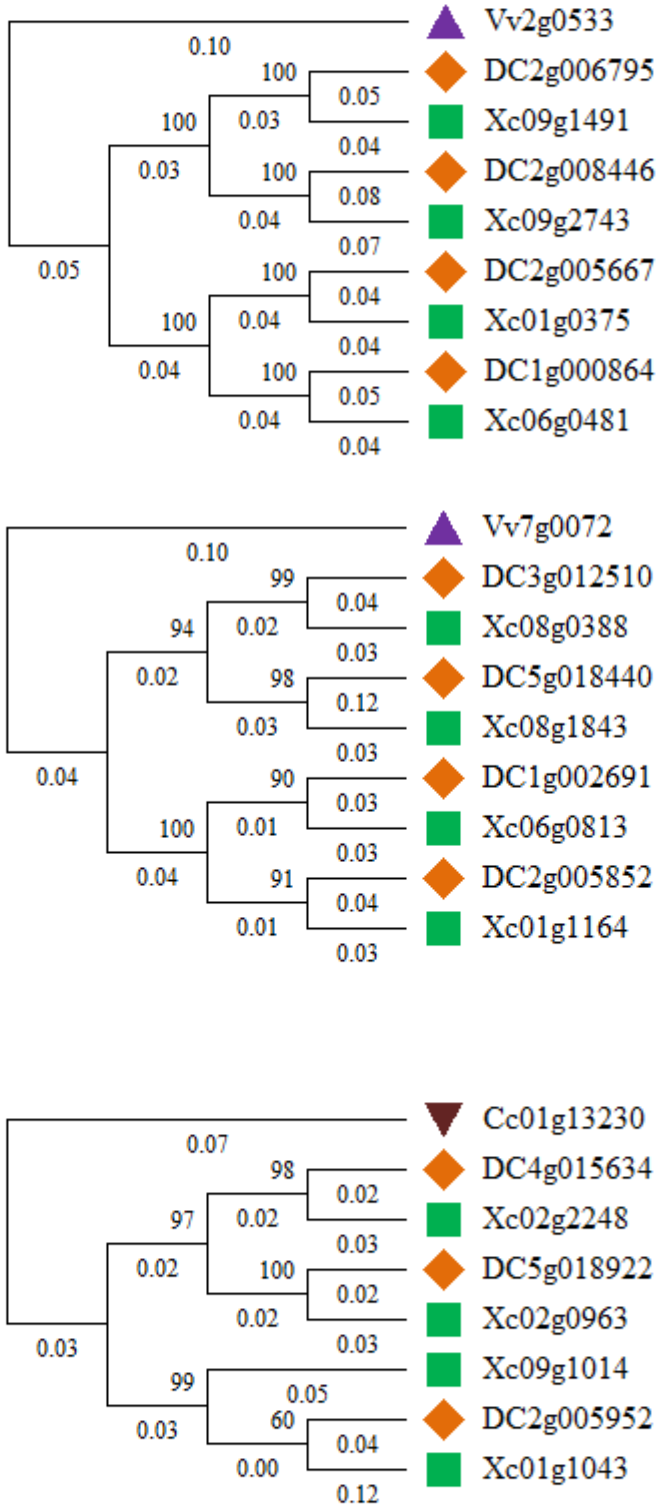
Vv2g0533
DC2g006795
Xc09g1491
DC2g008446
Xc09g2743
DC2g005667
Xc01g0375
DC1g000864
Xc06g0481
0.10
100
0.05
100
0.03
0.04
0.03
100
0.08
0.04
0.07
0.05
100
0.04
100
0.04
0.04
0.04
100
0.05
0.04
0.04
Vv7g0072
DC3g012510
Xc08g0388
DC5g018440
Xc08g1843
DC1g002691
Xc06g0813
DC2g005852
Xc01g1164
0.10
99
0.04
94
0.02
0.03
0.02
98
0.12
0.03
0.03
0.04
90
0.03
100
0.01
0.03
0.04
91
0.04
0.01
0.03
Cc01g13230
DC4g015634
Xc02g2248
DC5g018922
Xc02g0963
Xc09g1014
DC2g005952
Xc01g1043
0.07
98
0.02
97
0.02
0.03
0.02
100
0.02
0.02
0.03
0.03
99
0.05
60
0.03
0.04
0.00
0.12

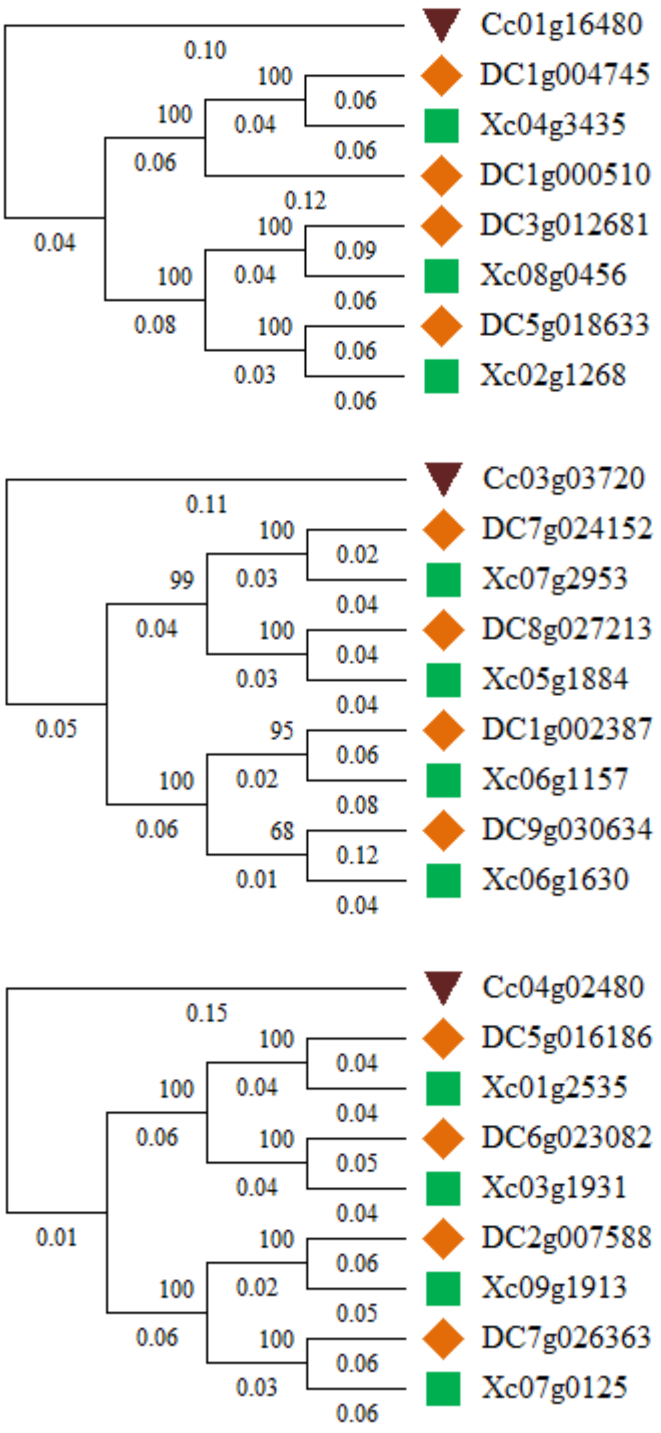

disagree with expectations

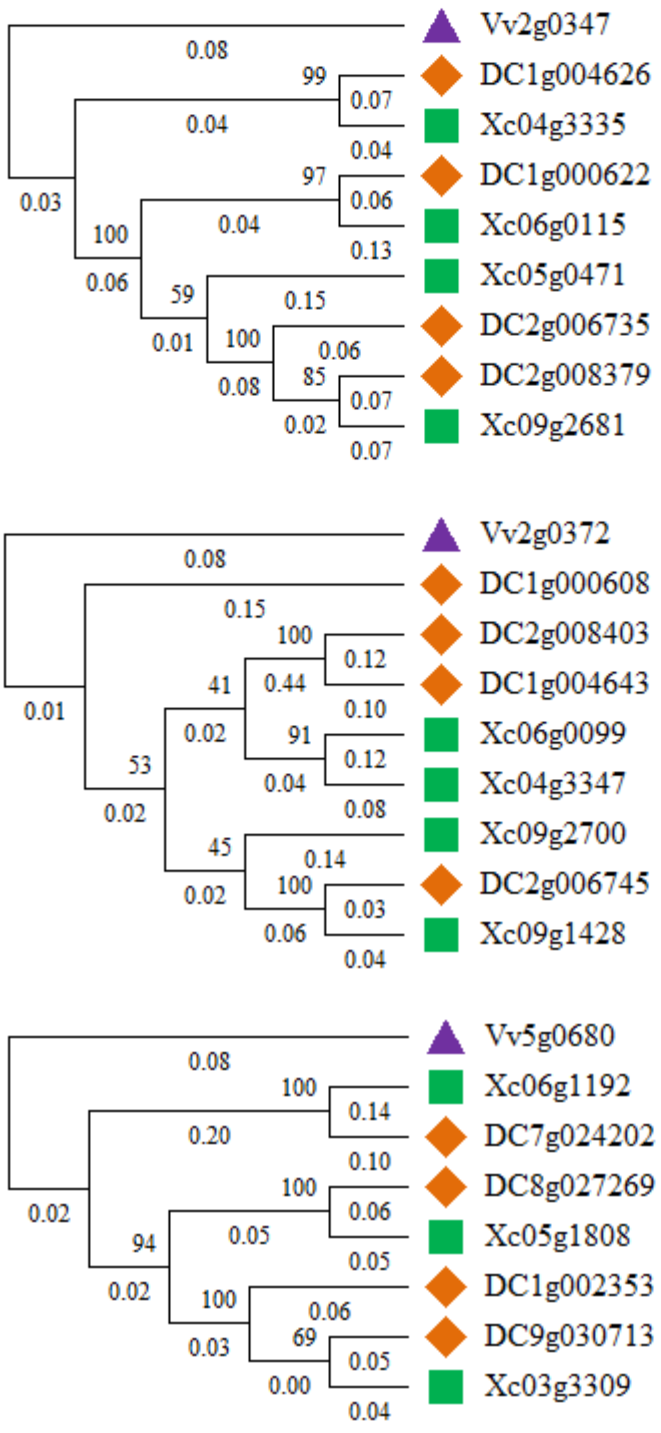
Vv2g0347
0.08
99
DC1g004626
0.07
Xc04g3335
0.04
0.04
97
DC1g000622
0.06
0.03
Xc06g0115
0.04
100
0.13
Xc05g0471
0.06
59
0.15
DC2g006735
0.01
100
0.06
85
DC2g008379
0.08
0.07
Xc09g2681
0.02
0.07
Vv2g0372
0.08
DC1g000608
0.15
100
DC2g008403
0.12
41
0.44
DC1g004643
0.01
0.10
0.02
91
Xc06g0099
0.12
53
Xc04g3347
0.04
0.02
0.08
Xc09g2700
45
0.14
100
DC2g006745
0.02
0.03
Xc09g1428
0.06
0.04
Vv5g0680
0.08
100
Xc06g1192
0.14
0.20
DC7g024202
0.10
100
DC8g027269
0.02
0.06
94
0.05
Xc05g1808
0.05
0.02
DC1g002353
100
0.06
69
DC9g030713
0.03
0.05
0.00
Xc03g3309
0.04

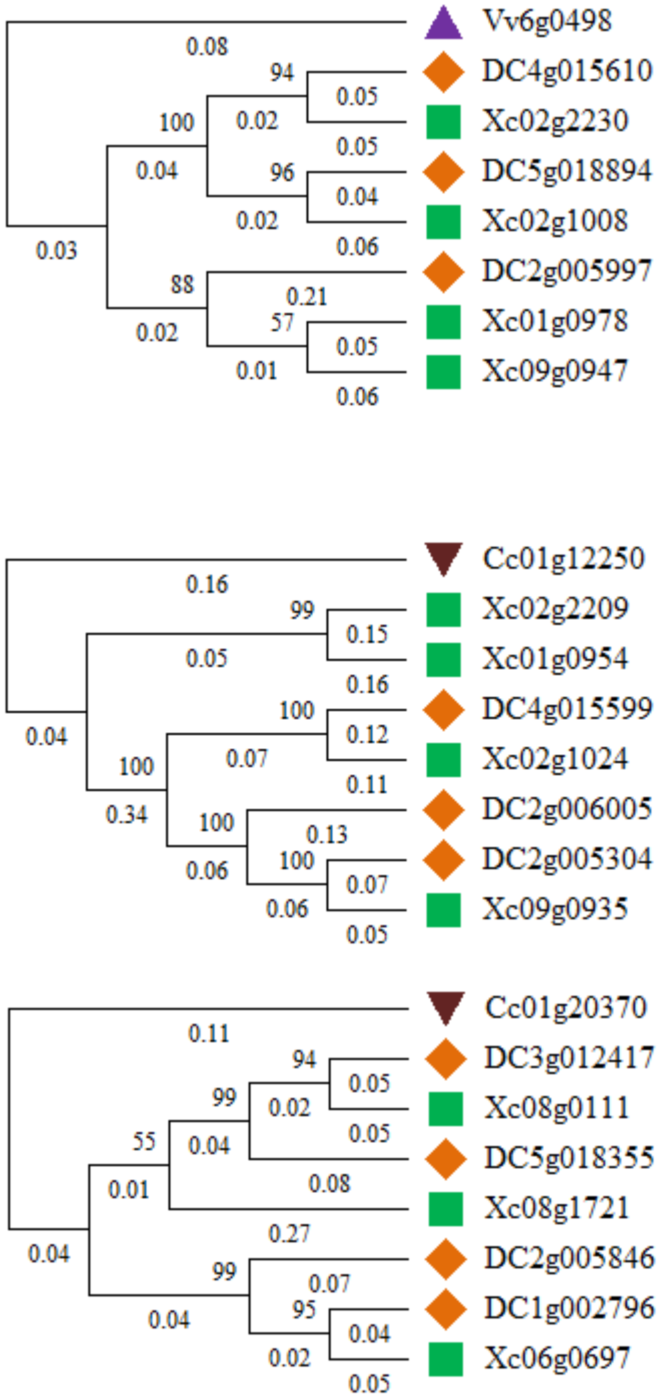
Vv6g0498
DC4g015610
Xc02g2230
DC5g018894
Xc02g1008
DC2g005997
Xc01g0978
Xc09g0947
0.08
94
0.05
100
0.02
0.05
0.04
96
0.04
0.02
0.06
0.03
88
0.21
57
0.02
0.05
0.01
0.06
Cc01g12250
Xc02g2209
Xc01g0954
DC4g015599
Xc02g1024
DC2g006005
DC2g005304
Xc09g0935
0.16
99
0.15
0.05
0.16
100
0.04
0.12
0.07
100
0.11
0.34
100
0.13
100
0.06
0.07
0.06
0.05
Cc01g20370
DC3g012417
Xc08g0111
DC5g018355
Xc08g1721
DC2g005846
DC1g002796
Xc06g0697
0.11
94
0.05
99
0.02
55
0.04
0.05
0.01
0.08
0.27
0.04
99
0.07
95
0.04
0.04
0.02
0.05

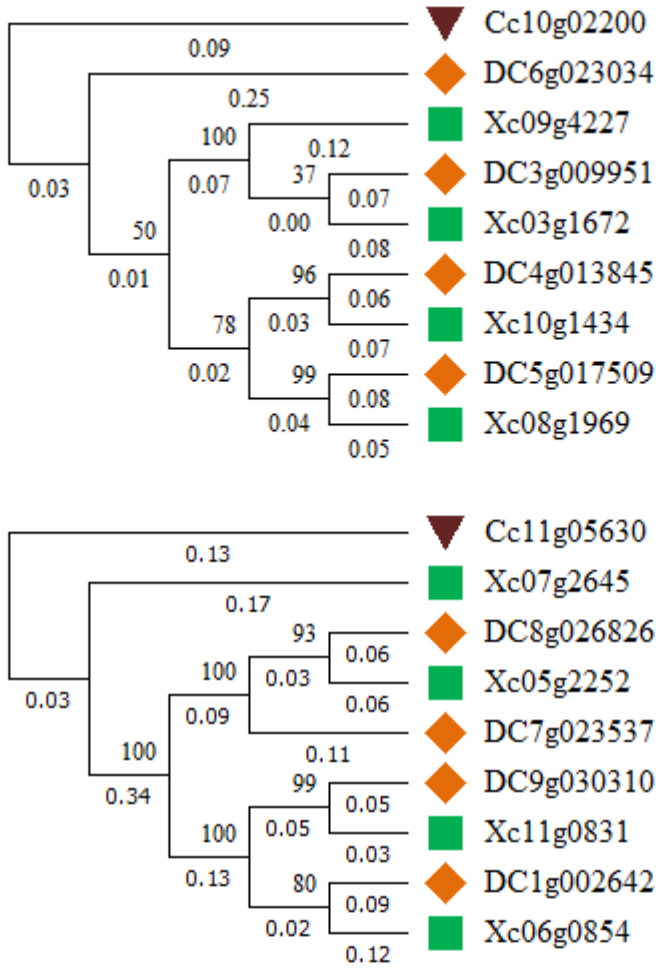

Supplementary Figure 18. The statistics of the expected topology, agree with expectations, and disagree with expectations of the evolutionary trees. The homologous gene evolutionary trees were constructed with one grape gene and one coffee gene as outgroups, and each containing at least two carrot genes and at least two coriander genes.

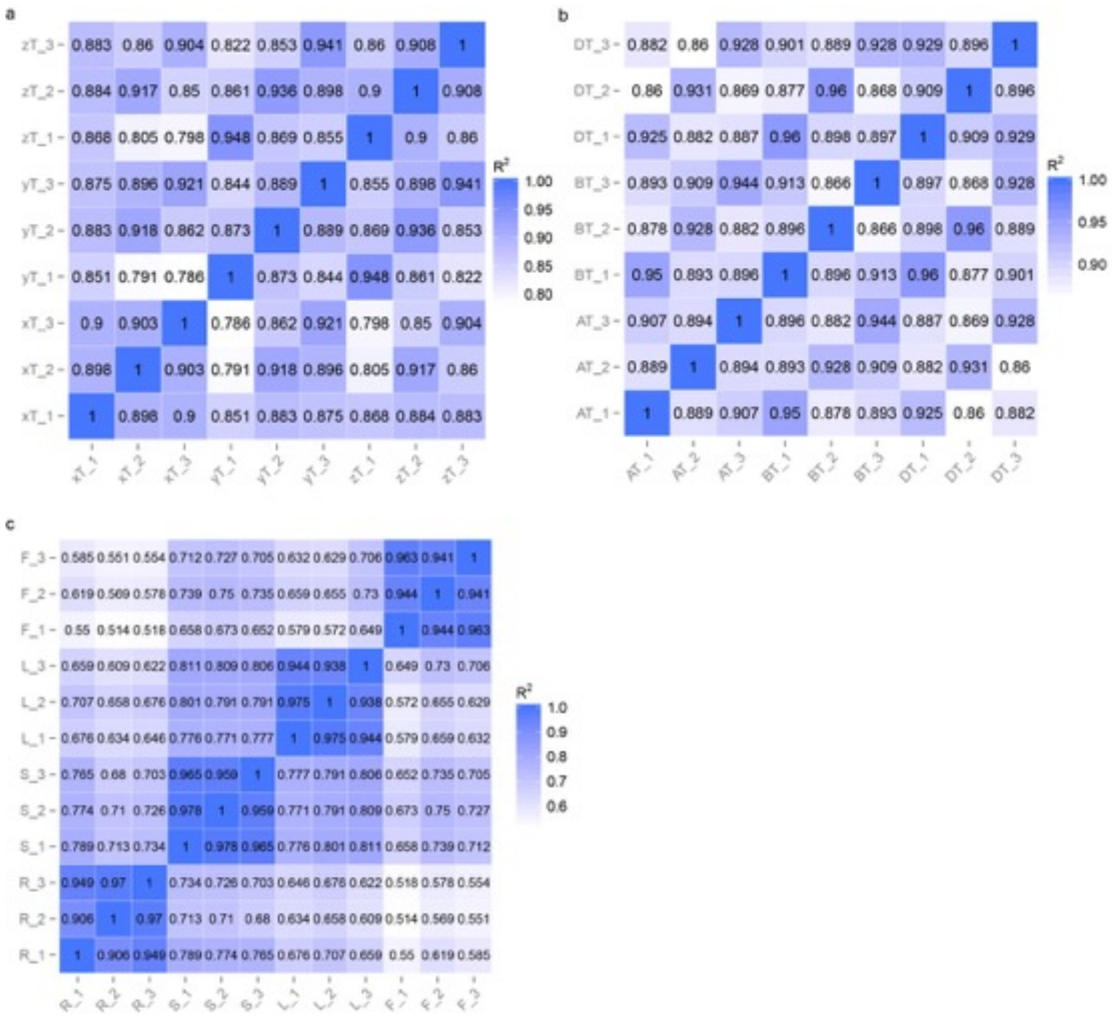

Supplementary Figure 19. The heat map of correlation coefficients were showed between any two of samples by RNA-Seq. (a) The RNA-Seq for 3 different growth stages (30d, 60d and 90d) of *C. sativum*. (b)The RNA-Seq for 3 different growth stages (30d, 60d and 90d) of *D. carota*. (c) The RNA-Seq for four tissues (root, stem, leaf, and flower) of *C. sativum*.

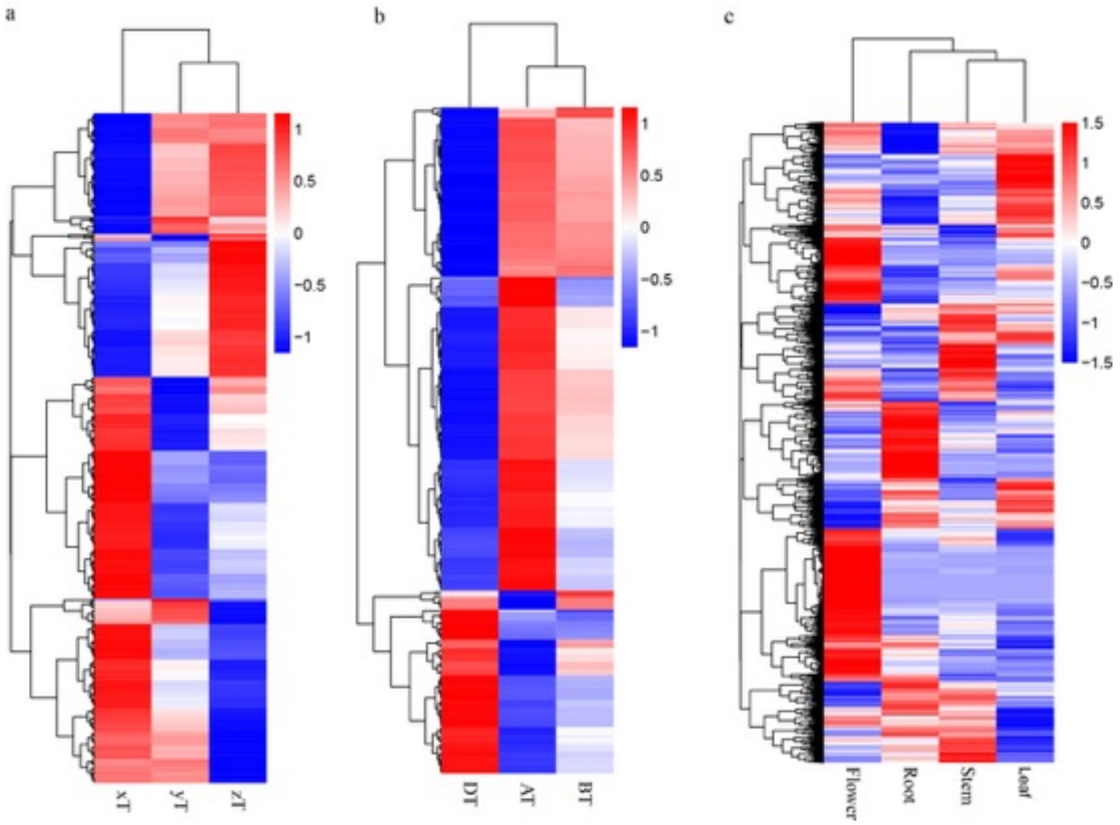

Supplementary Figure 20. The whole FPKM hierarchical clustering map, log10 (FPKM+1) values are normalized (scale number) and clustered, red indicates high expression gene, blue indicates low expression gene. The color ranges from red to blue, indicating log10 (FPKM+1) from large to small. (a) The RNA-Seq for 3 different growth stages (30d, 60d and 90d) of *C. sativum*. (b)The RNA-Seq for 3 different growth stages (30d, 60d and 90d) of *D. carota*. (c) The RNA-Seq for four tissues (root, stem, leaf, and flower) of *C. sativum*.

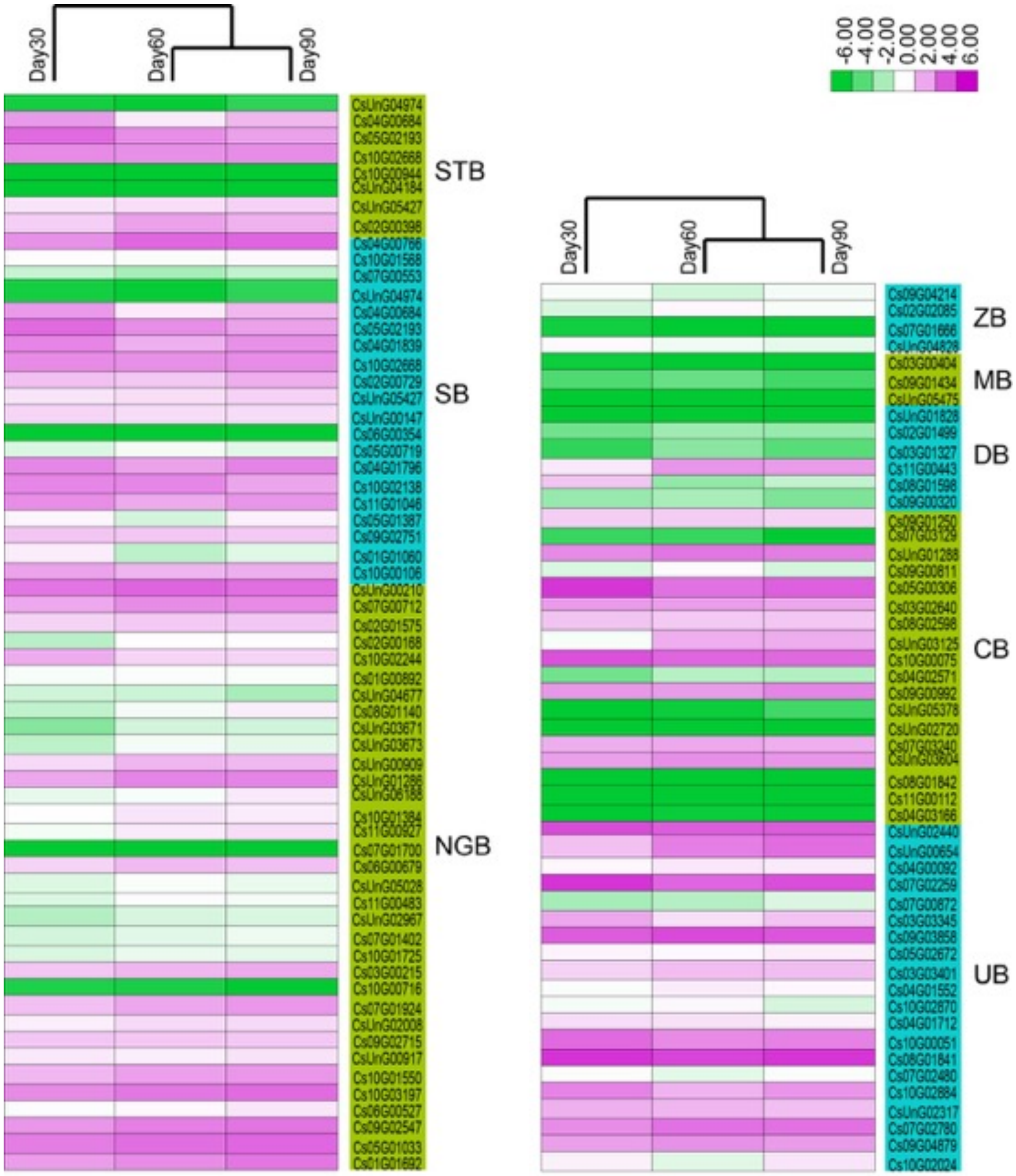

Supplementary Figure 21. The expression heatmap of 8 sub-biosynthesis pathways (STB, SB, NGB, ZB, MB, DB, CB, and UB) contained in the terpenoid backbone biosynthesis. The full name of 8 sub-biosynthesis pathways are list in the Supplementary Table 44. The genes expression was detected in the different development periods (30d, 60d, and 90d) after sowing of *C. sativum*. The log2 (FPKM+1) values are normalized and clustered, purple color indicates high expression gene, green color indicates low expression gene.

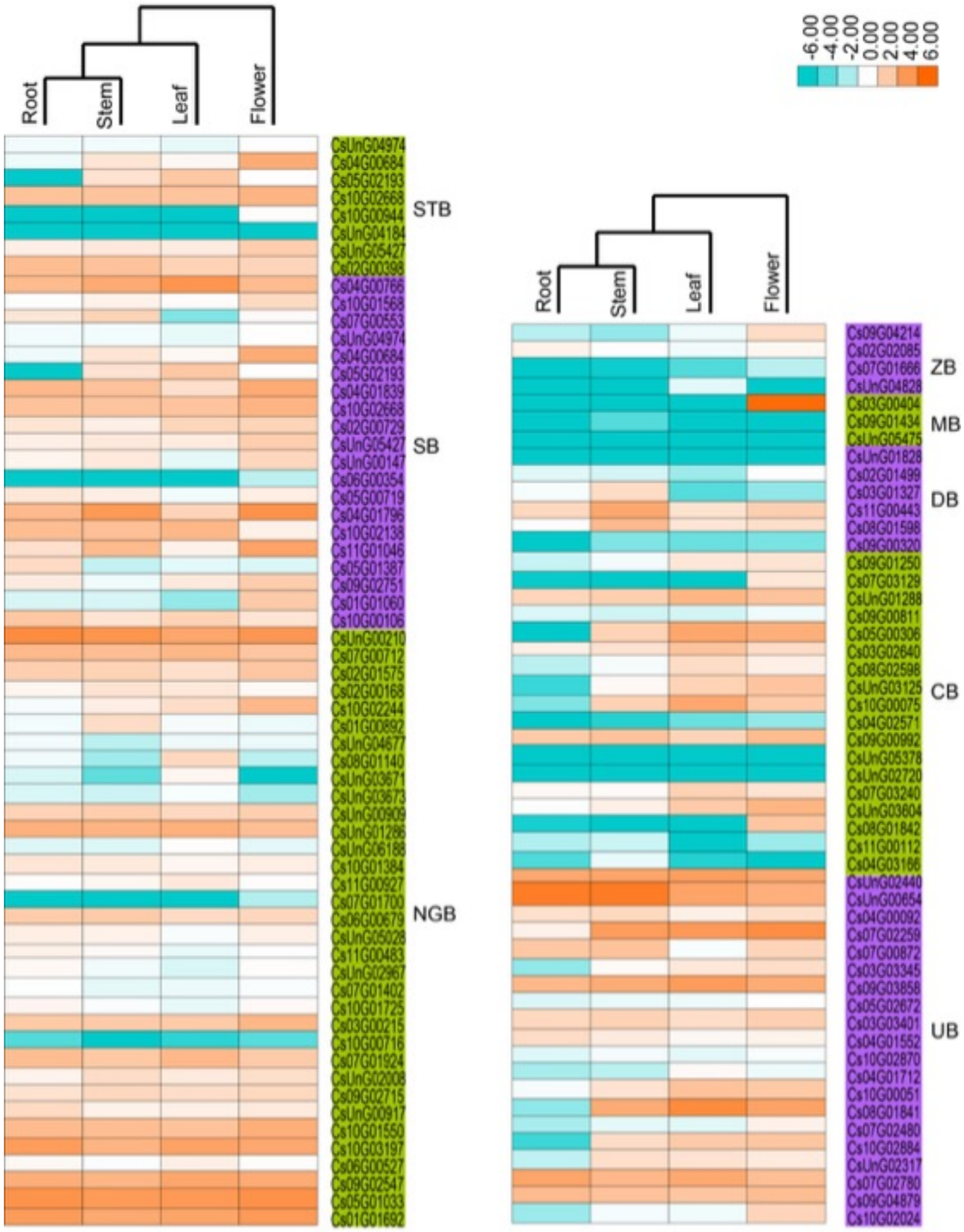

Supplementary Figure 22. The expression heatmap of 8 sub-biosynthesis pathways (STB, SB, NGB, ZB, MB, DB, CB, and UB) contained in the terpenoid backbone biosynthesis. The full name of 8 sub-biosynthesis pathways are list in the Supplementary Table 44. The genes expression was detected in the different tissues (root, stem, leaf, and flower) of *C. sativum*. The log2 (FPKM+1) values are normalized and clustered, purple color indicates high expression gene, green color indicates low expression gene.
