# Supplementary material for: Deciphering the high‐quality genome sequence of coriander that causes controversial feelings
Source: Plant Biotechnol J. 2020 Feb 5;18(6):1444–56. doi: 10.1111/pbi.13310 (PMC7206992; doi:10.1111/pbi.13310)
Supplement: Supplementary file 3 — Supinfo Supplementary Notes (including legends). [file PBI-18-1444-s003.pdf]

Deciphering the High Quality Genome Sequence of Coriander that Causes Controversial Feelings

Xiaoming Song[1,2,8], Jinpeng Wang[1,2,3,4,8], Nan Li[1,2], Jigao Yu[1], Fanbo Meng[1], Chendan Wei[1], Chao Liu[1], Wei Chen[1,2,5], Fulei Nie[1,2], Zhikang Zhang[1], Ke Gong[1], Xinyu Li[1], Jingjing Hu[1], Qihang Yang[1], Yuxian Li[1], Chunjin Li[1], Shuyan Feng[1], He Guo[1], Jiaqing Yuan[1], Qiaoying Pei[1], Tong Yu[1], Xi Kang[1], Wei Zhao[1], Tianyu Lei[1], Pengchuan Sun[1], Li Wang[1], Weina Ge[1], Di Guo[1], Xueqian Duan[1], Shaoqi Shen[1], Chunlin Cui[1], Ying Yu[1], Yangqin Xie[1], Jin Zhang[1], Yue Hou[1], Jianyu Wang[1], Jinyu Wang[1], Xiu-Qing Li[6], Andrew H. Paterson[7], Xiyin Wang[1,2,5*]

[1] School of Life Sciences, North China University of Science and Technology, Tangshan, Hebei 063210, China

[2]Center for Genomics and Computational Biology, North China University of Science and Technology, Tangshan, Hebei 063210, China

[3]State Key Laboratory of Systematic and Evolutionary Botany, Institute of Botany, Chinese Academy of Sciences, Beijing 100093, China

[4]University of Chinese Academy of Sciences, Beijing 100049, China

[5]School of Genomics and Bio-Big-Data, Chengdu University of Traditional Chinese Medicine, Chengdu 610075, China.

[6]Fredericton Research and Development Centre, Agriculture and Agri-Food Canada, Fredericton, New Brunswick, E3B 4Z7, Canada

[7]Plant Genome Mapping Laboratory, University of Georgia, Athens, GA, 30605, USA

[8]The authors contributed equally to the work.

*Correspondence should be addressed to Xiyin Wang: wangxiyin@vip.sina.com

Tel: 86-315-8805592

Running title: Deciphering Genome Sequence of Coriander

Directory

1. Survey of *C. sativum* genome 5

1.1 Introduction 5

1.2 Experimental method 5

1.3 Sequencing data output and quality control 5

1.3.1 Data description 5

1.3.2 Methods of data filtering 6

1.3.3 Quality control 6

1.4 K-mer analysis 7

1.4.1 K-mer analysis principle 7

1.4.2 K-mer analysis results 7

1.5 Conclusion 7

2. Preliminary genome assembly 7

2.1 Data error correction 7

2.2 10X genomics assisted third generation data assembly 8

2.3 Assembly results 8

2.3.1 Sequencing data statistics 9

2.3.2 Assembly result statistics 9

2.3.3 Genomic base composition 9

2.4 Assembly results evaluation 9

2.4.1 Sequence consistency assessment 9

2.4.2 Sequence integrity assessment 10

2.5 Conclusion 10

3. Hi-C technology assisted genome assembly 11

3.1 Introduction 11

3.2 Experimental procedure 11

3.2.1 Hi-C biotin labeling and genomic DNA extraction 11

3.2.2 Library construction 11

3.2.3 Library Check 11
3.2.4 Sequencing 12
3.3 Bioinformatics analysis 12
3.4 Sequencing data quality control 12
3.4.1 Original sequencing data 12
3.4.2 Sequencing data statistics 12
3.4.3 Sequencing data quality assessment 12
3.5 Hi-C technology assisted genome assembly 12
3.5.1 Comparison with draft genome 12
3.5.2 Clustering 13
3.5.3 Sorting and Orientation 13
3.5.4 Assembly result statistics 13
4. Genome prediction and annotation 13
4.1 Analysis process and method 13
4.1.1 Genome prediction process 14
4.1.2 Genome annotation analysis 14
4.2 Analysis results 15
4.2.1 Repeat sequence annotation 15
4.2.2 Gene structure annotation 16
4.2.3 Gene function annotation 16
4.2.4 Non-encoded RNA annotation 16
5. RNA-seq 17
5.1 Introduction 17
5.2 Database construction and sequencing 17
5.2.1 Total RNA sample detection 17
5.2.2 Library construction 17
5.2.3 Library inspection 18
5.2.4 Sequencing 18
5.3 Bioinformatic analysis 18

5.3.1 Oringinal sequences data 18
5.3.2 Sequencing data quality assessment 18
5.3.3 Alignment analysis of reference sequence... 19
5.3.4 Alternative splicing analysis 20
5.3.5 Novel transcript prediction 20
5.3.6 Gene expression level analysis 21
5.3.7 RNA-seq quality assessment... 21
5.3.8 Differential expression analysis 21
5.3.9 Differential gene GO enrichment analysis 22
5.3.10 Differential gene KEGG enrichment analysis 23
6 Comparative genomic analyses.. 23
6.1 Materials and Methods 23
6.1.1 Gene family identification and analysis.. 23
6.1.2 Phylogenetic analysis and divergence time estimation............................ 24
6.1.3 Gene family expansion and contraction analysis..................................... 24
6.1.4 Positive selection analysis... 25
6.1.5 Inference of gene collinearity 25
6.1.6 Construction of the event-related collinear gene table 26
6.1.7 Ks calculation, distribution fitting, and correction 26
6.1.8 Evolutionary tree construction.. 28
6.2 Results 28
6.2.1 Gene collinearity within and among genomes.. 28
6.2.2 Trajectory of two paleo-tetraploidization events 29
6.2.3 Distinguishing orthologous and out-paralogous regions 29
6.2.4 Trees of collinear genes support ancient paleo-tetraploidization 29
6.2.5 Multiple alignment.. 29
6.2.6 Local alignment 30
6.2.7 Genomic fractionation 30
6.2.8 Evolutionary rate divergence and dating 31
6.2.9 Positive selection analysis.. 32
6.2.10 Analysis of functional genes.. 32

1. Survey of *C. sativum* genome

1.1 Introduction

The survey was conducted for the coriander (*Coriandrum sativum*) genome size, heterozygosity rate, and repeat sequence ratio estimation. The coriander was diploid speices with the genome size about 2.484 Gb reported in the Kew website (https://www.kew.org). Here, we estimated the coriander genome size using Kmer, which is a popular method used nearly almost every species genome sequencing project (Marcais and Kingsford, 2011). In our study, we constructed two small fragment of the libraries, and then carried out Illumina HiSeq PE sequencing. The mainly analyses were about sequencing data quality control and genome feature assessment.

1.2 Experimental method

The qualified DNA sample was randomly interrupted into a length of 350 bp fragment using Covaris ultrasonic crusher. Then the fragment was repaired the end, added A Tail, plus sequencing joints, purification, PCR amplification steps to complete the entire library preparation. Finally, the well-constructed library was sequenced by Illumina HiSeq.

1.3 Sequencing data output and quality control

1.3.1 Data description

The production of sequencing data is through the DNA extracting, building, and sequencing multiple steps. However, the invalid data generated in these steps can cause serious interference to the advanced analysis of biological information data, such as the deviation of the length of the database during the construction phase, and the situation of sequencing errors in the sequencing phase. So, we must eliminate these ineffective data filtering by some methods to ensure the normal conduct of bioinformatics analysis.

1.3.1.1 Raw data

The original image data obtained by sequencing base calling into sequence data, which we called raw data or raw reads with the FASTQ format. FASTQ file was the original file, which contained the reads sequence and reads sequencing quality.

1.3.1.2 Clean data

The original sequencing data contained the adapter, low-quality bases, and an undefined base (N). These can cause significant disruption to subsequent bioinformatics analyses. Using filtering methods to remove these interference information, then the data is valid data, which we called clean data or clean reads. The finally file has the same data format as raw data.

1.3.2 Methods of data filtering

Filter mainly from the following three aspects:

1) The reads containing the adapter sequence needs to be filtered out;

2) When the content of N contained in single-ended sequencing read exceeds that 10% length of read.

3) The pair needs to be removed when the single-end sequencing read contains low quality (< 5) base exceeds 20% of the read length.

1.3.3 Quality control

1.3.3.1 Sequencing data statistics

We obtained the high quality clean data after a serials strict filtering of sequencing data. Furthermore, we statistic the output data, including sequencing read quantity, data yield, sequencing error rate, Q20 content, Q30 content, GC content and so on (Supplementary Table 1).

1.3.3.2 Sequencing quality assessment

1) GC content distribution check: This is mainly detection of GC separation phenomena. The proportion of A and T should be nearly equal, C and G should also be equal according to the principle of base complementarity. The N content reflects the quality of sequencing data.

2) Sequencing data quality check: The quality of sequencing data will be mainly distributed above Q20, so as to ensure the normal conduct of subsequent analysis. According to the characteristics of sequencing technology, the base quality at the end of sequencing fragment is generally lower than that of the front end.

3) Sequencing error rate distribution: The sequencing error rate is related to the quality of the base. According to the characteristics of sequencing technology, the error rate at the end of sequencing fragment will be high, and the error rate of large segment library sequencing is higher than that of small fragment library sequencing.

1.3.3.3 Sequencing data evaluation and conclusion

The original sequencing data of coriander samples is 139.87G. The sequencing data with the high quality (Q20≥90%, Q30≥85%), and sequencing error rate is low (<0.05%). The nucleotide library comparison results did not reveal any contamination in the sample.

1.4 K-mer analysis

1.4.1 K-mer analysis principle

Before genome assembly, genomic characteristics can be estimated by sequencing of sequences. We use K-mer method to estimate the genome size and hybridization rate, that is, from a continuous sequence to iteratively select the length of K base sequence. If the length of each sequence is L, then the k-mer length is K, then we can get the L-K+1 k-mer, here we take k= 17 to analyze.

1.4.2 K-mer analysis results

According to the results of the survey analysis, the main peak near depth=51(Supplementary Figure 1). The genome size obtained by (Kmer-number/depth) is about 2,151.47 Mb, and the corrected genome size is 2,130.29 Mb. The genomic heterozygosity rate was 0.47%, and the repeat sequence ratio was 80.58% (Supplementary Table 2).

1.5 Conclusion

The 139.87G sequencing data of coriander genome was analyzed by Kmer=17, and the estimated genome size was 2,151.47 Mb, corrected to 2,130.29 Mb, the heterozygosity rate was 0.47%, and the repeat sequence ratio was 80.58%. From the analysis of each indicator, it is indicated that the sequence of coriander genome is a complex genome, and the corresponding strategy can be used for further genome assembly.

2. Preliminary genome assembly

2.1 Data error correction

We can assume that the kmer with low frequency is due to sequencing errors when the amount of sequencing is large enough. The process of error correction is first establishment a kmer frequency table with some data. After setting cutoff, the kmer can be divided into high frequency and low frequency kmer. For reads with low-frequency

kmer, we can make the kmer of the entire reads high by changing some bases. Then we think that we have corrected some errors caused by sequencing. Because the large segment is in the process of cyclization during the construction of the library, and only plays a role in the assembly process. The large segments do not need to participate in this error correction process, so data correction is usually performed on small segment library data. The corrected data will be used for subsequent genome assembly.

2.2 10X genomics assisted third generation data assembly

(1) Extraction of genomic DNA (>50Kb)

(2) Third-generation database construction: 1) Third generation database: DNA adaptor with hairpin structure attached to both ends of double-stranded DNA. 2) First, the Pacbio data is self-corrected. Generally, the accuracy of the data can reach 99.999% after error correction. 3) Conducting the genome assemble using the third generation data after error correction. The assembly uses the Overlap-Layout-Consensus (OLC) algorithm, which is spliced by the overlap relationship between long reads. 4) For the assembly results obtained in the above step, all third generation data were sequenced for mapping. The assembly results were further corrected to improve the accuracy of the results, and finally obtained the contig sequence.

(3) 10X Genomics library construction: The gel beads are connected with: 1) illuminaP5 connector. 2) 16 base Barcode. 3) Illumina read 1 sequencing primers. 4) 10-bp random sequence primers.

The Barcode primer combines DNA and enzyme mixtures through two intersections, one at the intersection, and the second intersection with oil droplets, which are then collected and placed on a special 96-plate for 10X Genomics library preparation. After PCR amplification, break the oil droplets, mix different Barcode amplification sequences, break into appropriate fragments, and add P7 linker for illumina sequencing.

(4) Compare the obtained linked-reads with the contig of the third-generation sequencing.

(5) For contig/scaffold, where the actual distance is relatively close, there are many linked-reads that support their connection. For contig/scaffold, which is far away from actual distance, linked-reads support is missing and cannot be connected.

2.3 Assembly results

2.3.1 Sequencing data statistics

The coriander genome was sequenced using the third-generation sequencing technology Pacbio platform with a total sequencing capacity of 197.45 Gb, and a coverage depth of 92.69X (calculated according to the estimated genome size of 2,130.29 Mb). In addition, 10X Genomics library and second generation small fragments were constructed and sequenced using the illumina platform (Supplementary Table 3).

2.3.2 Assembly result statistics

Assembly results were selected from scaffolds above 100 bp. The contig N50 of the coriander genome reached 612.62 Kb, and the scaffold N50 reached 2.15 Mb (Table 1).

2.3.3 Genomic base composition

The results showed that the ratio of four bases A, T, G, and C is normal. The ratio of GC is 34.83%, and the ratio of N is 1.34%, which is an acceptable range (<10%) (Supplementary Table 4-5).

2.4 Assembly results evaluation

2.4.1 Sequence consistency assessment

In order to evaluate the accuracy of the assembly, the small fragment library reads were selected using BWA software (http://bio-bwa.sour ceforge.net/) to compare the assembled genomes (Jo and Koh, 2015), and the ratio of reads was counted. Cover the extent and depth of the genome, assess the integrity of the assembly and the uniformity of sequencing. The results showed that the alignment rate of all small fragments reads to the genome is about 97.98%, and the coverage rate is about 99.49%, indicating that the genomes of reads and assembly are well consistent (Supplementary Figure 2a, Supplementary Table 4).

Single Nucleotide Polymorphisms (SNPs) refer to genetic markers formed by single nucleotide variations in the genome, which are numerous and polymorphic. We use tools such as samtools (http://samtools.s ourceforge.net/) to sort the BWA alignment results by chromosome coordinates, remove duplicate reads, perform SNP Calling, and filter the original results to obtain SNP statistics (Etherington et al., 2015; Li et al., 2009). The ratio of SNP in the coriander genome is 0.0939%, and the ratio of homozygous SNP is 0.0005% (Supplementary Table 5). It is generally believed that the homozygous SNP ratio can reflect the correct rate of genome assembly. This result indicates that the assembly has a high single base correct rate.

The assembled genomic sequence was plotted with 10K for windows without recalculating GC content and mean depth. Based on this graph, the sequencing data can be analyzed for GC bias and contamination of the sample. The results showed that the sample is not contaminated according to the distribution of GC content and average depth. The GC content is concentrated around 35%, and there is no obvious separation of the scatter plots, indicating that it is no other external pollution in the genome (Supplementary Figure 2b).

2.4.2 Sequence integrity assessment

2.4.2.1 CEGMA assessment

The integrity of genome assembly is evaluated by the CEGMA, which is the abbreviation of Core Eukaryotic Genes Mapping Approach (http://korflab.ucdavis.edu/Datasets/) (Parra et al., 2007). The evaluation selected the conserved genes (248 Core Eukaryotic Genes) present in six eukaryotic model organisms to form a core gene library. Then, we combined several softwares, such as tblastn, genewise, and geneid to evaluate the assembled genome integrity (Birney et al., 2004). According to the statistical result, we assembled 239 Core Eukaryotic Genes with a ratio of 96.37% (Supplementary Table 6), indicating that the assembly was relative complete.

2.4.2.2 BUSCO assessment

We also used the Benchmarking Universal Single-Copy Orthologs (BUSCO, http://busco.ezlab.org/) program to evaluate the integrity of genome assembly (Seppey et al., 2019; Waterhouse et al., 2019). The evaluation of assembled genome by using a single-copy orthologous gene pool in conjunction with tblastn, augustus, and hmmer programs. According to the BUSCO assessment statistical results, 4, 956 orthologous single-copy genes assembled 94% of complete single-copy genes (Supplementary Table 7), indicating that the assembly results were complete.

2.5 Conclusion

Using 577.88G sequencing data of coriander genome, the sequencing depth was 271.27 X. Then the coriander genome was denovo assembly, and the results were as follows: contig total length 2,118.31 Mb, contig N50 length reached 612.62 Kb. The scaffold has a total length of 2,147.13 Mb, and the scaffold N50 has a length of 2.15 Mb. A variety of methods were used to evaluate the assembled genome, and the results

showed that the genome has a good consistency, integrity and accuracy.

3. Hi-C technology assisted genome assembly

3.1 Introduction

The Hi-C technology was used for assisted *C. sativum* genome assembly. The libraries were sequenced using Illumina HiSeq PE150. These analyses mainly contained the data quality control, mapping the reference genomes, clustering, sorting, orientation, accuracy assessment for the genome with chromosomal information.

3.2 Experimental procedure

3.2.1 Hi-C biotin labeling and genomic DNA extraction

(1) Using the cell cross-linking agent paraformaldehyde to make the DNA and the cell combined;

(2) After the cell lysis, using the restriction enzyme to deal with the cross-linked DNA, which cause a gap on both sides of the cross-linking point;

(3) At the end of the repairing, adding biotin label the end of the oligonucleotide;

(4) Using the nucleic acid ligase, make the adjacent DNA fragments linked;

(5) The protease digests the protein at the junction to de-crosslink the protein and DNA. Genomic DNA was extracted and the DNA was randomly broken into fragments of 350 bp by Covaris crusher and then recovered.

3.2.2 Library construction

Capture DNA with biotin under the adsorption of avidin magnetic beads, and follow the procedure to the DNA fragments. The steps of end-repair, addition of A, linker ligation, and PCR amplification and purify complete the entire library preparation. Constructed library was sequenced using Illumina HiSeq PE150.

3.2.3 Library Check

After the library was constructed, using Qubit 2.0 start preliminary quantification, and the library was diluted to 1 ng/μl. Then test the insert size of the library followed by Agilent 2100. If the insert size was as expected, starting accurate quantification to the effective concentration of the library by Q-PCR (the library effective concentration > 2 nM) to ensure the library quality.

3.2.4 Sequencing

After the library was qualified, the different libraries were pooled according to the effective concentration and the target data volume, and then using Illumina HiSeq PE150 to sequence.

3.3 Bioinformatics analysis

The main steps of Hi-C technology are as follows:

(1) Quality control of the raw data to obtain clean data;

(2) Mapping the clean data to the genome for comparison analysis;

(3) According to the comparison results, clustering, sorting, orienting, assisting the genome to anchor the chromosome.

3.4 Sequencing data quality control

3.4.1 Original sequencing data

Please refer to the section 1.3.1.

3.4.2 Sequencing data statistics

Please refer to the section 1.3.2.

3.4.3 Sequencing data quality assessment

The total of sequencing data for Hi-C is 278.90 Gb with the high sequencing quality (Q20 ≥ 90%, Q30 ≥ 85%). The GC distribution is normal, and the sample is not contaminated (Supplementary Table 8). The Hi-C construction library has a relative high quality. The finally valid read pairs was 3,385,763, and the data effect rate was 33.04% (Supplementary Table 9).

3.5 Hi-C technology assisted genome assembly

Hi-C technology obtained spatially connected DNA fragments, interactions between distantly located DNA fragments at physical locations by special experimental techniques. According to the interaction probability inside the chromosome is higher than that of between the two chromosomes, the contig or scaffold were divided into different chromosomes. According to the interaction probability decreases with the increase of the interaction distance on the same chromosome, sorting and orienting the contig or scaffold of the same chromosome.

3.5.1 Comparison with draft genome

Efficient high-quality sequencing data was compared to the draft genome by BWA software. We removed the repeat data and no paired data by SAMTOOLS (parameter:

rmdup), and obtained the high quality data (Etherington et al., 2015; Li et al., 2009). At the same time, we extracted the reads near the cleavage site for assisted genome assembly.

The sample alignment rate reflected the similarity between the sample sequencing data and the reference genome. The sequencing depth and coverage can directly reflect the homogeneity and the homology with the reference sequence. The alignment of the individual samples showed that their similarity to the draft genome met the requirements, while at the same time having very high sequencing depth and coverage.

3.5.2 Clustering

The short reads obtained by sequencing were first compared to the draft genome, and the reads were compared to contigs or scaffolds. If there were reads pairs captured by Hi-C technology on the two contigs, then there was an interaction between the two contigs. The more the number of reads interacting on two contigs, the stronger the interaction, and the more likely they were to be grouped together (Supplementary Figure 3a).

The number of reads with interaction between contigs was the number of interactions. The contigs were clustered according to the number of interactions, and the number of chromosomes of the species was divided into the specified number of classes.

3.5.3 Sorting and Orientation

According to the results of clustering grouping, the positions of the strengths of each two contig interactions and the interaction reads were sorted and oriented (Supplementary Figure 3b).

3.5.4 Assembly result statistics

Finally, a total of 1.774 Gb, accounting for 83.77% of the assembled genome, was anchored the 11 chromosomes by the Hi-C (Supplementary Table 10). The GC content was about 34.83%, accounting for the finally assembled genome (Supplementary Table 11).

4. Genome prediction and annotation

4.1 Analysis process and method

4.1.1 Genome prediction process

Structural prediction of genes usually involves multiple prediction methods, mainly homologous prediction, De novo prediction and other evidence-supported predictions. The method of homologous prediction is to compare the encoded protein sequence of a known homologous species with the genomic sequence of a new species (the number of homologous species is usually no more than 5), by blast (*http://blast.ncbi. nlm.nih.gov/Blast.cgi*), genewise (*http://www.ebi.ac.uk/~birney/wise2/*) and other comparison software predicts gene structure in the genome (Birney et al., 2004; Camacho et al., 2009). De novo predicts the use of software that relies on the statistical characteristics of genomic sequence data (such as codon frequency, exon-intron distribution) to predict gene structure. The commonly used softwares are Augustus (*http://bioinf.uni-greifswald.de/augustus/*), GlimmerHMM (*http://ccb.jhu.edu/software/glimmerhmm/*) (Stanke and Morgenstern, 2005), SNAP (*http://homepage.mac.com/iankorf/*), etc (Korf, 2004). Other evidence supports predictions that use EST or cDNA data from homologous species to predict gene structure by blat (*http://genome.ucsc.edu/cgi-bin/hgBlat*) (Kent, 2002). Combining the above prediction results and the transcriptome comparison data, the gene sets predicted by various methods are integrated into one non-redundant, and more complete gene set using the IntegrationModeler (*EVM*, *http://evidencemodeler.sourceforge.net/*) integration software (Haas et al., 2008). Finally, combined with the results of transcriptome assembly, the EVM annotation results were corrected using PASA (*http://pasa.sourceforge.net/*)(Haas et al., 2003). The UTR and variable splice information were added to obtain the final gene set.

4.1.2 Genome annotation analysis

The genome annotation mainly includes three aspects: repeated sequence annotation, gene annotation (including gene structure prediction and gene function prediction), and non-coding RNA (ncRNA) annotation.

The method of repetitive sequence annotation is divided into two types: homologous sequence alignment and de novo prediction. The homologous sequence alignment method is based on a repeat sequence database (RepBase, *http://www.girinst.org/repbase*), using the Repeatmasker and repeatproteinmask (*http://www.repeatmasker.org/*) software to

identify and know Repeat sequences with similar sequences (Bao et al., 2015; Tarailo-Graovac and Chen, 2009). The de novo prediction method firstly constructed the repeat sequence database by using LTR_FINDER (*http://tlife.fudan.edu.cn/ltr_finder/*)(Xu and Wang, 2007), Piler (*http://www.drive5.com/piler/*)(Edgar and Myers, 2005), RepeatScout (*http://www.repeatmasker.org/*)(Price et al., 2005), RepeatModeler (*http://www.repeatmasker.org/RepeatModeler.html*), then predicted by the Repeatmasker software. For the other method of de novo predictions, the TRF (*http://tandem.bu.edu/trf/trf.html*) software can detect tandem repeats in the genome(Benson, 1999).

We conducted the gene function annotation by comparing with a known protein database to obtain functional information of the gene. The Commonly protein databases are SwissProt (*http://www.uniprot.org/*) (Bairoch, 2005), TrEMBL (*http://www.uniprot.org/*)(Bairoch and Apweiler, 2000), KEGG (*http://www.genome.jp/kegg/*)(Ogata et al., 1999) and InterPro (*https://www.ebi.ac.uk/interpro/*)(Mulder and Apweiler, 2008).

Non-coding RNA annotations include tRNA, rRNA, miRNA and snRNA. According to the structural characteristics of tRNA, tRNAscan-SE (*http://lowelab.ucsc.edu/tRNAscan-SE/*) software is used to search for tRNA sequences in the genome (Chan and Lowe, 2019). Due to the rRNA is highly conserved, it is possible to select closely related species rRNA sequence as a reference sequence to search rRNA by Blast program. According to the Rfm family's covariance model, INFERNAL (*http://infernal.janelia.org/*) software was used to predict miRNAs and snRNAs (Nawrocki and Eddy, 2013).

4.2 Analysis results

4.2.1 Repeat sequence annotation

Repeat sequences can be divided into two major categories: tandem repeats and interspersed repeats. The tandem repeat sequence includes a microsatellite sequence, a small satellite sequence, etc. The scattered repeat sequence is also called a transposon element, and includes a DNA transposon and a retrotransposon transposed by DNA-DNA. Common retrotransposon classes are LTR, LINE and SINE. Based on the Lenovo repeat

sequence prediction tool and the existing repbase repeat sequence library, the cope seed genome was subjected to repeat annotation, and the results showed that the genome contained 70.59% of the repeat sequence (Supplementary Table 12). Furthermore, we classified the TEs, and the results showed that most of them belonged to the LTR (66.71%) (Supplementary Table 13).

Based on the alignment of the genome with Repbase, we plot the frequency of the different type repeat sequence (Supplementary Figure 4a).

4.2.2 Gene structure annotation

We conducted the denovo prediction of the genetic structure using the software Augustus, GlimmerHMM, SNAP, Geneid and Genscan. The homologous species include *A. thaliana*, *C. sativus*, *S. lycopersicum*, *S. tuberosum*, *D. Carota*, *O. sativa* and *L. sativa*. A total of 40747 genes were predicted in the coriander genome. The detailed statistical information is shown in Supplementary Table 14. Supplementary Figure 4b shows the support of each evidence for the gene set.

We further conducted the analyses of genetic structure in *C. sativum* and above mentioned 7 representative species. Among these examined species, *C. sativum* has the more genes (40,747) than other 7 species (Supplementary Table 15, Supplementary Figure 4c).

4.2.3 Gene function annotation

A total of 40,747 genes were predicted in the *C. sativus* genome (Table 5). The gene annotation was obtained by alignment of the known protein libraries, including NR, Swiss-Prot, KEGG, InterPro. Finally, a total of 37772 (92.7%) of the genes in the *C. sativus* genome can be predicted to function. Among of them, 25722 genes were annotated by all of these four databases (Supplementary Table 16, Supplementary Figure 5a).

4.2.4 Non-encoded RNA annotation

Non-coding RNA refers to RNA that does not translate proteins, such as rRNA, tRNA, snRNA, miRNA. miRNA can degrade its target gene or inhibit the translation of a target gene into a protein, and has the function of silencing the gene. The tRNA and rRNA are directly involved in the synthesis of proteins. The snRNA is mainly involved in the processing of RNA precursors and is the main component of RNA splicing. The ncRNA information of the coriander genome obtained by comparison with known

ncRNA libraries or structural prediction is shown in Supplementary Table 17.

5. RNA-seq

5.1 Introduction

The samples of *C. sativum* and *D. carota* collected from 3 different growth stages, including 30d, 60d and 90d after sowing. In addition, the four tissues (root, stem, leaf, and flower) of *C. sativum* were also used for RNA-Seq analyses. Each sample was set as three replications. The RNA was isolated from leaves using RNA kit (Tiangen, Beijing, China) according to manufacturer's instructions.

5.2 Database construction and sequencing

From the RNA sample to the final data acquisition, each step of sample detection, database construction and sequencing will have an effect on the quality and quantity of the data. The quality of data will directly affect the results of subsequent analysis. In order to guarantee the accuracy and reliability of the sequencing data, we strictly control each step of sample detection, database construction and sequencing, which can radically ensure the high quality data.

5.2.1 Total RNA sample detection

The following four methods for RNA sample detection:

(1) Agarose Gel Electrophoresis analyses the degree of RNA degradation and test whether existing contamination or not.

(2) Nanodrop test the purity of RNA（OD260/280）.

(3) Qubit accurately quantified RNA concentration.

(4) Agilent 2100 accurately detects RNA integrity.

5.2.2 Library construction

When the samples were qualified, using the magnetic beads with Oligo (dT) to enrich the Eukaryote mRNA by the base A-T pairing and the combination of the mRNA ploy A tail. After, adding fragmentation buffer is to break mRNA into short fragments. Next using mRNA as a template, a single-strand cDNA was synthesized by using random hexamers, and then the double-stranded cDNA was synthesized by adding buffer, dNTPs and DNA polymerase Ⅰ. Finally using AMPure XP beads purified double-stranded

cDNA. The purified double-stranded cDNA was subjected to terminal repairing. After adding tail A and connecting the sequencing linker, adopting AM Pure XP beads choose the size of fragments. Last, performing PCR enrichment was to obtain the final cDNA library.

5.2.3 Library inspection

When library construction was finished, we needed taking on preliminary quantification by using Qubit 2.0, and the library was diluted until 1 ng/ul. Then, using Agilent 2100 detected the insert size of the library. If the insert size was as expected, using Q-PCR to take on accurate quantification for the effective concentration of the library (the library's effective concentration> 2 nM) to ensure the library's quality.

5.2.4 Sequencing

After library inspection was qualified, we needed to take on HiSeq sequencing for the different libraries according to the effective concentration and the target data volume which were pooled.

5.3 Bioinformatic analysis

After obtaining the sequenced reads and referencing the relevant species and the genome, the bioinformatics analysis processing as follows:

5.3.1 Oringinal sequences data

The original image data files obtained by High-throughput sequencing (Illumina HiSeq^{TM}) transformed the original sequencing sequences after analyzed by CASAVA Base Calling. We called it Raw Data or Raw Reads. The results stored were stored in FASTQ file format. It contained the sequencing (reads) information and its corresponding sequencing quality information.

5.3.2 Sequencing data quality assessment

5.3.2.1 Check the distribution of sequence error rate

The error rate of each base sequencing is obtained by the Phred score (Formula 1: Qphred = -10log10(e)). The Phred value is obtained by a rate model during the base call (Base Calling) process. The model can predict accurately the error rate of the base calling. The sequencing error rate is related to the base quality and is affected by many factors such as the sequencer itself, sequencing reagents, and samples.

5.3.2.2 Check A/T/G/C content

The GC content distribution test is used to detect the phenomenon whether exist

separation between AT and GC or not. This phenomenon may be caused by sequencing or library construction and may affect subsequent quantitative analysis.

In the transcriptome sequencing at the Illumina sequencing platform, the 6 bp random primer used in reverse transcription into cDNA causes a preference for the first few positions nucleotide composition. This preference has nothing to do with the sequenced species and laboratory environment, but it can affect the degree of homogeneity of transcriptome sequencing (Hansen et al., 2012). In addition, in each sequencing circle, the G and C bases and A and T base contents should be equal, and the whole sequencing process is stable and horizontal. But for the Strand-Specific library construction, it exists the G and C base separation. For Illumina sequencing, due to random primer amplification bias and other reasons, it is normal that the first six-seven base for each read appear fluctuation.

5.3.2.3 Sequencing data filtering

The original sequencing sequence from sequencing contains low-quality reads with connectors. In order to ensure the quality of information analysis, we must filter to the raw reads and then gain the clean reads. And the subsequent analysis is based on the clean reads.

The steps for data processing are as follows:

(1) Removing the reader with adapter;

(2) Removing N (N means that the base information not clear) the proportion of reads lager than 10%;

(3) Remove the low-quality reads (the bases of Qphred <= 20 account for more than 50% of the whole read length of reads).

5.3.2.4 Summary of sequencing data quality

The clean data of four tissues (root, stem, leaf, and flower) of *C. sativum* were totally 79.26Gb (Supplementary Table 34). The clean data of 3 different growth stages (30d, 60d and 90d) of *C. sativum* and *D. carota* were totally 69.22Gb and 68.70Gb, respectively (Supplementary Table 35).

5.3.3 Alignment analysis of reference sequence

We select the software HISAT in order to perform genomic positioning analysis for the filtered sequence (Kim et al., 2015). HISAT can effectively compare the spliced reads

in RNA-Seq sequencing data, which is the highest and most accurate alignment software.

The total mapped ratios of four tissues were almost more than 90%, and the uniquely mapped ratios were more than 80% (Supplementary Table 36). Similar, there was the same trends for the 3 different growth stages (30d, 60d and 90d) of *C. sativum* and *D. carota* (Supplementary Table 37-38). This indicated that there was no contamination and the reference genome is selected appropriately.

5.3.4 Alternative splicing analysis

Alternative Splicing (AS) is a common expression pattern in most eukaryotic cells. After the gene is transcribed into an mRNA precursor, the intron is removed by the RNA cleavage, while the exon is retained in the mature mRNA. An RNA can have multiple exon splicing forms, thus allowing a gene to translate different proteins at different times and in different environments, thereby increasing the complexity or adaptability of the system under physiological conditions.

rMATS (http://rnaseq-mats.sourceforge.net/index.html) is a variable AS analysis software for RNA-Seq data (Shen et al., 2014). It can not only classify AS events, but also perform differential analysis of AS events between different samples. For each comparison group that performs differential shear analysis, we first count the types and quantities of AS events that occur. Then we calculate the expression levels of each type of variable shear events, and finally variable for each type. The AS event is analyzed for difference. In the quantification process, rMATs took two quantitative methods: Junction Count only, reads on target and junction counts. The difference between them is that the Junction Count only quantifies the reads that are all aligned to the Alternative spliced exon. We performed a difference analysis for each type of AS event. The FDR < 0.05 is used as the screening criterion for the differential AS event.

5.3.5 Novel transcript prediction

Put together the genomic localization results of all sequencing reads data, assemble them with Cufflinks (Trapnell et al., 2010), and then compare them with known gene models using Cuffcompare, which can: (1) discover new genes (relative to the original gene annotation files); (2) Discover new exon regions of known genes; (3) Optimize the initiation and termination positions of known genes. The new gene and new exon region prediction results are annotation files in GTF format.

5.3.6 Gene expression level analysis

In RNA-seq analysis, we estimate gene expression levels by counting the number of sequencing reads that are located in the genomic region or exon region. The Reads count is not only related to the length of the gene and the depth of sequencing, but also can able to comprise to the true expression level of the gene. In order to make the different genes and different experiments comparable, FPKM is currently the most commonly used method for estimating gene expression levels (Trapnell et al., 2010), which expected number of Fragments Per Kilobase of transcript sequence per Millions base pairs sequenced, and took into account the effect of sequencing depth and gene length (Supplementary Table 39-41).

We adopt the model union and the HTSeq software to analysis the gene expression level (Anders et al., 2015). In general, the FPKM value of 0.1 or 1 is used as a threshold for judging whether or not a gene is expressed. We compare the gene expression levels under different experimental conditions by FPKM profiles of all genes. For replicate samples under the same experimental conditions, the final FPKM is the average of all replicates.

5.3.7 RNA-seq quality assessment

The correlation of gene expression levels between samples is an important indicator to test the reliability of the experiment and whether the sample selection is reasonable. The closer the correlation coefficient is to 1, the higher the similarity in expression patterns between samples. Here, we require that the biological repeat sample R2 be at least greater than 0.8 (Supplementary Figure 19).

5.3.8 Differential expression analysis

5.3.8.1 Identification of differentially expressed genes

The input data of gene differential expression analysis is the readcount data obtained in the gene expression level analysis. The analysis is mainly divided into three parts:

1) Normalize the readcount;

2) Calculating the hypothesis test probability (pvalue) according to the model;

3) Finally, multiple hypothesis test calibration is performed to obtain the FDR value (error discovery rate). We used the DESeq software to conduct the DEGs analyses with the padj<0.05 (Anders and Huber, 2010).

5.3.8.2 Differential gene cluster analysis

Cluster analysis is used to determine the expression patterns of differential genes under different experimental conditions; genes with similar expression patterns may have similar functions or participate in the same metabolic process or cellular pathway. Therefore, by clustering genes with the same or similar expression patterns, it can be used to speculate on the function of an unknown gene or the new function of a known gene. The FPKM values of differential genes under different experimental conditions were used for hierarchical clustering analysis (Supplementary Figure 20). Different color regions represented different clustering group information. The gene expression patterns in the same group were similar and may have similarities. Function or participate in the same biological process.

In addition to the differential gene expression FPKM hierarchical cluster analysis, we also clustered the relative expression level values of log2 (ratios) of the differential genes by three methods: H-cluster, K-means and SOM. Different clustering algorithms divide the differential gene into several clusters, and the genes in the same cluster have similar expression levels under different processing conditions.

5.3.9 Differential gene GO enrichment analysis

Gene Ontology (GO, http://www.geneontology.org/) is an international standard classification system for gene function. GO is divided into three parts: molecular function (Molecular Function), biological process (Biological Process), and cell composition (Cellular Component). The principle of GO enrichment analysis is hypergeometric distribution. The hypergeometric distribution relationship between these differential genes and some specific branches in the GO classification is calculated according to the selected differential genes, and a specific p-value is obtained through hypothesis verification.

The software used in our analysis of GO enrichment analysis is GOseq (Young et al., 2010), which is based on the Wallenius non-central hyper-geometric distribution. Compared to the ordinary hyper-geometric distribution, this distribution is characterized by the fact that the probability of extracting an individual from a certain category is different from the probability of extracting an individual from outside a certain category, and the probability is different. It is estimated by estimating the length of the gene, so that the probability of GO term being enriched by differential genes can be calculated more

accurately.

5.3.10 Differential gene KEGG enrichment analysis

Pathway significant enrichment analyses can determine the most important biochemical metabolic pathways and signal transduction pathways involved in differentially expressed genes. KEGG (Kyoto Encyclopedia of Genes and Genomes) is a system for analyzing gene function and genomic information databases. It provides excellent integration of metabolic pathways, including metabolism of carbohydrates, nucleosides, amino acids, etc. Pathway significant enrichment analysis uses hypergeometric tests, and find pathway that was significantly enriched in differentially expressed genes compared to the entire genome background. It indicates that the differential gene is significantly enriched in the pathway when FDR $\leq$ 0.05. We used KOBAS (2.0) for pathway enrichment analysis (Xie et al., 2011).

6 Comparative genomic analyses

6.1 Materials and Methods

6.1.1 Gene family identification and analysis

Gene family is a group of genes that are derived from the same ancestor and consist of two or more copies of a gene through gene duplication and species differences. They have distinct similarities in structural and function, encoding similar proteins product. The identification of gene families is an important aspect of evolutionary analysis. The OrthoMCL (http://orthomcl.org/orthomcl/) process is used for gene family identification in this study (Fischer et al., 2011). The specific steps are as follows:

Step 1: Filter the gene set of each species. Firstly, when a gene has multiple alternative splicing transcripts, only the longest transcript in the coding region is retained for further analysi. Secondly, excluded genes that encode proteins less than 50 amino acids.

Step 2: Obtain the similarity relationship between protein sequences of all species by blastp, and the e-value defaults to 1e-5.

Step3: Use OrthoMCL software to compare the results and cluster the results using 1.5 expansion coefficient.

Then, the single-copy gene families and multi-copy gene families can be obtained,

which are relatively conserved among species. Single-copy gene family refers to a gene family with only one gene copy in all species. Multi-copy gene family refers to the large number of duplication of the genomes during evolution. Some of these repetitive DNA sequences continue to turn out evolutionary differences and become new genes that are different from the original sequences. While some of them are remained in the form of structure and function that remain essentially the same, and become multi-copy gene families.

6.1.2 Phylogenetic analysis and divergence time estimation

In the study of biological evolution and systematic classification, a tree-like branch is commonly used to summarize the kinship between various organisms. The graph of this tree branch becomes a phylogenetic tree, and one of the main content of phylogenetic analysis is the construction of a phylogenetic tree. When constructing the phylogenetic tree, we performed multiple sequence alignments on all single-copy genes, and then combined all the alignment results to construct a phylogenetic tree called super alignment matrix. Here, we performed the construction of 13 species phylogenetic trees (ML TREE) by the maximum likelihood method using RAxML software (http://sco.h-its.org/exelixis/web/software/raxml/index.html) (Stamatakis, 2014).

Finally, we identified 519 single-copy gene families and used to estimate divergence time using mcmctree (http://abacus.gene.ucl.ac.uk/software/paml.html) in the PAML software package (Yang, 1997; Yang, 2007). The time correction points are as follows, *C. sativum* and *M. truncatula* (107-*125Mya*), *B. rapa* and *P. trichocarpa* (107.0-109.0Mya), *B. rapa* and *M. truncatula* (107-109Mya), *O. sativa* and *N. nucifera* (140- 200Mya), *B. rapa* and *A. thaliana* (20.4-30.9Mya). The time correction points are all taken from the TimeTree website (http://www.timetree.org/) (Kumar et al., 2017). The parameters of mcmctree are set as follows, burn-in=5,000,000, sample-number=1,000,000, sample-frequency=50.

6.1.3 Gene family expansion and contraction analysis

Expansion and contraction of a gene family means that a gene family exhibits significant differences in the number of genes among one or several species (more genes represent expansion, and less genes represent contraction). The gene family expansion and contraction analysis based on mathematical statistical tests. Based on the cluster

analysis results of the gene family, and filtering the gene family with abnormal gene numbers in individual species. The gene family expansion and contraction analysis were performed using CAFE software (http://sourceforge.net/projects/cafehahnlab/)(De Bie et al., 2006).

6.1.4 Positive selection analysis

The probability of positively selected is detected by calculating the Ka/Ks using the maximum likelihood ratio. Ka/Ks refers to the ratio of non-synonymous mutation rate to synonymous mutation rate. Ka/Ks>1, it is considered to have positive selection effect; Ka/Ks=1, it is considered to have neutral selection; Ka/Ks <1, it is considered that there is a purification selection effect. Multi-sequence alignment of protein sequences of single-copy gene families of species in the selection analysis was performed by MUSCLE software. The protein sequence alignment results were filtered by Gblocks software (http://molevol.cmima.csic.es/castresana/Gblocks.html) to remove the low-quality alignment region (Talavera and Castresana, 2007). Then, the multi-sequence alignment result of the corresponding CDS is generated by using the filtered protein sequence alignment result as a template. For each gene family, we detect whether the gene family was positively selected in the foreground branch by using a branch-site model in the codeml tool in PAML. The likelihood ratio test of two hypotheses is being chosen to determine if there is a positive selection rather than simply looking for a gene with ka/ks >1.

6.1.5 Inference of gene collinearity

Genomic sequences and annotations of referring plants, including grape, coffee, lettuce, and carrot.

Collinear genes were inferred using ColinearScan (Wang et al., 2006), a statistically well-supported algorithm and software. Blastp search was performed to find putative homologous genes within a genome or between genomes. A loose criterion was used to perform the search, with E-value set to be >= 1e-5. To our experience, a loose threshold here will accommodate the much diverged collinear genes and their fast and unbalanced divergence, but will not jeopardy the inference of genomic homology, in that homologs produced by paleo-polyploidy tens of million years ago could be much diverged. When running ColinearScan, Maximal gap length between genes in collinearity along a

chromosome sequence was set to be 50 genes apart, which was also used in many previous publications (Wang et al., 2017a; Wang et al., 2017b; Wang et al., 2016a; Wang et al., 2016b; Wang et al., 2005; Wang et al., 2015). For large gene families leads to difficulty to infer gene colinearity, familiar with > 30 genes were removed from the analysis before running ColinearScan.

To see directly the homology within and between genomes, homologous gene dotplots were produced using MCSCANX toolkits (Wang et al., 2012). Dotplots were used to aid to find homologous blocks produced by different polyploidization events (Wang et al., 2017a). Synoymous nucleotide substitution rates (Ks) were estimated between homologous genes, and with the Ks median of a collinear block was shown in the dotplots to help to group blocks produced by different events.

6.1.6 Construction of the event-related collinear gene table

To construct the table with the grape genome as a reference, all grape genes were listed in the first column. Each grape gene may have two extra collinear genes in its genome due to hexaploidy, and we assigned two other columns in the table to list this information. For a grape gene, when there was a corresponding collinear gene in an expected location, a gene ID was filled in a cell of the corresponding column in the table. When it was missing, often due to gene loss or translocation in the genome, we filled in the cell with a dot. For the coffee genome, without extra duplications, we assigned one column. While for the carrot or coriander genome, each affected by the two paleo-terapoidization events, we assigned four columns. Therefore, the table had 39 columns, reflecting layers and layers of tripled and then fourfold homology due to recursive polyploidies across the genomes. The coffee genome as the reference was constructed similarly.

6.1.7 Ks calculation, distribution fitting, and correction

Synonymous nucleotide substitutions on synonymous sites (Ks) were estimated by using the Nei-Gojobori approach (Nei and Gojobori 1986) implemented by using the Bioperl Statistical module.

Kernel smoothing density function **ksdensity** (width is generally set to 0.05) in Matlab was used to estimate the probability density of each Ks list to obtain the density distribution curve. Then, Gaussian multi-peak fitting of the curve was inferred by using

the gaussian approximation function **Gaussian** in the fitting toolbox **cftool**. We set $R\text{-}squared$, a parameter to evaluate the fitting goodness, to be at least 95%, used the smallest number of normal distributions to represent the complex Ks distribution, and the principle one was used to represent the corresponding evolutionary event.

To correct the evolutionary rates of ECH-produced duplicated genes, the Maximum likelihood Estimate μ from inferred Ks means of ECH-produced duplicated genes were aligned to have the same value of that of grape, which has been evolved the slowest. Supposing a grape duplicated gene pair to have Ks value is a random variable $X_G \sim \left(\mu_G,\ \sigma_G{}^2\right)$, and for a duplicated gene pair in another genome the Ks to be $X_i \sim \left(\mu_i,\ \sigma_i{}^2\right)$, we got the relative difference:

$$r = (\mu_i - \mu_G)\ /\ \mu_G.$$

To get the corrected $X_{i-correction} \sim \left(\mu_{i-corrrction},\ \sigma^2{}_{i-correction}\right)$, we defined the correction coefficient as:

$$\frac{\mu_{i-correction}}{\mu_i} = \frac{\mu_G}{\mu_i} = \lambda_i,$$

and $\mu_{i-correction} = \dfrac{\mu_G}{\mu_i} \times \mu_i = \dfrac{1}{1+r} \times \mu_i.$

$$\lambda_i = \frac{1}{1+r}$$

then,

$$X_{i-correction} \sim \left(\lambda_i \mu_i,\ \lambda_i^2 \sigma_i^2\right)$$

To calculate Ks of homologous gene pairs between two plants, i, j, suppose the Ks distribution is $X_{ij} \sim \left(\mu_{ij},\ \sigma_{ij}{}^2\right)$, we adopted the algebraic mean of the correction coefficients from two plants,

$$\lambda_{ij} = (\lambda_i + \lambda_j)\ /\ 2,$$

then,

$$X_{ij-correction} \sim \left(\lambda_{ij} \mu_{ij},\ \lambda_{ij}^2 \sigma_{ij}^2\right).$$

Specifically, when one the plant is grape, for the other plant, *i*, we have

$$X_{iG-correction} \sim \left(\lambda_i \mu_{iG},\ \lambda_i^2 \sigma_{iG}^2\right).$$

Based on the fact that carrot and coriander shared two extra polyploidizations after the split with lettuce, and the different evolutionary rates of these two polyploidizations, we need to re-correct their evolutionary rates to keep the same pace. Here, accoding to the result that coriander with the slower rate during both the two extra polyploidizations, we re-corrected the evolutionary rates suffered in carrot with coriander as the reference. The specific way to re-correct the evolutionary rates related to the extra events just fitted the above re-corrections of the ECH events.

6.1.8 Evolutionary tree construction

Trees of homologous genes in three genomes were constructed by implementing the maximal likelihood approach in PHYML (Guindon et al., 2005) and the neighboring-joining approach in PHYLIP using default parameter settings (Retief, 2000; Shimada and Nishida, 2017).

6.2 Results

6.2.1 Gene collinearity within and among genomes

Homologous collinearity of existing genomes is an important clue to reveal the evolution of complex genomes. Using ColinearScan (Wang et al., 2006), we inferred collinear genes within and between coriander and other reference genomes, which provides a function for evaluating the statistical significance of blocks of colinear genes (Supplementary Table 18-19, Supplementary Figure 6). For the blocks with four or more colinear genes, we found the most duplicated genes in coriander (7,214 pairs), and the fewest in grape (1,895 pairs) **(**Supplementary Table 18**)**. The blocks reside in coriander is similar to that in the carrot genome, and there are more collinearity regions in the coriander than in other genomes. For the collinear regions contain more than 10 gene pairs, coriander (3,829 pairs reside in 186 blocks) has larger number than grape and coffee have 1,232 pairs and 1,301 pairs reside in 54, and 45 blocks, respectively. This suggests that coriander may have experienced more polyploidy events.

In addition, the results also indicated that the collinearity between genomes is much better than within genomes (Supplementary Table 18-19). For example, there were only 84 colinear gene pairs reside in longest duplicated block in coriander. However, 210, 886,

101, 121 colinear gene pairs reside in longest duplicated block between coriander and coffee, carrot, lettuce, and grape, respectively (Supplementary Table 18). Therefore, based on the above comparisons, we can get a clearer understanding of the polyploid scale of coriander by comparing the homologous structure between the coriander and the reference genome.

6.2.2 Trajectory of two paleo-tetraploidization events

By constructing the homologous dotplot between genomes (Supplementary Figure 7-10), and comparing the homologous chromosome regions of coriander, grape and coffee, we found that after the differentiation of coriander and coffee, two consecutive whole genome tetraploid events have occurred.

6.2.3 Distinguishing orthologous and out-paralogous regions

Through genome structure analysis, we distinguished the orthologous and out-paralogous, and the correspondence of orthology chromosomes have displayed (Supplementary Table 20-22,25-27).

6.2.4 Trees of collinear genes support ancient paleo-tetraploidization

We constructed 524 and 683 groups of homologous gene evolutionary trees with one grape gene and one coffee gene as outgroups, each containing at least two carrot genes and at least two coriander genes. In the homologous gene trees with grape and coffee as the outgroup, 78.1% (409/524) and 76.9% (525/683) respectively correspond to the expected topology (Supplementary Figure 18).

6.2.5 Multiple alignment

With the grape genome as a reference, we produced a table to store inter- and intra-genomic homology information. First, we filled in all grape gene IDs in the first column of the table, then added gene IDs from coffee and other genome column by column, species by species according to the colinearity inferred by multiple alignments. As noted above, in the absence of gene loss the grape genes would have one colinear orthologous genes in coffee, 3 orthologous genes in lettuce, and 4 in coriander and carrot. When a legume species contained a gene showing colinearity with a grape gene, a gene ID was filled into an appropriate cell in the table. When a legume species did not have an expected colinear gene, often due to gene loss or translocation or insufficient assembly, a dot (signifying missing) was filled into an appropriate cell. For grape, coffee, lettuce, coriander, and carrot there have 13 (1+1+3+4x2) columns in the table. Moreover, due to

the ECH, each chromosomal segment would repeat three times in each genome. Based on homology inferred in grape, we therefore extended the table to 39 columns. Finally, we constructed a table of colinear genes reflecting three polyploidizations and all salient speciations. In partial summary, the table summarized results of multiple-genome and event-related alignment, reflecting layers of tripled and/or doubled homology due to recursive polyploidizations (Figure 4).

The genomic alignment table for four genomes with grape as a reference is not complete – in particular, it cannot include genes retained or newly generated in coffee. That is, genes specific to coffee and absent from the grape genome are not represented. Therefore, the grape-reference homology table was supplemented by a genomic homology table with coffee as reference (Supplementary Figure 12).

6.2.6 Local alignment

Using as reference the grape chromosomes 8, 6 and 13, which were produced by the ECH, we displayed the alignment of a region from 12.3 to 13.5 Mb on grape chromosome 8, 4.1 to 5.5 Mb on chromosome 6, 1.7 to 3.1 Mb on chromosome 13, along with its corresponding regions from all other genomes. Chromosome numbers are shown after the names of plants, and locations on chromosomes also are shown. The gene is shown by a rectangle and its position corresponds to the position of the gene that is collinear on chromosomes 8, 6 and 13 of the grape (Figure 4a,b).

In addition, using as reference the coffee chromosomes 6, 8 and 4, which were produced by the ECH, we displayed the alignment of a region from 8.2 to 8.8 Mb on coffee chromosome 6, 24.1 to 27.2 Mb on coffee chromosome 8, 1.8 to 2.2 Mb on coffee chromosome 4, along with its corresponding regions from all other genomes. Chromosome numbers are shown after the names of plants, and locations on chromosomes also are shown. The gene is shown by a rectangle and its position corresponds to the position of the gene that is collinear on chromosomes 6, 8 and 4 of the coffee (Supplementary Figure 13).

6.2.7 Genomic fractionation

We *analyses the Coriandrum sativum* gene loss rates and gene translocation compare with the grape, coffee, and carrot. *Using the* grape as reference genome, *Coriandrum sativum* gene loss rates from 0.59 (grape chromosomes 8) to 0.77 (grape chromosomes 16)

(Supplementary Table 31, Supplementary Figure 14a). *Using the* coffee as reference genome, *Coriandrum sativum* gene loss rates from 0.51 (coffee chromosomes 6) to 0.66 (coffee chromosomes 9) (Supplementary Table 32, Supplementary Figure 14b). *Using the* carrot as reference genome, *Coriandrum sativum* gene loss rates from 0.46(carrot chromosomes 6) to 0.64 (carrot chromosomes 9) (Supplementary Table 33, Supplementary Figure 14c).

Furthermore, the observed distribution of gene loss and translocation numbers were fitted by using different density curves of geometry distribution (Supplementary Figure 14). The F-test was used, and the P-value were 0.893, 0.903, and 0.915 for *C. sativum* compared with coffee, carrot, and grape, respectively (Supplementary Table 28). The retation of duplicated genes reside in *C. sativum* subgenome was detected using the the grape, coffee, and carrot as references, respectively (Supplementary Figure 15-17).

6.2.8 Evolutionary rate divergence and dating

We characterized the synonymous substitution divergence (Ks) between each colinear gene pair, which showed a clear bimodal structure with two distinct sets, one with Ks distribution peaking at 0.40 (±0.31) and another peaking at 0.75(±0.53) (Figure 3c), indicating at least two large-scale genomic duplication events (Supplementary Figure 11a,b; Supplementary Table 23). We also inferred colinear genes and characterized Ks distribution in other plant genomes. The peaks with larger Ks values in all grape, coffee, lettuce, and carrot genomes correspond to the ECH, as repeatedly reported previously (Jaillon et al., 2007; Paterson et al., 2012; Wang et al., 2016).

To date the hexaploidization event in the coriander lineage, we performed evolutionary rate correction to the evolutionary rates of coffee, lettuce and carrot duplicates (Supplementary Figure 11c,d; Supplementary Table 24). Here, different from previous practice (Wang et al., 2015a, 2017), we performed a two-step rate correction. Based on the fact that carrot and coriander shared two extra polyploidizations after the split with lettuce, and the different evolutionary rates of these two polyploidizations, so we conducted two rounds correction of their evolutionary rates to keep the same pace. In the first step, we managed to correct evolutionary rate by aligning the Ks distributions of coriander, coffee, lettuce and carrot ECH duplicates to that of grape ECH duplicates, which have the smallest Ks values. Then, accoding to the result that coriander with the

slower rate during both the two extra polyploidizations, we re-corrected the evolutionary rates suffered in carrot with coriander as the reference. The specific way to re-correct the evolutionary rates related to the extra events just fitted the above re-corrections of the ECH events.

Eventually, we found that the coriander paralogs had a corrected Ks distribution peaking at 0.42(±0.23) and 0.50(±0.28) for alpha and beta events, respectively. Assuming that the ECH occurred 115–130 Mya with Ks distribution peaking at 1.053 (Vekemans et al., 2002; Jiao et al., 2012), these two events have occurred 45-52, 54-61 Mya. Notably, the lettuce hexaploidy-produced paralogs had a corrected Ks distribution peaking at 0.66(±0.20) (73-82Mya), showing that the Asteraceae-common hexaploidy (ACH) was older than the two paleo-tetraploidization events in coriander. In addition, the coriander-carrot split was inferred to have occurred 24–28 mya, and they split 98-111 mya from lettuce (Supplementary Fiugre 11c,d).

6.2.9 Positive selection analysis

Positive selection means that in a single-copy gene family, a certain gene is affected by environmental or human factors during the evolution process. In order to adapt to the environment changing, a centain gene occur non-synonymous mutation at the amino acid level. The Ka/Ks value was calculated to detect the probability of being positively selected. *C. sativum* is the foreground branch of the positive selection analysis, and the other 12 representative species (*L. sativa, O. sativa, A. thaliana, P. trichocarpa, S. indicum, D. carota, S. tuberosum, A. chinensis, C. canephora, B. rapa, M. truncatula, N. nucifera*) as a background branch. By likelihood ratio detection, 81 positively selected candidate genes were identified in *C. sativum* (p-value<0.01, FDR < 0.05).

6.2.10 Analysis of functional genes

Terpenoid biosynthesis pathways analyses

The pathways of terpenoid backbone biosynthesis manily contained eight subpathways in Arabidopsis according to the KEGG. We firstly searched the genes of Arabidopsis from these subpathways, including 23 genes in Sesquiterpenoid and triterpenoid biosynthesis, 34 genes in Steroid biosynthesis, 40 genes in N-Glycan biosynthesis, 6 genes in Zeatin biosynthesis, 5 genes in Monoterpenoid biosynthesis, 9 genes in Diterpenoid biosynthesis, 20 genes in Carotenoid biosynthesis, and 22 genes in

Ubiquinone and other terpenoid-quinone biosynthesis (Supplementary Table 44). Then, we inferred homologous genes of these pathways with A. thaliana in other 6 representative species using Blast program (e-value<1e-5, identify>50%, score >200). The results showed that almost every node in the regulatory pathway has one or more gene copy among all the 7 species. These eight subpathways contained 801 genes in the 7 species (Supplementary Table 45).

Furthermore, we detected the expression level of these candidate genes in *C. sativum* by RNA-Seq with three replications. The samples used for RNA-Seq were not only from the different tissues, including root, stem, leaf, and flower, but also contained the different development periods, including 30, 60, and 90 days after sowing of *C. sativum*. We detected the expression for 113 genes of eight subpathways in *C. sativum* (Supplementary Table 46-48, Supplementary Figure 21,22). In conclusion, this study systematically analyzed the gene copy and gene expression of this regulatory pathway in coriander, which laid a foundation for the better study of the gene function of the pathway in coriander.

TPS gene family analyses

The genes in the gene family are key enzymes in the regulatory network of synthetic terpenoids and play important regulatory roles in the synthesis of various terpenoids, including monoene, diene, sesquiterpene and polyene (Aubourg et al., 2002; Chen et al., 2011; Yahyaa et al., 2015). The scent is a volatile oil substance, and the main constituents are these olefinic materials (Martin et al., 2010; Savoi et al., 2016).

We compared the ratio of the number of TPS genes to the whole genome genes in each species. It was found that there was no obvious distribution in the classification, but there was obvious species preference. At the same time, we also analyzed the ratio of the number of TPS genes in each species to the number of candidate TPS genes, and found similar rules to the former (Figure 6).

For *C. sativum*, the vast majority of TPS genes were distributed in the TPS-a and TPS-b groups, 12 and 11 respectively. The number of genes in the TPS-c, TPS-e, TPS-f and TPS-g groups was small, followed by 2, 2, 1 and 1. Previous studies have shown that TPS-a genes mainly encode cadinene synthase isozyme, TPS-b genes mainly encode Myrcene synthase, TPS-c genes mainly encode Ent-copalyl diphosphate synthase, and

TPS-e genes mainly encode Ent-kaur-16-ene synthase, the TPS-f gene mainly encodes S-linalool synthase, and the TPS-g gene mainly encodes Linalool synthase and Myrcene synthase (Aubourg et al., 2002). In this study, we conducted the RNA-Seq analyses in different tissues, including root, stem, leaf, and flower (Figure 7b). The results showed than there were more genes with the higher expression level than other 3 tissues. Furthermore, we also conducted the RNA-Seq analyses in different development periods, including 30d, 60d, and 90d after sowing of C. sativum (Figure 7b). We found that several TPS genes were still high expressed in these 3 development periods, such as *Cs02G02594.1*, *Cs06G00661.1*. However, there were also 11 genes with no expression among all three periods. Interestingly, we found that the *Cs06G00661.1* gene, which had high expression level whenever in different tissues and different development periods. It was belonged to the Tps-g group, so it may play important roles in encoding Linalool synthase and Myrcene synthase. Therefore, this study laid the foundation for further study of the gene function in each TPS group of coriander

References

Anders, S., and Huber, W. (2010). Differential expression analysis for sequence count data. Genome Biol 11:R106.

Anders, S., Pyl, P.T., and Huber, W. (2015). HTSeq--a Python framework to work with high-throughput sequencing data. Bioinformatics 31:166-169.

Aubourg, S., Lecharny, A., and Bohlmann, J. (2002). Genomic analysis of the terpenoid synthase (AtTPS) gene family of Arabidopsis thaliana. Mol Genet Genomics 267:730-745.

Bairoch, A. (2005). From sequences to knowledge, the role of the Swiss-Prot component of UniProt. Molecular & Cellular Proteomics 4:S2-S2.

Bairoch, A., and Apweiler, R. (2000). The SWISS-PROT protein sequence database and its supplement TrEMBL in 2000. Nucleic Acids Research 28:45-48.

Bao, W., Kojima, K.K., and Kohany, O. (2015). Repbase Update, a database of repetitive elements in eukaryotic genomes. Mob DNA 6:11.

Benson, G. (1999). Tandem repeats finder: a program to analyze DNA sequences. Nucleic Acids Res 27:573-580.

Birney, E., Clamp, M., and Durbin, R. (2004). GeneWise and Genomewise. Genome Res 14:988-995.

Camacho, C., Coulouris, G., Avagyan, V., Ma, N., Papadopoulos, J., Bealer, K., and Madden, T.L. (2009). BLAST+: architecture and applications. BMC Bioinformatics 10:421.

Chan, P.P., and Lowe, T.M. (2019). tRNAscan-SE: Searching for tRNA Genes in

Genomic Sequences. Methods Mol Biol 1962:1-14.

Chen, F., Tholl, D., Bohlmann, J., and Pichersky, E. (2011). The family of terpene synthases in plants: a mid-size family of genes for specialized metabolism that is highly diversified throughout the kingdom. Plant J 66:212-229.

De Bie, T., Cristianini, N., Demuth, J.P., and Hahn, M.W. (2006). CAFE: a computational tool for the study of gene family evolution. Bioinformatics 22:1269-1271.

Edgar, R.C., and Myers, E.W. (2005). PILER: identification and classification of genomic repeats. Bioinformatics 21 Suppl 1:i152-158.

Etherington, G.J., Ramirez-Gonzalez, R.H., and MacLean, D. (2015). bio-samtools 2: a package for analysis and visualization of sequence and alignment data with SAMtools in Ruby. Bioinformatics 31:2565-2567.

Fischer, S., Brunk, B.P., Chen, F., Gao, X., Harb, O.S., Iodice, J.B., Shanmugam, D., Roos, D.S., and Stoeckert, C.J., Jr. (2011). Using OrthoMCL to assign proteins to OrthoMCL-DB groups or to cluster proteomes into new ortholog groups. Curr Protoc Bioinformatics Chapter 6:Unit 6 12 11-19.

Guindon, S., Lethiec, F., Duroux, P., and Gascuel, O. (2005). PHYML Online--a web server for fast maximum likelihood-based phylogenetic inference. Nucleic Acids Res 33:W557-559.

Haas, B.J., Delcher, A.L., Mount, S.M., Wortman, J.R., Smith, R.K., Hannick, L.I., Maiti, R., Ronning, C.M., Rusch, D.B., Town, C.D., et al. (2003). Improving the Arabidopsis genome annotation using maximal transcript alignment assemblies. Nucleic Acids Research 31:5654-5666.

Haas, B.J., Salzberg, S.L., Zhu, W., Pertea, M., Allen, J.E., Orvis, J., White, O., Buell, C.R., and Wortman, J.R. (2008). Automated eukaryotic gene structure annotation using EVidenceModeler and the Program to Assemble Spliced Alignments. Genome Biol 9:R7.

Hansen, K.D., Irizarry, R.A., and Wu, Z. (2012). Removing technical variability in RNA-seq data using conditional quantile normalization. Biostatistics 13:204-216.

Jo, H., and Koh, G. (2015). Faster single-end alignment generation utilizing multi-thread for BWA. Biomed Mater Eng 26 Suppl 1:S1791-1796.

Kent, W.J. (2002). BLAT -- The BLAST-Like Alignment Tool. Genome Research 4:656-664.

Kim, D., Langmead, B., and Salzberg, S.L. (2015). HISAT: a fast spliced aligner with low memory requirements. Nat Methods 12:357-360.

Korf, I. (2004). Gene finding in novel genomes. BMC Bioinformatics 5:59.

Kumar, S., Stecher, G., Suleski, M., and Hedges, S.B. (2017). TimeTree: A Resource for Timelines, Timetrees, and Divergence Times. Mol Biol Evol 34:1812-1819.

Li, H., Handsaker, B., Wysoker, A., Fennell, T., Ruan, J., Homer, N., Marth, G., Abecasis, G., Durbin, R., and Genome Project Data Processing, S. (2009). The Sequence Alignment/Map format and SAMtools. Bioinformatics 25:2078-2079.

Marcais, G., and Kingsford, C. (2011). A fast, lock-free approach for efficient parallel counting of occurrences of k-mers. Bioinformatics 27:764-770.

Martin, D.M., Aubourg, S., Schouwey, M.B., Daviet, L., Schalk, M., Toub, O., Lund, S.T., and Bohlmann, J. (2010). Functional annotation, genome organization and phylogeny of the grapevine (Vitis vinifera) terpene synthase gene family based on genome assembly, FLcDNA cloning, and enzyme assays. BMC Plant Biol 10:226.

Mulder, N.J., and Apweiler, R. (2008). The InterPro database and tools for protein domain analysis. Curr Protoc Bioinformatics Chapter 2:Unit 2 7.

Nawrocki, E.P., and Eddy, S.R. (2013). Infernal 1.1: 100-fold faster RNA homology searches. Bioinformatics 29:2933-2935.

Ogata, H., Goto, S., Sato, K., Fujibuchi, W., Bono, H., and Kanehisa, M. (1999). KEGG: Kyoto Encyclopedia of Genes and Genomes. Nucleic Acids Research 27:29-34.

Parra, G., Bradnam, K., and Korf, I. (2007). CEGMA: a pipeline to accurately annotate core genes in eukaryotic genomes. Bioinformatics 23:1061-1067.

Price, A.L., Jones, N.C., and Pevzner, P.A. (2005). De novo identification of repeat families in large genomes. Bioinformatics 21 Suppl 1:i351-358.

Retief, J.D. (2000). Phylogenetic analysis using PHYLIP. Methods Mol Biol 132:243-258.

Savoi, S., Wong, D.C., Arapitsas, P., Miculan, M., Bucchetti, B., Peterlunger, E., Fait, A., Mattivi, F., and Castellarin, S.D. (2016). Transcriptome and metabolite profiling reveals that prolonged drought modulates the phenylpropanoid and terpenoid pathway in white grapes (Vitis vinifera L.). BMC Plant Biol 16:67.

Seppey, M., Manni, M., and Zdobnov, E.M. (2019). BUSCO: Assessing Genome Assembly and Annotation Completeness. Methods Mol Biol 1962:227-245.

Shen, S., Park, J.W., Lu, Z.X., Lin, L., Henry, M.D., Wu, Y.N., Zhou, Q., and Xing, Y. (2014). rMATS: robust and flexible detection of differential alternative splicing from replicate RNA-Seq data. Proc Natl Acad Sci U S A 111:E5593-5601.

Shimada, M.K., and Nishida, T. (2017). A modification of the PHYLIP program: A solution for the redundant cluster problem, and an implementation of an automatic bootstrapping on trees inferred from original data. Mol Phylogenet Evol 109:409-414.

Stamatakis, A. (2014). RAxML version 8: a tool for phylogenetic analysis and post-analysis of large phylogenies. Bioinformatics 30:1312-1313.

Stanke, M., and Morgenstern, B. (2005). AUGUSTUS: a web server for gene prediction in eukaryotes that allows user-defined constraints. Nucleic Acids Res 33:W465-467.

Talavera, G., and Castresana, J. (2007). Improvement of phylogenies after removing divergent and ambiguously aligned blocks from protein sequence alignments. Syst Biol 56:564-577.

Tarailo-Graovac, M., and Chen, N. (2009). Using RepeatMasker to identify repetitive elements in genomic sequences. Curr Protoc Bioinformatics Chapter 4:Unit 4 10.

Trapnell, C., Williams, B.A., Pertea, G., Mortazavi, A., Kwan, G., van Baren, M.J., Salzberg, S.L., Wold, B.J., and Pachter, L. (2010). Transcript assembly and quantification by RNA-Seq reveals unannotated transcripts and isoform switching during cell differentiation. Nat Biotechnol 28:511-515.

Wang, J., Sun, P., Li, Y., Liu, Y., Yang, N., Yu, J., Ma, X., Sun, S., Xia, R., Liu, X., et al. (2017a). An overlooked paleo-tetraploidization in Cucurbitaceae. Molecular Biology and Evolution:msx242-msx242.

Wang, J., Sun, P., Li, Y., Liu, Y., Yu, J., Ma, X., Sun, S., Yang, N., Xia, R., and Lei, T. (2017b). Hierarchically aligning 10 legume genomes establishes a family-level genomics platform. Plant physiology 174:284.

Wang, J., Yu, J., Sun, P., Li, Y., Xia, R., Liu, Y., Ma, X., Yu, J., Yang, N., and Lei, T.

(2016a). Comparative Genomics Analysis of Rice and Pineapple Contributes to Understand the Chromosome Number Reduction and Genomic Changes in Grasses. Frontiers in Genetics 7.

Wang, X., Guo, H., Wang, J., Lei, T., Liu, T., Wang, Z., Li, Y., Lee, T.H., Li, J., and Tang, H. (2016b). Comparative genomic de-convolution of the cotton genome revealed a decaploid ancestor and widespread chromosomal fractionation. New Phytologist 209:1252-1263.

Wang, X., Shi, X., Hao, B., Ge, S., and Luo, J. (2005). Duplication and DNA segmental loss in the rice genome: implications for diploidization. New Phytologist 165:937-946.

Wang, X., Shi, X., Li, Z., Zhu, Q., Kong, L., Tang, W., Ge, S., and Luo, J. (2006). Statistical inference of chromosomal homology based on gene colinearity and applications to Arabidopsis and rice. BMC bioinformatics 7:447.

Wang, X., Wang, J., Jin, D., Guo, H., Lee, T.H., Liu, T., and Paterson, A.H. (2015). Genome Alignment Spanning Major Poaceae Lineages Reveals Heterogeneous Evolutionary Rates and Alters Inferred Dates for Key Evolutionary Events. Molecular plant 8:885-898.

Wang, Y., Tang, H., Debarry, J.D., Tan, X., Li, J., Wang, X., Lee, T., Jin, H., Marler, B., and Guo, H. (2012). MCScanX: a toolkit for detection and evolutionary analysis of gene synteny and collinearity. Nucleic Acids Research 40:e49-e49.

Waterhouse, R.M., Seppey, M., Simao, F.A., and Zdobnov, E.M. (2019). Using BUSCO to Assess Insect Genomic Resources. Methods Mol Biol 1858:59-74.

Xie, C., Mao, X., Huang, J., Ding, Y., Wu, J., Dong, S., Kong, L., Gao, G., Li, C.Y., and Wei, L. (2011). KOBAS 2.0: a web server for annotation and identification of enriched pathways and diseases. Nucleic Acids Res 39:W316-322.

Xu, Z., and Wang, H. (2007). LTR_FINDER: an efficient tool for the prediction of full-length LTR retrotransposons. Nucleic Acids Res 35:W265-268.

Yahyaa, M., Matsuba, Y., Brandt, W., Doron-Faigenboim, A., Bar, E., McClain, A., Davidovich-Rikanati, R., Lewinsohn, E., Pichersky, E., and Ibdah, M. (2015). Identification, Functional Characterization, and Evolution of Terpene Synthases from a Basal Dicot. Plant Physiol 169:1683-1697.

Yang, Z. (1997). PAML: a program package for phylogenetic analysis by maximum likelihood. Comput Appl Biosci 13:555-556.

Yang, Z. (2007). PAML 4: phylogenetic analysis by maximum likelihood. Mol Biol Evol 24:1586-1591.

Young, M.D., Wakefield, M.J., Smyth, G.K., and Oshlack, A. (2010). Gene ontology analysis for RNA-seq: accounting for selection bias. Genome Biol 11:R14.
